# Mechanism of spliceosome termination

**DOI:** 10.1038/s41586-026-11101-6

**Published:** 2026-10-07

**Authors:** Vytaute Boreikaite, Rupert Faraway, Matthias K. Vorländer, Alexander W. Phillips, Leonie Opitz, Moritz Wanke, George Yakoub, Gerald Raffl, Laura Fin, Román González-Prieto, Martijn S. Luijsterburg, Stefan L. Ameres, Clemens Plaschka

**Affiliations:** 1 Research Institute of Molecular Pathology (IMP), Vienna BioCenter (VBC), Vienna, Austria; 2 Max Perutz Labs, Vienna BioCenter (VBC), Vienna, Austria; 3 University of Vienna, Max Perutz Labs, Vienna, Austria; 4 Department of Human Genetics, Leiden University Medical Center, Leiden, The Netherlands; 5 Vienna BioCenter PhD Program, Doctoral School of the University of Vienna and Medical University of Vienna, Vienna, Austria; 6 Andalusian Centre for Regenerative Medicine and Molecular Biology (CABIMER), Universidad de Sevilla-CSIC–Universidad Pablo de Olavide-Junta de Andalucía, Sevilla, Spain; 7 Departament of Cell Biology, Faculty of Biology, University of Sevilla, Sevilla, Spain

**Keywords:** Cryoelectron microscopy, RNA splicing

## Abstract

After excising an intron from pre-mRNA, the spliceosome remains trapped in a non-productive complex bound to the intron^1–5^. Termination of this complex is critical for spliceosome recycling and intron decay^6^, but the mechanism remains unknown. Here we present cryo-electron microscopy structures of human spliceosomes at two sequential stages of termination. First, the RNA helicases DHX15 and Aquarius unwind the RNA active site of the spliceosome, releasing bound components including U2 snRNA and extracting the buried intron–lariat branch point. The branch point is then debranched by the spliceosome-tethered enzyme DBR1, generating the previously unknown debranched intron spliceosome. This state recruits the RNA helicase DHX35 with its co-factors GPATCH1–WDR83, assisted by YJU2B. DHX35 ejects the debranched intron from the U6 snRNA–5′ splice site duplex, driving spliceosome disassembly and intron turnover. In defective spliceosomes stalled on aberrant introns, YJU2B partners with LENG1 to guide DHX35–GPATCH1–WDR83 for termination through spliceosome quality control. Together, we reveal the mechanism of regular spliceosome termination and its parallels with spliceosome quality control, ensuring accurate and efficient pre-mRNA splicing.

## Abstract (web summary)

A coordinated sequence of RNA helicases and associated factors dismantles normal and defective spliceosome complexes to enable intron degradation and ensure accurate gene expression.

## Main

**Fig. 1:**
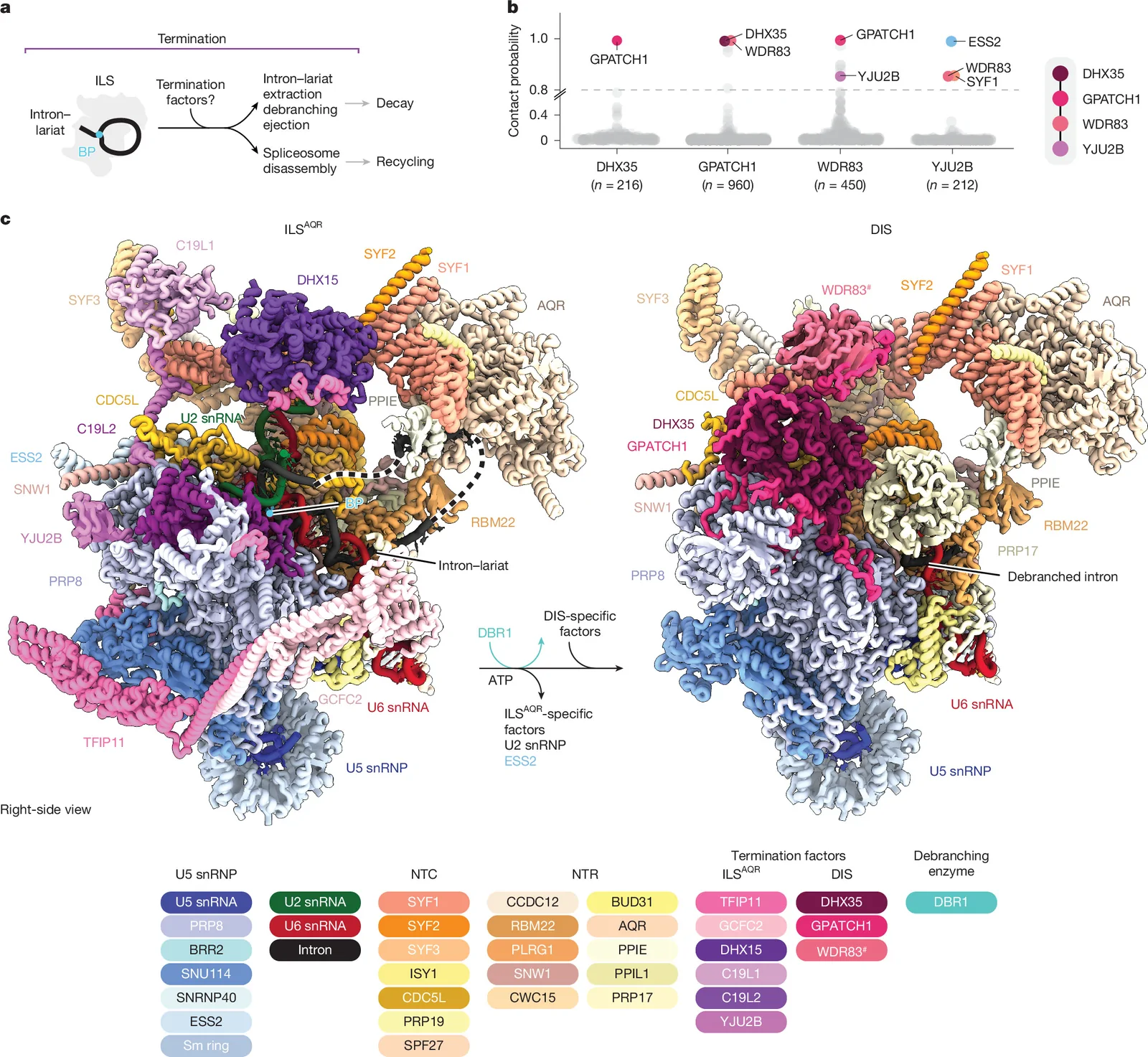
Structures of terminating human spliceosomes. **a**, Schematic of terminal splicing events. **b**, Identification of candidate termination factors (DHX35, GPATCH1, WDR83 and YJU2B) in a human proteome-wide in silico interaction screen^20^. The ‘*n*’ refers to the number of candidate interactors reported for each protein. The inset on the right summarizes the predicted chain of protein–protein interactions. Dashed line indicates an arbitrary cut-off of the contact probability score (0.8). **c**, Cryo-EM structures of the ILS^AQR^ and the DIS. Proteins and RNAs are coloured according to the scheme below, which is used throughout. The orientation of the WDR83 WD40 domain is not assigned owing to the low local resolution (marked with ‘#’).

Pre-mRNA splicing is carried out by the spliceosome, a dynamic multi-megadalton ribonucleoprotein complex^7,8^. The spliceosome assembles anew on each pre-mRNA intron to catalyse its excision as a branched intron–lariat and to ligate the flanking exons^7,8^. Although the ligated mRNA is released after catalysis, the intron–lariat remains bound within the RNA-based active site of the spliceosome, forming a stable complex called the intron–lariat spliceosome (ILS)^1^. To sustain subsequent rounds of pre-mRNA splicing, the ILS must be disassembled to allow the recycling of its constituent proteins and small nuclear RNAs (snRNAs)^9,10^. In addition, to maintain RNA homeostasis, the intron–lariat must be ejected from the spliceosome, debranched at its branch point (BP) adenosine, and eventually degraded^6,11,12^. These combined events—intron–lariat ejection, debranching and spliceosome disassembly—are referred to as spliceosome termination^13^ (Fig. 1a).

Recent studies have uncovered the initial recognition of the ILS by four dedicated termination factors and the DEAH-box RNA helicase DHX15 (refs. ^2–5^), but subsequent termination steps remain unclear. In the available ILS structures^2–5^, the excised intron–lariat and its BP remain sequestered within the RNA network of the spliceosome. Intron–lariat debranching by the essential enzyme DBR1 has been proposed to occur on the spliceosome^14–16^, but this would require extraction of the intron–lariat BP from the RNA network before intron ejection through as-yet-unknown mechanisms. Subsequently, the intron must be ejected from the spliceosome for decay, and the spliceosome itself needs to disassemble. These terminal splicing events probably involve extensive remodelling of the spliceosome, possibly through RNA helicase action on the spliceosomal RNA network^17^. However, the mechanisms of intron–lariat extraction, debranching and ejection from the spliceosome, their coupling to spliceosome disassembly, and the participating factors at each step remain unclear (Fig. 1a).

Here we combined cryo-electron microscopy (cryo-EM) of endogenous human spliceosomes with biochemistry, RNA sequencing and genetics to reveal the mechanisms of spliceosome termination. We define the roles of the RNA helicases DHX15, Aquarius (AQR) and DHX35 in termination, identify a new splicing intermediate—the debranched intron spliceosome (DIS)—and reveal how shared and context-specific factors terminate both regular and defective spliceosomes to sustain pre-mRNA splicing.

## Identification of spliceosome termination factors

**Extended Data Fig. 1:**
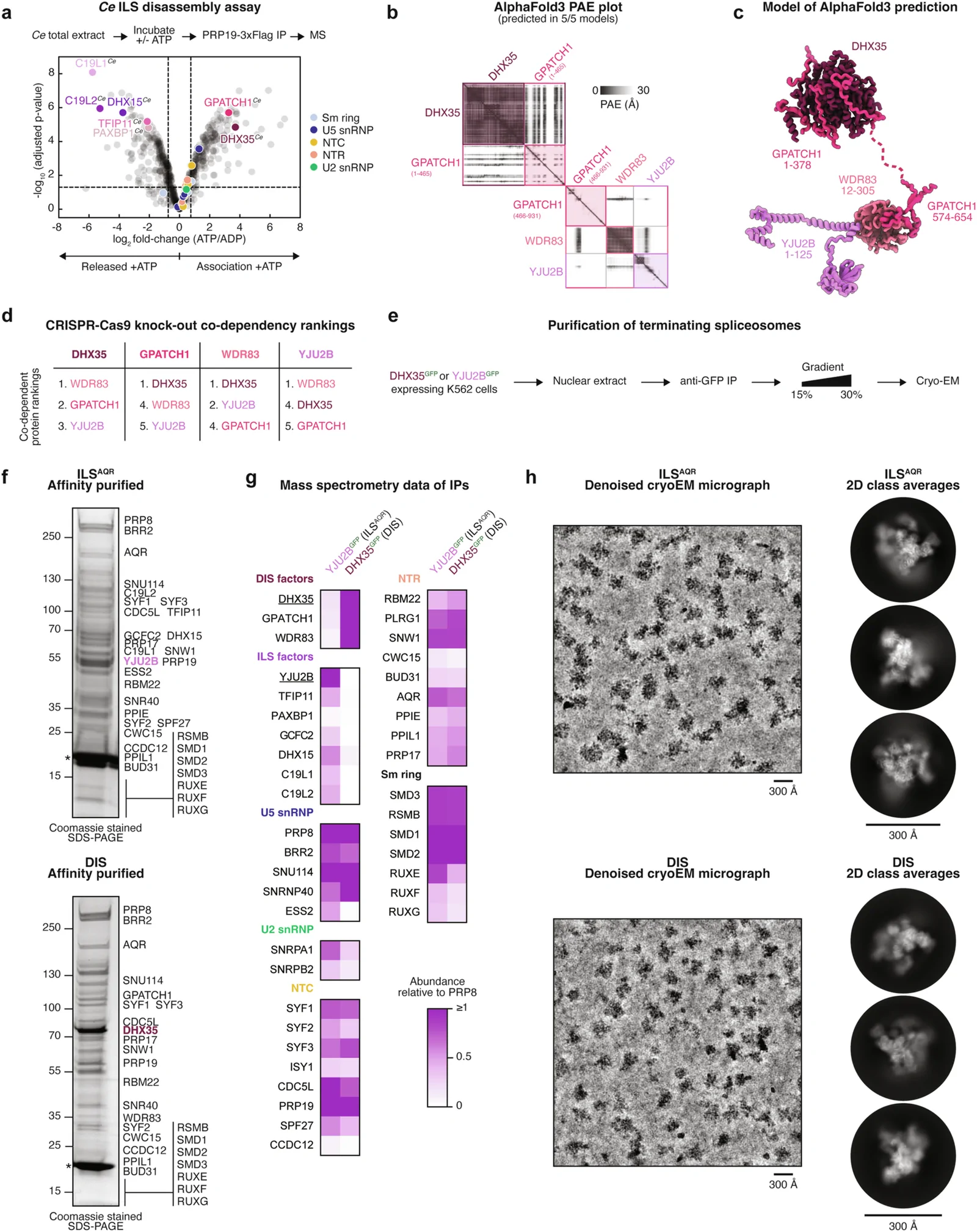
Biochemical and cryo-EM analysis of terminating spliceosomes. **a**. *C. elegans* ILS remodeling assay in whole nematode extract. Top: Experiment schematic of the assay. Bottom: Results of the remodelling assay measured by quantitative mass spectrometry and shown in a volcano plot. Nematode extract was incubated with ATP, after which spliceosomes were purified by a pulldown on the endogenously 3xFlag-tagged NTC subunit PRP19 (ref. ^2^). Although PRP19 is generally common to all catalytic and post-catalytic spliceosome states, the ILS is the dominant spliceosome state in *C. elegans* extract. In the experiment, the known termination factors are released after the ATP treatment (DHX15, PAXBP1, C19L1, C19L2, TFIP11), while the later termination factors (DHX35, GPATCH1) associate. Other spliceosomal subunits do not significantly change in abundance. This suggests that in extracts the ILS remodels but does not completely disassemble upon ATP treatment. Differences between groups were statistically evaluated using limma with a moderated two-sided t-test. The multiple testing correction used the Benjamini-Hochberg procedure. The horizontal dashed line marks a threshold of p-value of 0.05, the vertical dashed lines denote a log_2_-fold change threshold of one (two-fold change on a linear scale). **b**. Predicted alignment error (PAE) plot of the DHX35–GPATCH1–WDR83–YJU2B complex prediction from AlphaFold3. Each binary interaction was confidently predicted in five out of five models. **c**. Model of the DHX35–GPATCH1–WDR83–YJU2B complex prediction from AlphaFold3. **d**. Table of the co-dependency rankings of DHX35, GPATCH1, WDR83 and YJU2B in each other’s CRISPR-Cas9 knock-out viability screens, according to the DepMap database^24^ (as of June 2026). Co-dependency means that the four proteins are required for growth in the same set of cancer cell lines, indicating that they may function in a common cellular pathway. **e**. Experiment schematic for the endogenous purification of DHX35- and YJU2B-containing spliceosomes. **f**. SDS-PAGE analyses of the immuno-purified samples of the YJU2B- (top; ILS^AQR^) and DHX35- (bottom; DIS) containing spliceosomes. The bait protein for either experiment is written in bold. The molecular weight in kilodalton (kDa) of the molecular weight marker is indicated to the left of each gel. The asterisk marks 3C protease, which was used to cleave GFP from the bait protein and thereby elute associated spliceosomes from anti-GFP nanobody beads. Both experiments were performed four times. **g**. Mass spectrometry analyses of YJU2B-GFP (ILS^AQR^) and DHX35-GFP (DIS) immuno-purified spliceosomes. Protein abundances were normalized to the core U5 snRNP protein PRP8. DHX35-bound spliceosomes contain significantly less of the ILS-specific factors, the U2 snRNP, and ESS2, in comparison to YJU2B-bound spliceosomes. In contrast, YJU2B-bound spliceosomes lack DHX35, GPATCH1, and WDR83, but contain the ILS-specific factors. The differences observed by mass spectrometry are also reflected in the cryo-EM structures of the two samples. **h**. Cryo-EM analyses of ILS^AQR^ (top) and DIS (bottom). Representative denoised cryo-EM micrographs (left) were selected from 53,418 and 12,277 micrographs of ILS^AQR^ and DIS samples, respectively. Representative cryo-EM 2D class averages (right) are also shown.

We have previously shown that the *Caenorhabditis elegans* ILS is the predominant spliceosome state in whole-worm extract and that it associates with the RNA helicase DHX15 (ref. ^2^). Adding ATP to these extracts activates DHX15 to initiate spliceosome termination. Here we purified these ATP-treated terminating spliceosomes for mass spectrometry analysis, aiming to capture later states of termination. Compared with the ILS, the resultant complexes were depleted of the five known termination factors (TFIP11, PAXBP1, C19L1, C19L2 and DHX15) but significantly enriched in DHX35 and GPATCH1 (Extended Data Fig. 1a and Supplementary Table 1), two factors known to associate with the spliceosome at the end of pre-mRNA splicing^18,19^. This suggested that metazoan DHX35 and GPATCH1 act in terminal splicing events, after ATP-dependent remodelling of the ILS.

To identify additional termination factors, we analysed the predicted protein–protein interactions of DHX35 and GPATCH1 within the human proteome^20^ (Fig. 1b). Of note, DHX35 and GPATCH1 were predicted as each other’s most confident interactors (Extended Data Fig. 1b,c), consistent with their reported interactions in fission yeast and humans^18,21,22^. GPATCH1 was further predicted to bind to WDR83 (Extended Data Fig. 1b,c), in agreement with previous biochemical reports in fission yeast^21–23^. WDR83 was itself predicted to interact with an uncharacterized protein YJU2B (Extended Data Fig. 1c), which in turn was predicted to bind to other spliceosomal proteins (ESS2 and SYF1). The four proteins also scored among each other’s top co-dependent proteins in human CRISPR–Cas9 knockout screens^24,25^ (Extended Data Fig. 1d), consistent with a common pathway. Together, these data suggest a chain of interactions among DHX35, GPATCH1, WDR83 and YJU2B (Fig. 1b and Extended Data Fig. 1b–d), with putative roles in spliceosome termination.

## Structures of terminating spliceosomes

**Extended Data Fig. 2:**
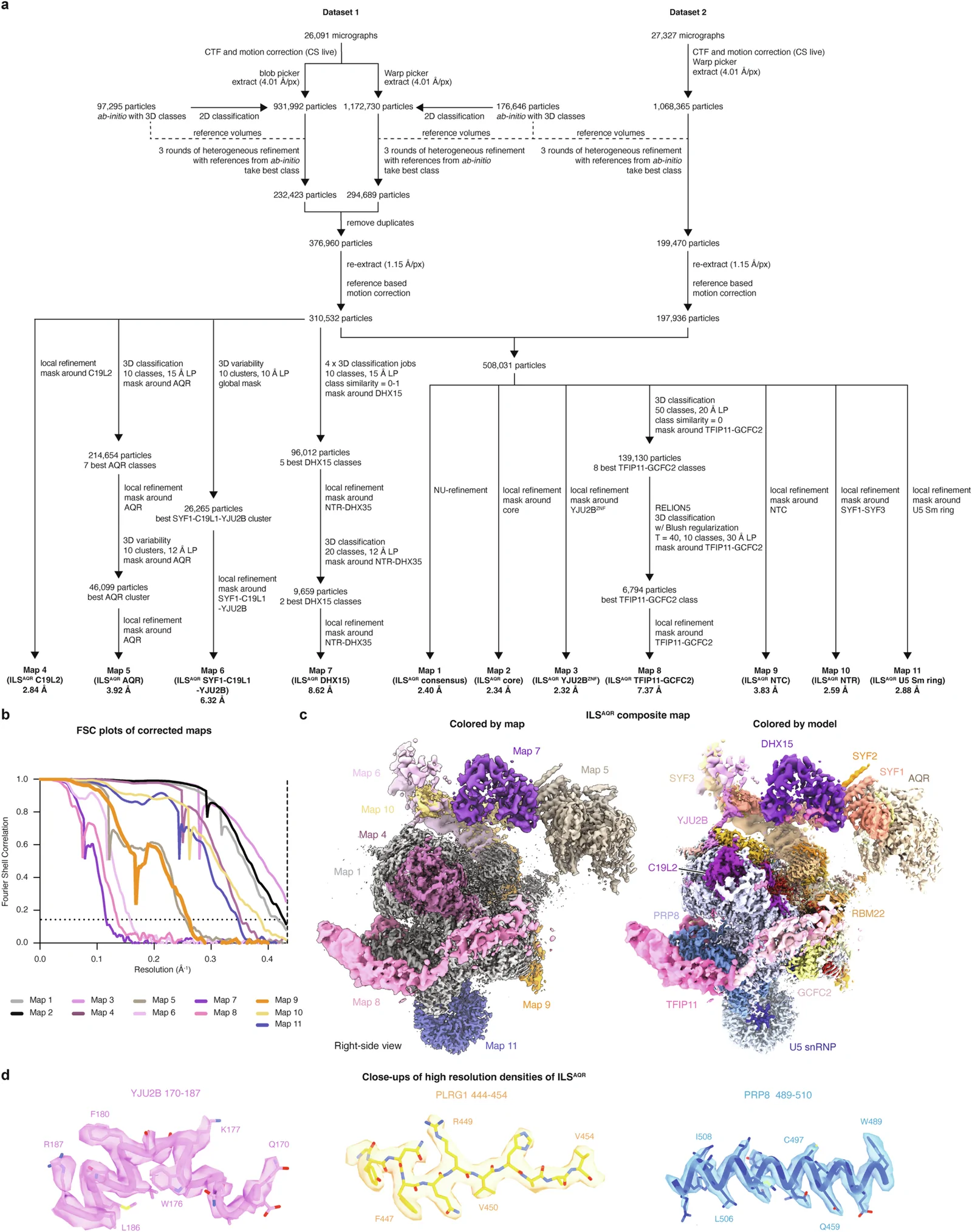
Overview of cryo-EM data processing of ILS^AQR^. **a**. Schematic representation of cryo-EM data processing of the two ILS^AQR^ datasets. Apart from particle picking done in Warp^66^ and certain 3D classification jobs performed in RELION5 (refs. ^67,77^), all processing steps were performed in CryoSPARC^65^. 11 different maps were used to generate a composite map of the ILS^AQR^ complex. At 2.4 Å resolution, the core of the ILS^AQR^ is the highest resolution map of an assembled human spliceosome published to date. See Materials and Methods for details. **b**. Fourier shell correlation (FSC) plots of correlation-corrected maps of ILS ^AQR^ described in panel a. The gold standard correlation value of 0.143 is indicated with a black dotted line. The Nyquist limit of 2.3 Å, or 0.43 Å^−1^, is marked with a dotted line. The colour legend is shown under the plot. The plots were generated as part of refinement jobs in CryoSPARC. **c**. Composite map of ILS^AQR^ generated by combining the maps described in panel a and coloured according to either the contributing map (left) or the modelled proteins and RNA elements (right). **d**. Example snapshots of high-resolution densities of ILS^AQR^ overlayed with the corresponding protein models.

**Extended Data Fig. 3:**
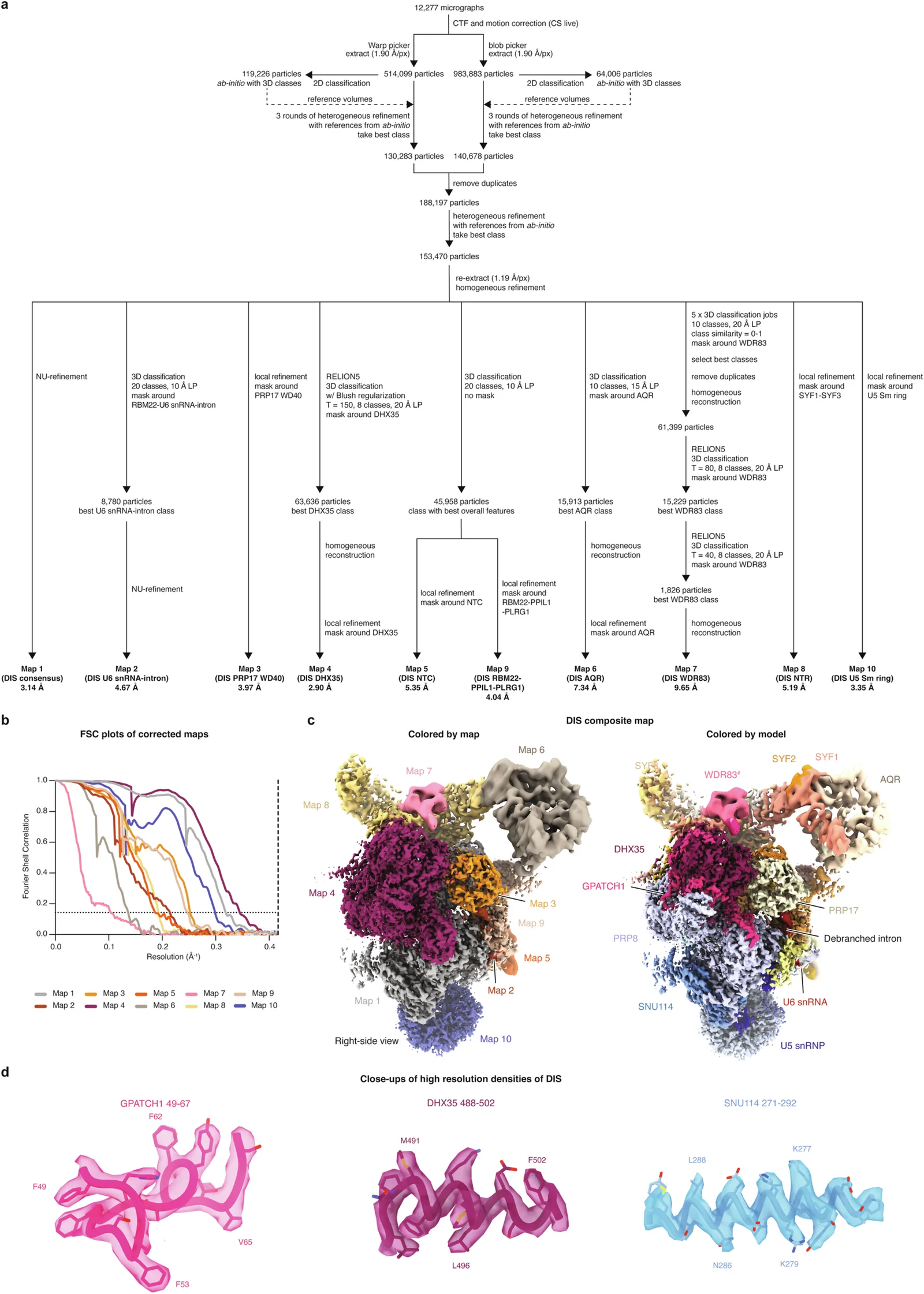
Overview of cryo-EM data processing of DIS. **a**. Schematic representation of cryo-EM data processing of the DIS dataset. Apart from particle picking done in Warp^66^ and certain 3D classification jobs performed in RELION5 (refs. ^67,77^), all processing steps were performed in CryoSPARC^65^. 10 different maps were used to generate a composite map of the DIS complex. See Materials and Methods for details. **b**. Fourier shell correlation (FSC) plots of correlation-corrected maps of DIS described in panel a. The gold standard correlation value of 0.143 is indicated with a black dotted line. The Nyquist limit of 2.38 Å, or 0.42 Å^−1^, is marked with a dotted line. The colour legend in shown under the plot. The plots were generated as part of refinement jobs in CryoSPARC. **c**. Composite map of ILS^AQR^ generated by combining the maps described in panel a and coloured according to either the contributing map (left) or the modelled proteins and RNA elements (right). **d**. Example snapshots of high-resolution densities of ILS^AQR^ overlayed with the corresponding protein models.

To investigate the mechanism of spliceosome termination, we purified endogenous spliceosomes from the nuclear extract of human K562 cells carrying a GFP tag on DHX35 or YJU2B (Extended Data Fig. 1e,f), placed at either end of the predicted interaction chain. Mass spectrometry revealed that these two spliceosomes differed substantially in protein composition, indicating two distinct states (Extended Data Fig. 1g and Supplementary Table 2). We next determined cryo-EM structures of YJU2B-containing and DHX35-containing spliceosomes, at nominal resolutions of 2.4 Å and 3.2 Å, respectively (Fig. 1c, Extended Data Figs. 1h, 2 and 3 and Supplementary Table 3).

The YJU2B-containing spliceosome contained the excised intron–lariat, YJU2B and the five known termination factors, including the RNA helicase DHX15, indicating that this spliceosome represents an ILS-like complex (Fig. 1c). Unexpectedly, the intron–lariat RNA was bound by AQR, which acts on the intron during spliceosome activation and remains in the ILS as part of the NineTeen related complex (NTR)^26,27^. We refer to this complex as ILS^AQR^.

By contrast, the DHX35-bound spliceosome contained DHX35, GPATCH1 and WDR83 (Fig. 1b) but lacked an RNA active site, the U2 snRNP, ESS2, the five known termination factors and YJU2B (Fig. 1c). Furthermore, it contained an excised and debranched intron, tethered to the spliceosome through a partial U6 snRNA–5′ splice site (SS) duplex. Because of this notable feature, we refer to this new state as the DIS. The DIS structure indicates that intron debranching occurs predominantly before intron ejection, consistent with previous reports^14–16^ (Fig. 1c).

Together, the composition and conformation of these complexes suggest that ILS^AQR^ and DIS form sequentially during termination, representing previously unknown terminal intermediates of pre-mRNA splicing.

## BP extraction

**Fig. 2:**
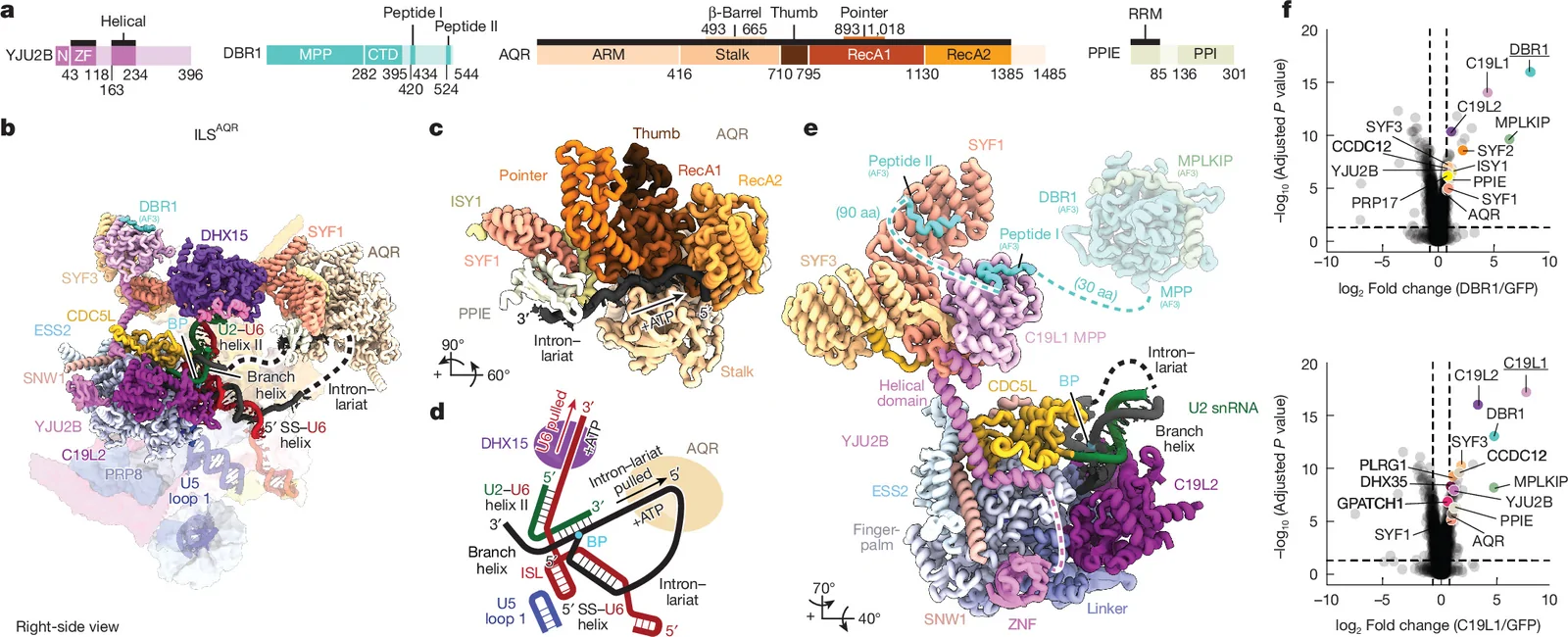
Insights into intron–lariat extraction and debranching on the spliceosome. **a**, Domain organization of YJU2B, DBR1, AQR and PPIE. The regions modelled in the ILS^AQR^ structure are marked with black lines. CTD, C-terminal domain; N, N-terminal extension; PPI, protein proline isomerase domain; ZF, zinc finger domain. **b**, ILS^AQR^ structure with termination factors (TFIP11, C19L1, C19L2, YJU2B and DHX15), their direct interactors and key RNA elements highlighted. The remainder of the complex is shown as a transparent surface. DBR1 peptide I (DBR1 (AF3)) is modelled based on an AlphaFold3 prediction and validated by biochemical and genetic data (Extended Data Fig. 4). **c**, AQR binds to intron–lariat RNA, which is guided to AQR by PPIE. The AQR domain colours are defined in panel **a**. The arrow marks the direction of ATP-dependent 5′-to-3′-directed pulling of the intron–lariat RNA by AQR. **d**, Schematic of the ILS^AQR^ RNA active site, with the arrows indicating the different pulling directions by each helicase. **e**, Interactions of YJU2B and DBR1 with ILS^AQR^. PRP8 is coloured according to its domains. DBR1 peptides I and II (marked with (AF3)) are modelled based on AF3 predictions and our biochemical and genetic data. The DBR1 MPP domain and interacting MPLKIP regions are modelled based on an AF3 prediction (marked with (AF3)). aa, amino acid. **f**, Proximity labelling using TurboID fused to DBR1 (top) or C19L1 (bottom) coupled to quantitative mass spectrometry. Proteins implicated in pre-mRNA splicing are labelled and bait proteins are underlined. The differences between groups were statistically evaluated using limma with a moderated parametric two-sided *t*-test. The multiple testing correction used the Benjamini–Hochberg procedure. The horizontal dashed line marks the significance threshold of *P* = 0.05, and the vertical dashed lines mark an enrichment threshold of log_2_ fold change of 0.75 over the TurboID-GFP control. Each experiment was carried out in biological triplicates.

**Extended Data Fig. 4:**
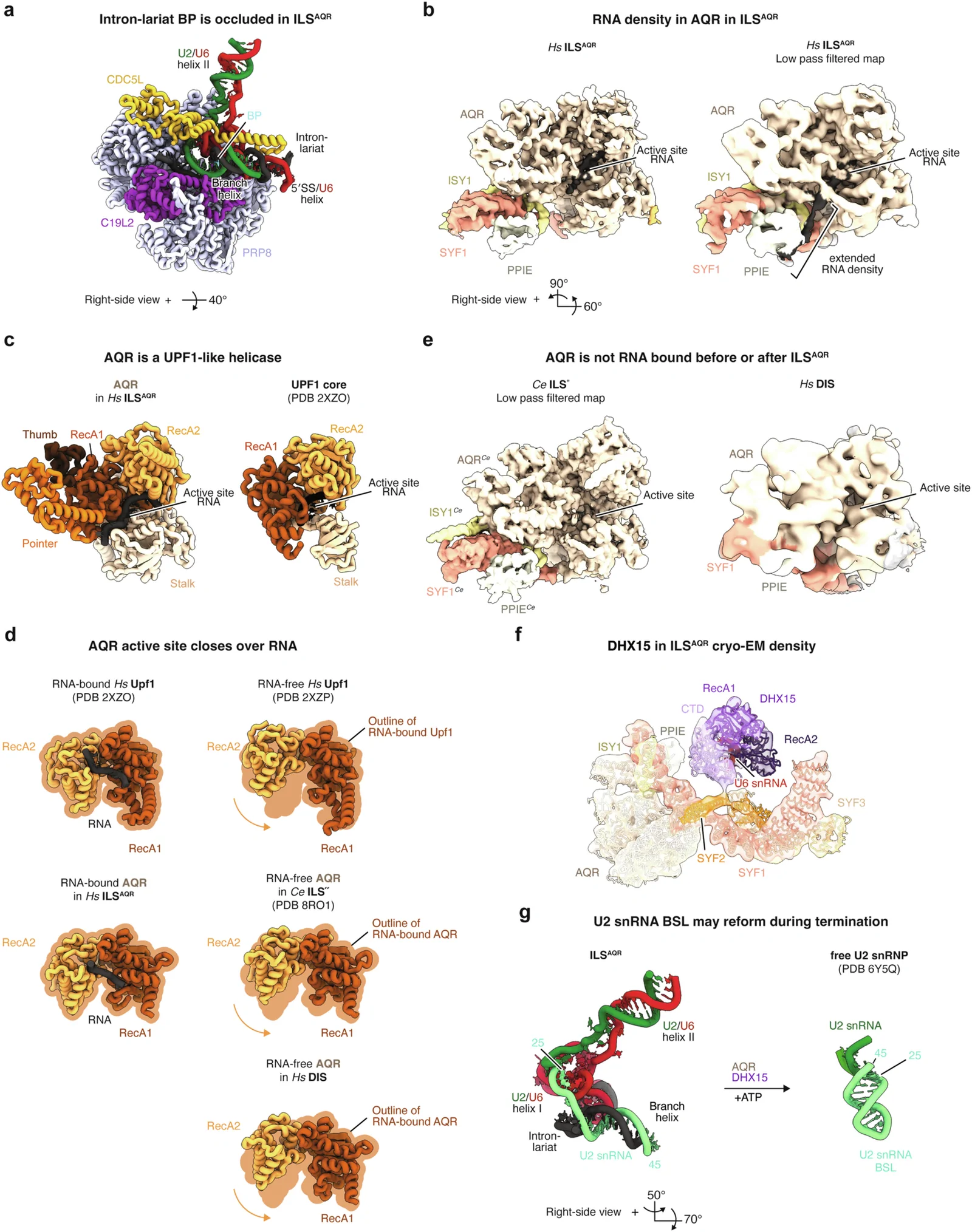
AQR and DHX15 extract the intron-lariat BP from the spliceosomal RNA network. **a**. Close-up view of the RNA active site of the ILS^AQR^ showing how the intron-lariat branch point (BP) is occluded from the solvent by both protein and RNA components of the spliceosome, making it inaccessible to debranching. **b**. Cryo-EM density of AQR and its surrounding proteins in the map of ILS^AQR^. The RNA density is observed in the active site of AQR, resolved at a nominal resolution of ~4 Å (left). Upon low pass filtering, further RNA density is observed extending from the AQR active site towards the RRM domain PPIE, which may guide the intron-lariat towards AQR (right). The density is coloured according to the protein and RNA it represents. **c**. Comparison of the RNA-bound AQR structure in the ILS^AQR^ (left) and the crystal structure of the RNA-bound UPF1 core (PDB 2XZ0; right)^30^. Protein domains of both helicases are indicated and coloured based on the scheme in Fig. 2a. **d**. Comparison of RNA-bound (top left) and RNA-free (top right) human Upf1 crystal structures^30^ with AQR in the human ILS^AQR^ (middle left), *C. elegans* ILS″ (middle right)^2^, and DIS (bottom) cryo-EM structures, showing that AQR adopts a closed RNA-bound conformation in the ILS^AQR^ but remains in an RNA-free open conformation in the ILS″ and DIS. The silhouette of the respective RNA-bound conformation of Upf1 and AQR is shown underneath the coordinate models of the different states. Arrows depict the direction of movement of the RecA2 domain upon RNA binding. **e**. Cryo-EM densities of AQR and its surrounding proteins in the ILS″ from *C. elegans* (EMD-19399)^2^ and in the human DIS, showing that RNA density is absent from the AQR active site in both complexes. The map of *Ce* ILS″ was gaussian filtered to match the density detail of the AQR density in the human ILS^AQR^. Despite the low local resolution of AQR in the DIS, its active site is empty. After intron-lariat BP extraction and debranching on the ILS^AQR^, AQR would likely run off the linearized intron RNA owing to AQR’s 5′-to-3′ polarity. This would leave AQR unable to further contribute to RNA remodelling, consistent with the absence of RNA density in the AQR active site in the DIS. **f**. Cryo-EM density of DHX15 after extensive image classification, revealed DHX15 relative to NTR components of the ILS^AQR^ at a nominal resolution of 9.5 Å from 9,659 particles (see Extended Data Fig. 2 for classification tree). The density is consistent with a model of DHX15 in complex with U6 snRNA and TFIP11 G-patch, as also seen in other ILS structures^2^. Both the model and the map are colored according to the protein they represent. The DHX15 domains were defined in ref. ^43^. **g**. Comparison of U2 snRNA conformation in the ILS^AQR^ (right) and in the free U2 snRNP (left; PDB 6Y5Q)^33^. The region of U2 snRNA corresponding to nucleotides 25-45 (light green) is base-paired with the intron-lariat forming U2/U6 helix I and the branch helix in the ILS^AQR^. The same region forms an internal branch point stem loop (BSL) in the free U2 snRNP. Remodelling of the ILS^AQR^ RNA network by the RNA helicases DHX15 and AQR, and specifically the dissolution of the branch helix by AQR, may thus allow the BSL to reform. This could further promote U2 snRNP release from the spliceosome.

The ILS^AQR^ structure substantially improves cryo-EM density quality of both core and peripheral spliceosome regions compared with previous human ILS structures^2,3^. Its unique features indicate that the ILS^AQR^ forms after the previously resolved ILS complexes (Supplementary Text 1). In the ILS^AQR^, we resolved the newly identified termination factor YJU2B (Fig. 2a) alongside the known termination factors TFIP11, GCFC2 (paralogue of PAXBP1; Supplementary Text 2), C19L1, C19L2 and DHX15 (Figs. 1c and 2). The ILS^AQR^ RNA active site contained the intron–lariat with the BP adenosine sequestered within the U2 snRNA–intron branch helix and enclosed by the U6 snRNA–5′ SS helix and C19L2 (Fig. 2b and Extended Data Fig. 4a). The ILS^AQR^ is thus committed to disassembly, but the disassembly process has not yet begun^2^.

Of note, we observed unambiguous RNA density in the active site of the RNA helicase AQR, locally resolved to 3.9 Å resolution (Fig. 2a–c and Extended Data Fig. 4b). On the basis of reports of AQR binding approximately 40 nucleotides on either side of the intron BP^14,28,29^, a proposed low-resolution RNA path^3^ and the relative positions of the RNA elements in the ILS^AQR^, we assigned this RNA as the circular portion of the intron–lariat. The intron–lariat is enclosed in an RNA-binding channel of AQR formed by its RecA1, RecA2 and Stalk domains, resembling the RNA-binding mode of the structurally related RNA helicase Upf1 (ref. ^30^) (Fig. 2a–c and Extended Data Fig. 4c). Unlike RNA-free AQR in the preceding ILS complexes, in the ILS^AQR^, the AQR RecA2 domain shifts position to close over the bound intron–lariat RNA (Extended Data Fig. 4d,e). The PPIE RRM domain sits at the entry of the AQR active site, suggesting that it guides the intron–lariat towards AQR (Fig. 2a–c and Extended Data Fig. 4b). AQR is an Upf1-like SF1 family helicase with single-stranded nucleotide translocase activity of 5′-to-3′ polarity^26,31^. Our structure suggests that AQR translocation along the intron–lariat would be poised to unwind the U2–intron branch helix and pull on the intron–lariat BP adenosine (Fig. 2c,d). This is reminiscent of the role of AQR in spliceosome activation, where it is thought to pull the intron to extract the branch helix from the SF3B complex, allowing the BP to participate in branching^26,27^. AQR could therefore assist both spliceosome activation and disassembly, as previously proposed^32^. Because the termination factor C19L2 occludes the buried intron–lariat BP in the ILS^AQR^, AQR translocation may also dissociate C19L2 from the spliceosome (Fig. 2b). Overall, our ILS^AQR^ structure supports a role for AQR in extracting the intron–lariat BP from the spliceosomal RNA network during termination.

In the ILS^AQR^, we also observed the RNA helicase DHX15, bound in the same position as in previous ILS structures^2–5^ (Fig. 2b and Extended Data Fig. 4f). DHX15 is a DEAH-box RNA helicase that unwinds the U2–U6 helix II in the ILS by translocating on U6 snRNA with 3′-to-5′ polarity^2,13^. Our structural data suggest that DHX15 and AQR may both act during spliceosome disassembly, either sequentially or simultaneously, to unwind different RNA elements: U2–U6 helices II and I, and the U2–intron branch helix, respectively (Fig. 2d). We hypothesize that this remodelling may further allow refolding of the internal U2 snRNA branch stem loop^33^, promoting U2 snRNA dissociation from the spliceosome (Extended Data Fig. 4g). Overall, these data suggest a model in which two RNAs pulled in opposite directions initiate dismantling of the RNA network of the spliceosome and extraction of the intron–lariat BP during ILS^AQR^ remodelling. The dependence on AQR probably varies across introns^14^, and we do not exclude AQR-independent routes to BP exposure for debranching.

## Recruiting the debranching enzyme to the spliceosome

Debranching of the intron–lariat is critical for intron turnover and nuclear RNA homeostasis^6,12^ and has been proposed to occur on the spliceosome^14–16^, although molecular evidence has been lacking. The DIS complex contains a debranched intron and forms after the ILS^AQR^ (see the section ‘ILS^AQR^ is remodelled into the DIS’), suggesting that debranching precedes intron ejection. In the ILS^AQR^, AQR is poised to extract the intron–lariat BP from the RNA active site, exposing it for debranching. This would, however, require the debranching enzyme DBR1 to be tethered near the extracted BP (Fig. 2e).

**Extended Data Fig. 5:**
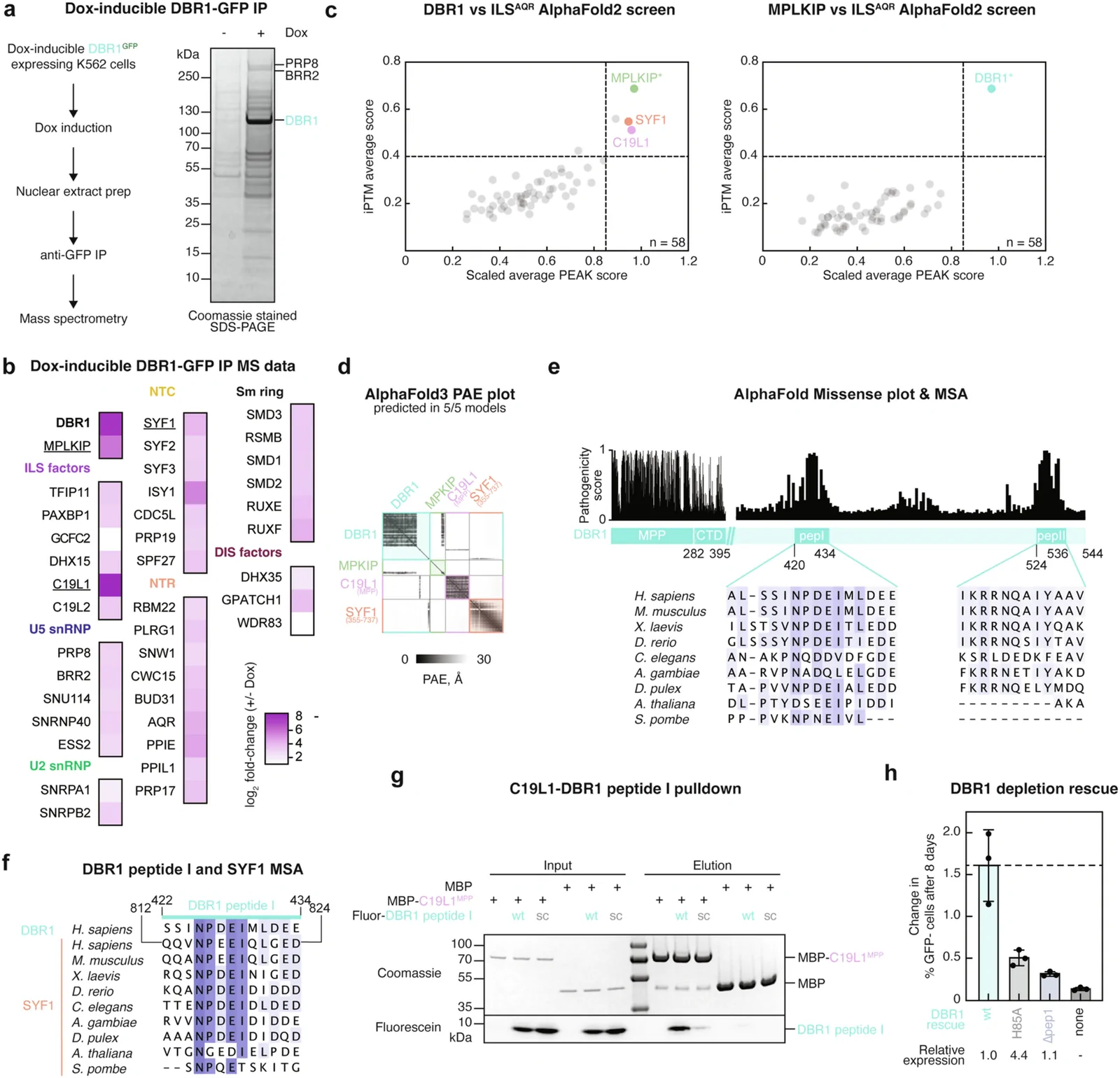
Characterization of DBR1 interactions with the spliceosome. **a**. Immunoprecipitation (IP) experiment of doxycycline (Dox) inducible GFP-tagged DBR1 from the nuclear extract of human K562 cells. Left: Experiment schematic. Right: SDS-PAGE analyses of the anti-GFP nanobody pulldowns from the nuclear extract of either uninduced (−; left lane) or doxycycline-induced (+; right lane) DBR1-GFP K562 cells. The pulldown was performed five times. The molecular weight in kilodalton (kDa) of the molecular weight marker is indicated to the left of the gel. The band corresponding to GFP-tagged DBR1 and the bands corresponding to spliceosome components PRP8 and BRR2 are labelled to the right of the gel, indicating the purification a DBR1-containing spliceosome complex. **b**. Heatmap of the quantitative mass spectrometry data of the DBR1-GFP immunoprecipitation experiments described in panel a. The data were normalized for protein abundance in the pulldown from the uninduced cells. The heatmap shows specific enrichment in the DBR1-GFP pulldown of the known DBR1 interactor MPLKIP and of the ILS components, especially the disassembly factor C19L1. The experiment was performed in technical triplicates. **c**. Pairwise AlphaFold2 Multimer^36,37^ protein interaction screens of DBR1 (left) and MPLKIP (right) against spliceosome factors identified by mass spectrometry of the ILS^AQR^ complex. Proteins with both a high iPTM score^37^ and a high PEAK score^78^ were selected as putative interactors. DBR1 and MPLKIP were additionally included in each other’s screens as respective positive controls. Although MPLKIP was previously proposed to recruit DBR1 to spliceosomes^34^, we did not find confident predictions for a binding interface between MPLKIP and any of the ILS^AQR^ proteins. The predicted interfaces of DBR1 with MPLKIP, SYF1, and C19L1 are fully compatible with each other and could occur at the same time. **d**. Predicted alignment error (PAE) plot of the DBR1–MPLKIP–C19L1–SYF1 complex prediction from AlphaFold3. The plot shows that DBR1 could bind to all three interactors simultaneously. Each binary interaction was confidently predicted in five out of five models. **e**. DBR1 interacts with the spliceosome via two conserved peptides. Top: Average predicted pathogenicity score per residue of DBR1 according to AlphaFold Missense^79^. Mutations in the catalytic MPP domain of DBR1 appear to be universally pathogenic, while the disordered C-terminal region of DBR1 contains two stand-out regions of increased pathogenicity, corresponding to peptides I and II. Bottom: Multiple sequence alignment (MSA) of DBR1 peptides I and II. Peptide I displays a higher degree of sequence conservation across eukaryotes than peptide II, suggesting that peptide I might be more functionally important. Peptide II may overlap with the nuclear localization sequence of DBR1 (ref. ^80^). Each residue was coloured according to its BLOSUM62 conservation score. **f**. Multiple sequence alignment (MSA) of the human DBR1 peptide I with a highly similar peptide in the spliceosomal protein SYF1 from various eukaryotic species. This SYF1 peptide may also bind the C19L1 MPP domain, suggesting that DBR1 peptide I may displace SYF1 to associate with the spliceosome, potentially explaining why DBR1 associates transiently with ILS complexes at steady state. The SYF1 peptide, which is predicted to bind C19L1, lies adjacent to an I-patch motif that may inhibit the nearby DHX15 (ref. ^42^) (Fig. 2b). Displacement of SYF1 from C19L1 by DBR1 peptide I could release this inhibitory motif, promoting DHX15 activation and physically coupling ILS remodelling to DBR1 binding. The biological significance of these observations remains to be determined. Each residue in the MSA was coloured according to its BLOSUM62 conservation score. **g**. In vitro pulldown experiment with purified recombinant proteins demonstrating a direct and specific interaction between DBR1 peptide I and the MPP domain of C19L1. MBP-tagged C19L1 MPP (residues 1-278) was immobilized on amylose beads and incubated with either wild-type (wt) fluorescein-tagged DBR1 peptide I or a scrambled (sc) version of the same peptide. Only the wild-type peptide showed an interaction with C19L1 MPP. Amylose beads with immobilized MBP were used as a control to assess the background binding of the DBR1 peptides. The pulldown was performed three times. The molecular weight in kilodalton (kDa) of the molecular weight marker is indicated to the left of the gels. **h**. Depletion-rescue experiments show that the DBR1–C19L1 interaction via DBR1 peptide I is needed for normal cell growth. DBR1-dTAG HEK293T human cells were mixed at a 1:1 ratio with GFP-expressing HEK293T cells. The small molecule dTAG^V^-1 was added to the culture to deplete endogenous DBR1 only in the GFP negative cells. Expression of various DBR1 rescue constructs was simultaneously induced by adding doxycycline. The proportion of GFP negative (DBR1-depleted) cells was assessed by fluorescence-activated cell sorting after eight days. Wild-type DBR1 (wt) overexpression was significantly more effective at rescuing cell growth than overexpression of either the catalytic mutant DBR1 (H85A) or DBR1 lacking peptide I (Δpep1). This indicates that peptide I contributes to normal DBR1 function in cells. The fluorescence signal of the mScarlet-tagged DBR1 constructs was measured by FACS one day after the start of doxycycline induction. The mScarlet levels relative to wild-type DBR1 are indicated underneath the plot. The wild-type DBR1 was expressed at the lowest level out of the three constructs, demonstrating that measured growth defects of the two mutants are not explained by low expression levels. Average values from three biological replicates measured in technical triplicates are plotted, and the standard deviation is indicated.

DBR1 was absent from the ILS^AQR^ mass spectrometry data, probably reflecting its transient interaction with the spliceosome. To identify candidate spliceosomal interactors of DBR1, we immunoprecipitated overexpressed GFP-tagged DBR1 from human K562 cells (Extended Data Fig. 5a). This enriched the known DBR1 interactor MPLKIP^34^ and ILS-specific termination factors, consistent with previous results^12,13^ (Extended Data Fig. 5b and Supplementary Table 2). Moreover, proximity labelling of DBR1 fused to the biotin ligase TurboID^35^ enriched MPLKIP, the termination factor C19L1 and the NTR complex (Fig. 2f and Supplementary Table 4). A reciprocal experiment using C19L1–TurboID enriched DBR1 and MPLKIP, supporting their proximity in spliceosomal complexes (Fig. 2f and Supplementary Table 4).

To investigate the molecular basis of DBR1 binding to the spliceosome, we performed AlphaFold2 Multimer^36,37^ screens of DBR1 and MPLKIP against the proteins present in the ILS^AQR^ (Extended Data Fig. 5c). We obtained high-confidence predictions only for DBR1 with the termination factor C19L1 and the NTR protein SYF1, involving two conserved peptides in the DBR1 C terminus (Fig. 2a and Extended Data Fig. 5d,e). Peptide I (DBR1 residues 420–434) was predicted to bind to the metallophosphatase (MPP) domain of C19L1, and peptide II (residues 524–532) to the helical repeats of SYF1 (Fig. 2e and Extended Data Fig. 5d). These predictions agree with our DBR1 proximity labelling data (Fig. 2f) and previous biochemical data^15,16^. Both peptides could simultaneously engage with the ILS^AQR^ and would be compatible with the predicted DBR1–MPLKIP interface (Fig. 2e). Peptide I is particularly well conserved and would tether the catalytic domain of DBR1 to the C19L1 MPP domain through a 30-residue linker, placing DBR1 near the intron–lariat BP after its extraction (Fig. 2e). The NTR subunit SYF1 carries a similar motif (SYF1 residues 812–824), which may indicate exchange between the two peptides at C19L1 during termination (Extended Data Fig. 5f).

We confirmed the predicted DBR1–C19L1 interaction with recombinant proteins, where wild-type DBR1 peptide I, but not its scrambled version, bound the C19L1 MPP domain in vitro (Extended Data Fig. 5g). Furthermore, we have shown that overexpression of wild-type DBR1 rescued the cell growth defect caused by acute depletion of endogenous DBR1–dTAG^38^, whereas DBR1 lacking peptide I did not (Extended Data Fig. 5h), demonstrating the cellular importance of the DBR1–C19L1 interface.

Together, we established a direct DBR1–ILS interaction that may facilitate intron–lariat debranching on the spliceosome after DHX15 and AQR extract the intron–lariat BP from the ILS RNA network.

## YJU2B may guide ILS^AQR^ remodelling

**Extended Data Fig. 6:**
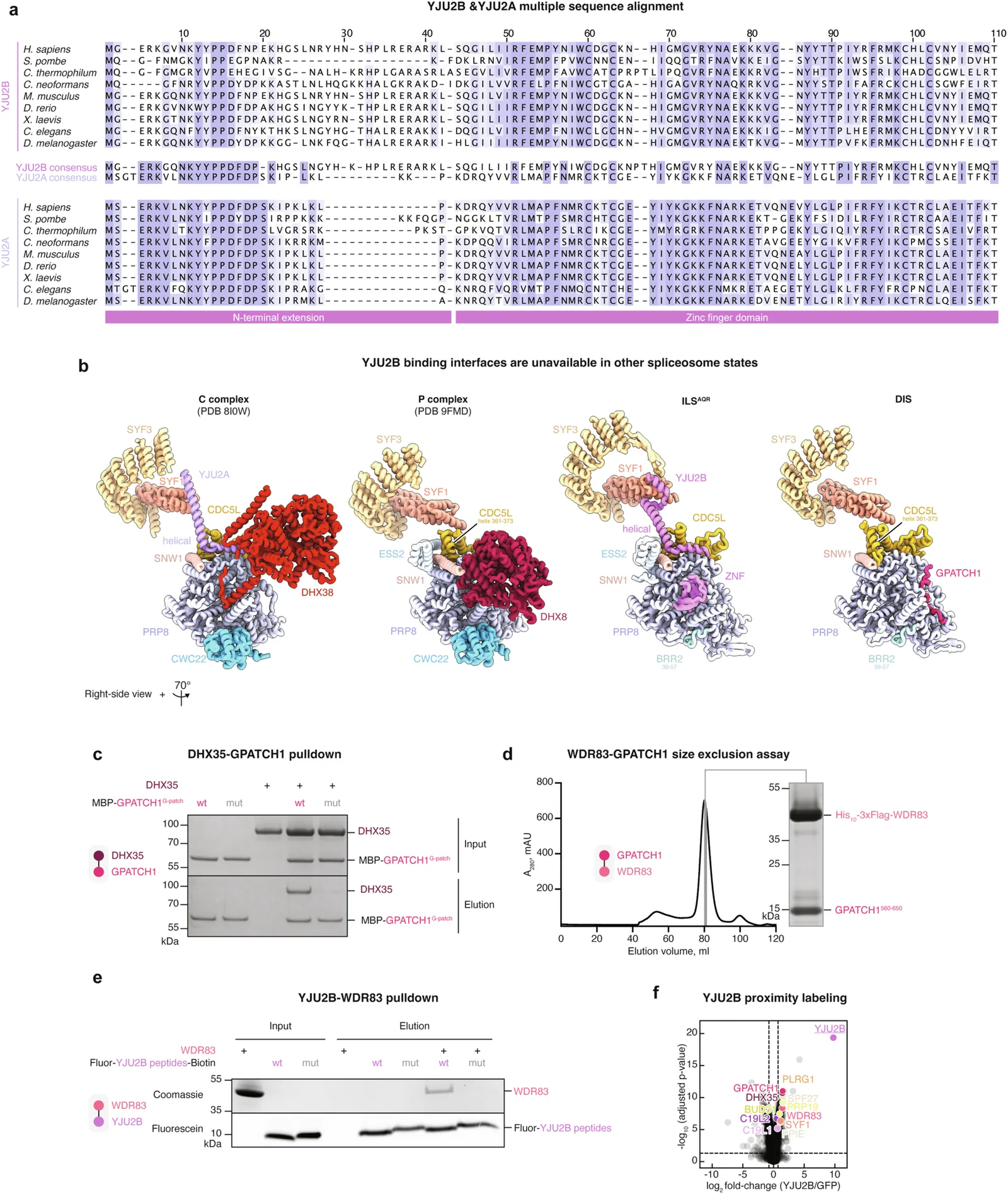
YJU2B conservation and characterization of YJU2B–DHX35–GPATCH1–WDR83 interactions. **a**. Multiple sequence alignment (MSA) of the N-terminal extension and the zinc finger domain of YJU2B and YJU2A. A comparison of the consensus sequences of YJU2A and YJU2B is also shown in the middle. Both proteins are highly conserved across eukaryotes, and their shared sequence features indicate that YJU2A and YJU2B are paralogues. Residues are coloured according to their BLOSUM62 conservation scores. **b**. Comparison of the YJU2B binding site in the ILS^AQR^ with the equivalent regions in other spliceosome states^2,56^, where the YJU2B binding interfaces are occupied by other state-specific factors. The binding site of YJU2B in the ILS^AQR^ is similar to how YJU2A binds to the C complex spliceosome with a few notable exceptions. In the ILS^AQR^, YJU2B additionally interacts with the spliceosome subunit ESS2, which is absent in the C complex. YJU2B also has a more extensive interaction interface with SYF1 compared to YJU2A. Finally, the zinc finger (ZNF) domain of YJU2A stabilizes the RNA active site in the C complex, while the same domain of YJUB occupies a surface on PRP8 in the ILS^AQR^. **c**. In vitro pulldown experiment with purified recombinant proteins demonstrating a direct and specific interaction between DHX35 and the G-patch domain of GPATCH1 (residues 137-247). Either wild-type (wt) or mutant (mut; M163K, V171R, L236E) MBP-tagged G-patch domain of GPATCH1 was immobilized on amylose beads and incubated with DHX35. DHX35 only bound to the wild-type but not to the mutant GPATCH1 G-patch domain. The pulldown was performed two times. The molecular weight in kilodalton (kDa) of the molecular weight marker is indicated to the left of the gel. A diagram summarizing the demonstrated interaction is shown on the left. **d**. Size exclusion chromatography of the purified recombinant WDR83-GPATCH1 complex. An SDS-PAGE analysis of the peak fraction is shown on the right with the molecular weight in kilodalton (kDa) of the molecular weight marker indicated to the left of the gel. His_10_−3xFlag-tagged WDR83 was co-expressed with its predicted interacting region of GPATCH1 (residues 560-650) in Hi5 insect cells. The complex was purified by nickel affinity and ion exchange chromatography before the run on a Superdex 200 16/60 gel filtration column. The complex was successfully co-expressed and co-purified demonstrating a stable interaction between WDR83 and GPATCH1. The size exclusion run was performed once during the purification of the complex. A diagram summarizing the demonstrated interaction is shown on the left. **e**. In vitro pulldown experiment with purified recombinant proteins demonstrating a direct and specific interaction between WDR83 and YJU2B. Fluorescein-labeled and biotin-tagged YJU2B peptides (residues 134-178) of either wild-type (wt) sequence or containing mutations at the predicted interface with WDR83 (mut; M146A L149K E150K) were immobilized on StrepTactin beads and incubated with WDR83. WDR83 bound to the beads only in the presence of the wild-type but not mutant YJU2B peptide. The pulldown was performed four times. The molecular weight in kilodalton (kDa) of the molecular weight marker is indicated to the left of the gel. A diagram summarizing the demonstrated interaction is shown on the left. **f**. Proximity labelling using TurboID fused to YJU2B coupled to quantitative mass spectrometry in human cells. Enriched proteins implicated in pre-mRNA splicing are labeled, and the bait protein YJU2B is underlined. Differences between groups were statistically evaluated using limma with a moderated two-sided t-test. The multiple testing correction used the Benjamini-Hochberg procedure. The horizontal dashed line marks the significance threshold of p = 0.05, the vertical dashed line indicates an enrichment threshold of log_2_-fold change of 0.75 (68% enrichment on a linear scale) over the TurboID-tagged GFP control. Each experiment was carried out in biological triplicates.

The remodelling of ILS^AQR^ by DHX15 and AQR would be expected to rearrange the RNA network for debranching, dissociating the ‘early’ termination factors C19L1, C19L2, GCFC2, TFIP11 and DHX15, and exposing surfaces for binding of the ‘late’ termination factors DHX35, GPATCH1 and WDR83 (Fig. 1c and Extended Data Fig. 1a). The essential and highly conserved protein YJU2B (Extended Data Fig. 6a) is ideally positioned to guide this exchange. In the ILS^AQR^, the YJU2B zinc-finger domain binds the PRP8 linker and finger-palm domains, otherwise inaccessible in catalytic and post-catalytic spliceosomes (Fig. 2e and Extended Data Fig. 6b). The YJU2B helical domain displaces a CDC5L α-helix (residues 357–382) from the ESS2–SNW1–PRP8 interface in the preceding ILS″ (Fig. 2e and Extended Data Fig. 6b), and runs along SYF1 and SYF3 to help position the C19L1 MPP domain and thereby DBR1 (Fig. 2e). Although WDR83, GPATCH1 and DHX35 were not detected in the final purified ILS^AQR^ sample, low levels were present before gradient purification, indicating transient association (Extended Data Fig. 1g). Consistently, using purified proteins in in vitro pulldowns and gel filtration assays, we confirmed the interactions predicted by AlphaFold3 between YJU2B–WDR83, WDR83–GPATCH1 and GPATCH1–DHX35 (Fig. 1b and Extended Data Figs. 1b,c and 6c–e). DHX35 and GPATCH1 were also enriched in proximity labelling experiments using both YJU2B and C19L1 as bait (Fig. 2f, Extended Data Fig. 6f and Supplementary Table 4). We therefore propose that YJU2B may aid the recruitment of DHX35, GPATCH1 and WDR83 during ILS^AQR^ remodelling.

## ILS^AQR^ is remodelled into the DIS

**Extended Data Fig. 7:**
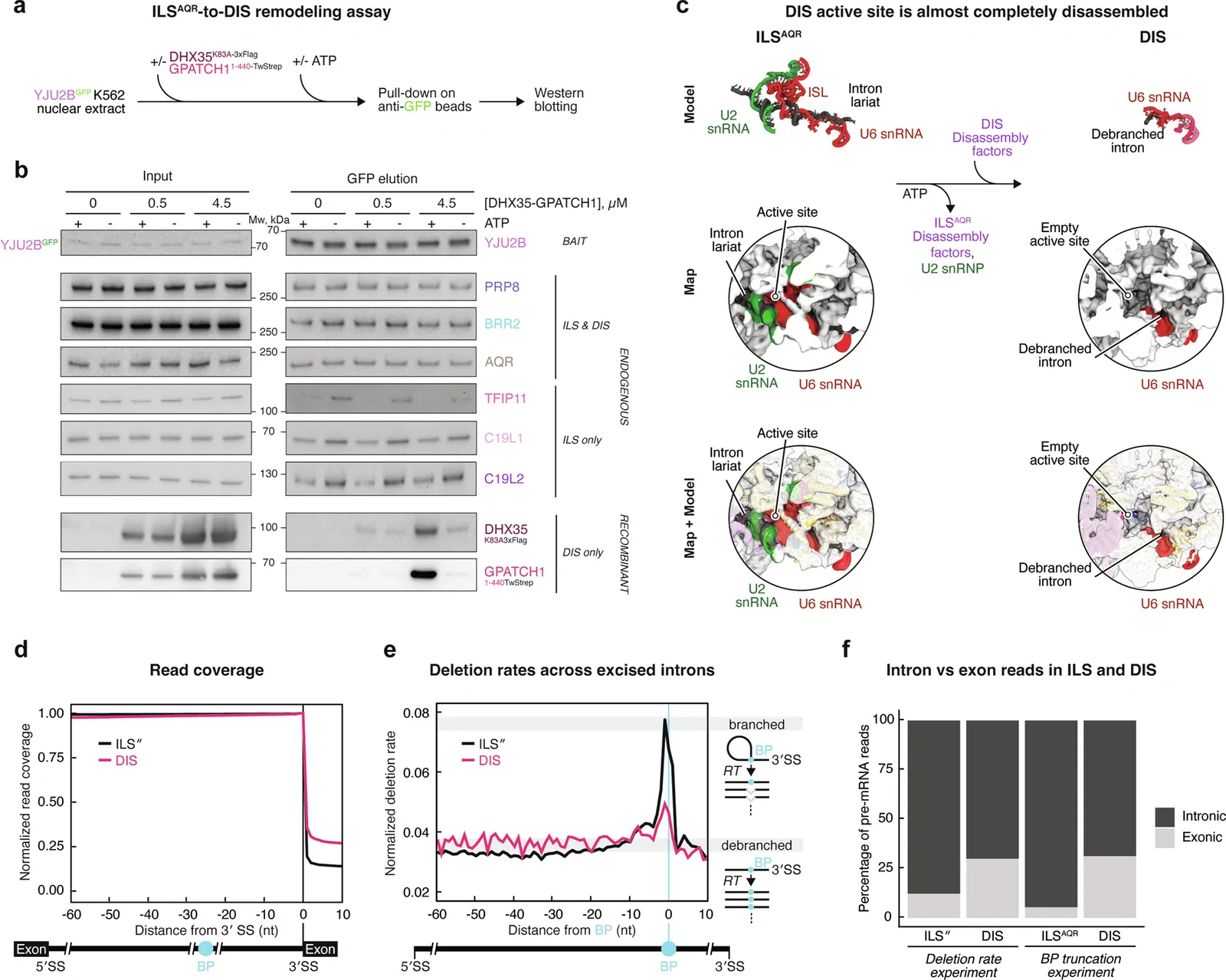
ILS remodelling and intron-lariat debranching precede DIS formation. **a**. Experimental scheme of the in vitro ILS^AQR^-to-DIS remodelling assay. **b**. Western blot analyses of the ILS^AQR^-to-DIS remodelling assay. The proteins in the treated extract before the pulldown (Input) are shown on the left, while the proteins bound to the anti-GFP beads (GFP elution) are shown on the right. The proteins were eluted by 3C protease cleavage of the GFP tag from YJU2B. The DHX35^K83A^-GPATCH1^1−440^ construct included the minimal region of GPATCH1 required to bind both DHX35 and the spliceosome but lacked the region binding WDR83 (GPATCH1 residues 574-654) to prevent alternative means of recruitment to the spliceosome, for example, through the YJU2B-WDR83 interaction. DHX35 and GPATCH1 were detected using anti-Flag and anti-Strep antibodies, respectively. The experiment was performed two times. **c**. Comparison of models (top), cryo-EM density maps (middle) and models of proteins and RNAs fit into the corresponding maps (bottom) of the RNA-based active site of the ILS^AQR^ (left) and DIS (right) complexes. The cryo-EM densities of both complexes were low pass filtered and clipped to highlight each RNA active site. The cryo-EM densities corresponding to RNA elements are coloured according to RNA identity, while the rest of the spliceosome is coloured in grey. Proteins in the bottom panels are coloured by identity in cartoon representation. The cavity of the active site is empty in the cryo-EM density of the DIS, demonstrating that the RNA network of the DIS has been almost completely dismantled, excepting the partial U6-5′SS duplex and the U6 5′-end. **d**. Normalized read coverage at pre-mRNA intron 3′-ends, contained within ILS″ or DIS complexes, representing 533,004 reads in the ILS″ and 16,207 reads in the DIS. Read coverage at the annotated 3′SS was set to 1. The nucleotide distance from the 3′SS is indicated on the x-axis and explained with a pre-mRNA schematic underneath. **e**. Normalized deletion rates of excised introns contained within DIS or ILS″ complexes, aligned at the branch point adenosine, representing 452,677 reads in the ILS″ ^AQR^and 13,224 reads in the DIS. Deletions are caused by error-prone reverse transcription through a branched intron-lariat, illustrated in the diagram on the right. Intron-lariats of the purified ILS″ produced a sharp peak in deletion rate around the annotated BP as expected. In contrast, introns of the purified DIS displayed a more uniform deletion rate with a significantly reduced peak at the annotated BP, providing additional evidence that DIS-associated introns are predominantly debranched. Owing to the low frequency of reverse transcription errors, the observed deletion rate significantly underestimates the absolute percentage of branched introns. Deletion rates are calculated per position and normalized to the deletion rate across all read positions. The nucleotide distance from the branch point is indicated on the x-axis and explained with an intron schematic underneath. **f**. Bar chart showing the proportion of intronic and exonic reads among the pre-mRNA reads detected in the ILS (ILS″ in the deletion rate experiment or ILS^AQR^ in the BP truncation experiment) and DIS complexes in the branch-point deletion rate experiment depicted in panels d and e (left) and in the branch point truncation experiment shown in Fig. 3 (right) (see Materials and Methods for details). The majority of pre-mRNA reads in both samples in both experiments come from introns, which, together with the intronic read coverage plots in panel d and in Fig. 3h, suggests that both the ILS and the DIS are associated primarily with excised introns. The higher percentage of exonic reads in the DIS sample compared to the ILS may indicate either contamination or that some defective catalytic spliceosomes were also purified in the DHX35-GFP pulldown in addition to the DIS complex, consistent with the role of DHX35 in spliceosome quality control^5,23^. We cannot exclude that a fraction of particles in our DIS cryo-EM reconstruction originates from SQC complexes that assembled prior to pre-mRNA branching or after branching and debranching. However, we consider this unlikely owing to (i) the lack of cryo-EM density for the 5′-exon in the exon channel, (ii) the absence of LENG1 and CWC22, which would be expected in a B*^D^ SQC complex, and (iii) the predominant association of excised and debranched introns with the DIS in the two RNA sequencing experiments.

To probe the ILS^AQR^-to-DIS transition in the human system, we used an in vitro remodelling assay using human nuclear extract expressing GFP-tagged YJU2B (Extended Data Fig. 7a). ATP treatment caused the depletion of the ILS-specific termination factors (TFIP11, C19L1 and C19L2) from anti-GFP-purified spliceosomes (Extended Data Fig. 7b). Given the low abundance of endogenous DIS factors, we supplemented the assay with a recombinant heterodimer of catalytically inactive DHX35(K83A) and a minimal GPATCH1 construct (residues 1–440; Extended Data Fig. 7b). DHX35(K83A)–GPATCH1 was recruited to the remodelled ILS^AQR^ in an ATP-dependent manner, consistent with the formation of a DIS-like intermediate (Extended Data Fig. 7b) and our *C. elegans* assay (Extended Data Fig. 1a). These results support a model in which ILS^AQR^ is remodelled into DIS, possibly involving additional short-lived intermediates.

**Fig. 3:**
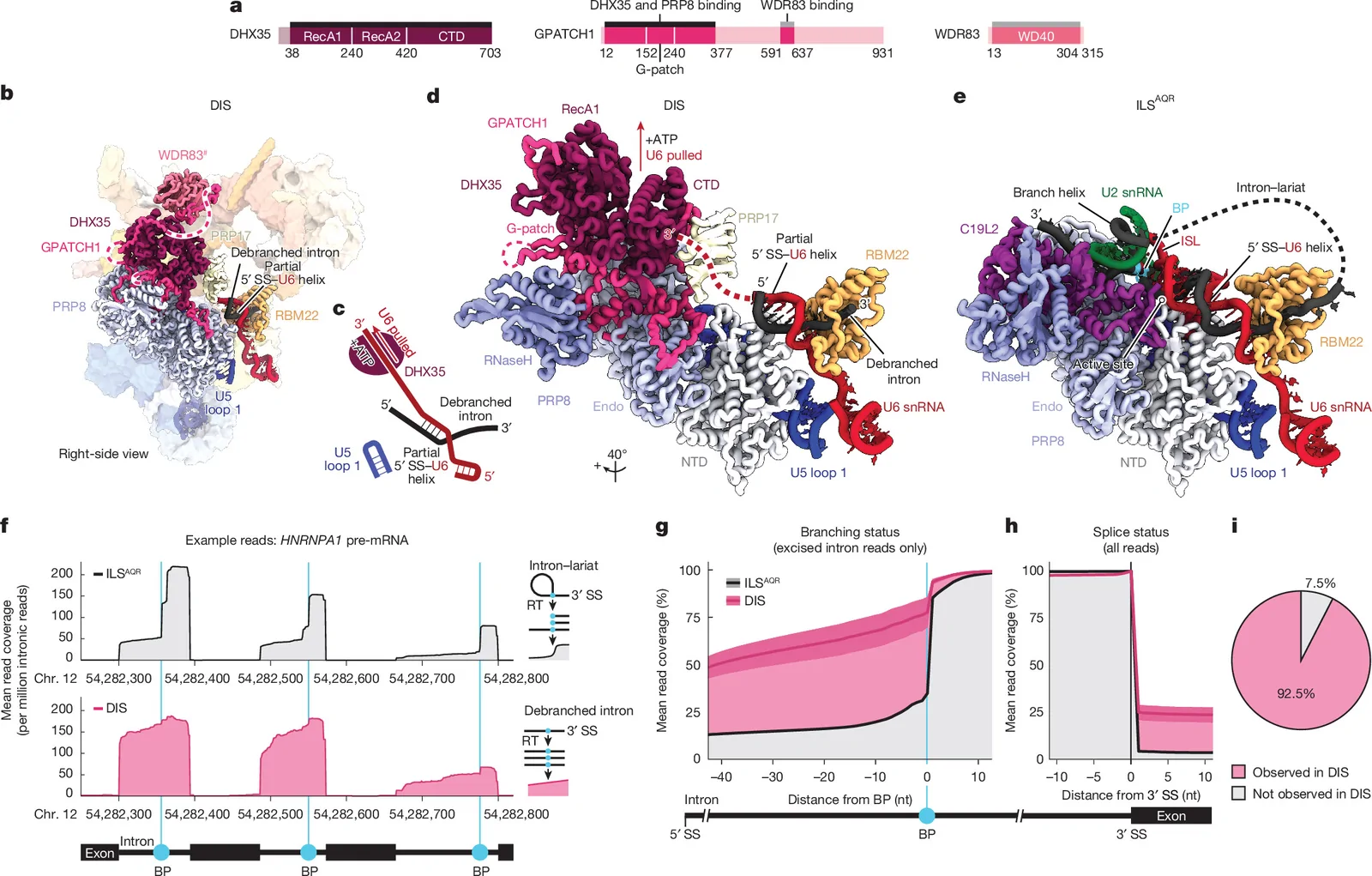
Features of the DIS. **a**, Domain organization of DHX35, GPATCH1 and WDR83. The regions modelled in the DIS are marked with black lines. **b**, DIS structure with termination factors (DHX35, GPATCH1 and WDR83), their interactors and RNA elements highlighted. The remainder of the complex is shown as a transparent surface. The orientation of WDR83 is not assigned (marked with #) due to the low local resolution. **c**, Schematic of the DIS RNA active site, with the arrow indicating the DHX35 pulling direction. **d**, Interactions of DHX35 and GPATCH1 with the DIS and a zoomed-in view of the DIS RNA network. The putative path of the U6 snRNA 3′ region towards DHX35 is indicated with a dashed line. **e**, The same region of the ILS^AQR^ as shown for the DIS in **d**. ISL, internal stem loop. **f**, Mean read coverage of adjacent introns of the *HNRNPA1* transcript contained within ILS^AQR^ or DIS complexes across three technical replicates. Both ILS^AQR^ and DIS samples were subsampled to 100 reads each. The expected patterns of read coverage of branched intron–lariats and debranched introns are schematically depicted (right). RT, reverse transcription. **g**, Normalized mean coverage of reads mapping to intron 3′ ends (excised introns) contained within ILS^AQR^ or DIS complexes aligned at the predominant BP. A total of 89,807 introns were included, representing 17,543,102 reads in ILS^AQR^ and 479,338 reads in DIS across three technical replicates. The shaded regions indicate 95% confidence intervals. nt, nucleotide. **h**, Normalized mean coverage at pre-mRNA intron 3′ ends of all reads, contained within ILS^AQR^ or DIS complexes across three technical replicates. The plot includes 14,042,209 reads in ILS^AQR^ and 338,613 reads in DIS. The shaded regions indicate 95% confidence intervals. **i**, Pie chart showing that 92.5% of the top half of most highly abundant ILS^AQR^ introns are also detected in DIS.

To understand the molecular consequences of ILS^AQR^ remodelling and the roles of DHX35–GPATCH1–WDR83 (Fig. 3a) in termination, we next analysed the DIS structure (Figs. 1c and 3b). Its protein and RNA composition resembled the 35S spliceosomal complex, which accumulates at the end of pre-mRNA splicing in vitro^18,19^. The DIS contained the NTR, the NineTeen complex (NTC), the U5 snRNP and the DIS-specific termination factors DHX35, GPATCH1 and WDR83 (Fig. 1c), but lacked the six ILS-specific termination factors (TFIP11, GCFC2, C19L1, C19L2, DHX15 and YJU2B), the U2 snRNP and ESS2 (Figs. 1c and 3d,e and Extended Data Fig. 1g). The DIS RNA network was almost completely dismantled, with only a partial U6–intron–5′ SS duplex remaining (Fig. 3c–e and Extended Data Fig. 7c). Remodelling of the ILS^AQR^ by AQR and DHX15 thus causes major compositional and conformational changes without complete disassembly of the spliceosome.

In the ILS^AQR^, AQR would extract the intron–lariat BP for debranching by the C19L1-tethered DBR1. As C19L1 is absent from the DIS, which forms after the ILS, we reasoned that introns bound to the DIS are probably debranched. As we observed no cryo-EM density for the intron BP in the DIS (Extended Data Fig. 7c), we used an RNA sequencing assay to determine the branching status of the DIS-bound introns. RNAs from purified ILS^AQR^ and DIS complexes showed high read coverage across introns that sharply dropped at the 3′ SS–3′ exon boundary, confirming that both complexes primarily contained excised introns (Fig. 3f–h). Because reverse transcription often terminates at the tripartite RNA junction around the BP of a branched intron–lariat^39^, branched introns can be identified by a drop in read coverage upstream of the BP relative to downstream, where reverse transcription initiates at the 3′ SS (Fig. 3f). Individual introns associated with the ILS^AQR^ showed a sharp decrease in read coverage upstream of the predominant BP, whereas the same introns bound to the DIS showed more uniform read coverage (Fig. 3f). We observed the same trend globally across all excised introns detected (Fig. 3g). The vast majority of DIS-associated introns are therefore debranched. We confirmed this using an orthogonal long-read RNA sequencing approach, analysing deletions introduced during reverse transcription around branched BPs^40,41^ (Extended Data Fig. 7d–f). A subset of DIS-associated introns showed signs of branching in both experiments (Fig. 3g and Extended Data Fig. 7e), suggesting that debranching largely occurs on the spliceosome but is not a prerequisite for the ILS^AQR^-to-DIS transition. These branched introns may represent complexes in which debranching is delayed or AQR independent.

To test whether the ILS^AQR^-to-DIS transition represents a general pathway for human introns, we analysed the overlap among the excised introns associated with each complex. If the ILS^AQR^ is remodelled into DIS, introns detected as branched lariats in the ILS^AQR^ should be detected as debranched, linear species in the DIS. Indeed, the vast majority of ILS^AQR^-associated introns were detected in the DIS sample (Fig. 3i), indicating that sequential passage through ILS^AQR^ and DIS is the predominant spliceosome termination pathway.

These combined biochemical and structural data support intron–lariat debranching on the spliceosome and reveal a new spliceosome state containing a debranched intron.

## Intron ejection from the DIS

**Extended Data Fig. 8:**
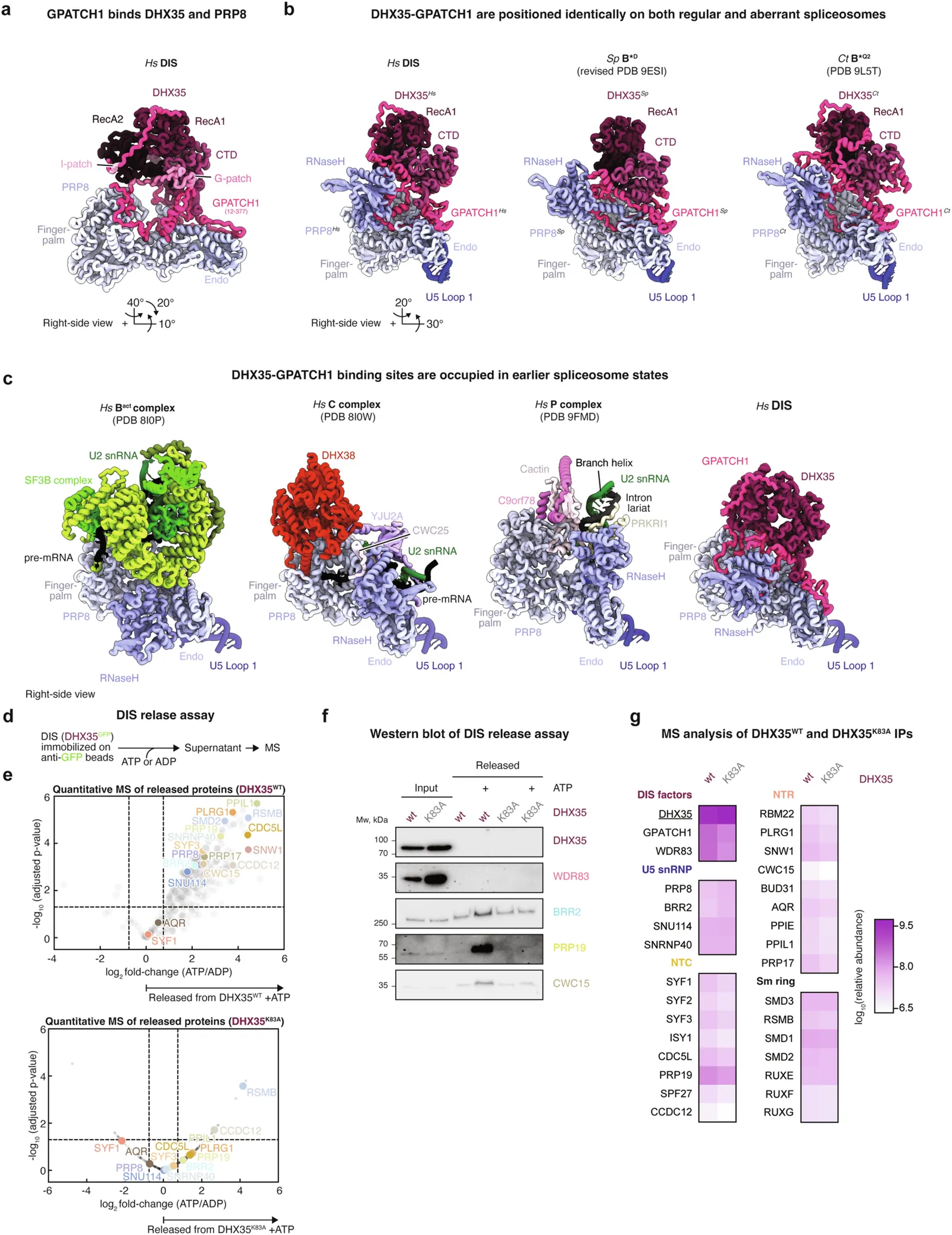
Termination factor interactions and release from the DIS. **a**. Close-up view of GPATCH1 and its interactions with PRP8 and DHX35 within the DIS. PRP8 and DHX35 are coloured according to their protein domains, respectively. The activating G-patch domain^43^ and the inhibitory I-patch domains^42^ of GPATCH1 are indicated. **b**. Comparison of the interactions between DHX35, GPATCH1 and PRP8 in the human DIS (left) and the defective B*^D^ spliceosomes from *S. pombe*^23^ (middle) and *C. thermophilum*^5^ (right). DHX35 and GPATCH1 bind through similar and conserved interfaces in both regular and defective spliceosomes. PRP8 and DHX35 are coloured by their domains with the domains of the *S. pombe* and *C. thermophilum* proteins assigned based on their structural similarity to the human orthologues. **c**. Comparison of the DHX35–GPATCH1 binding site in the DIS to the equivalent regions in earlier state spliceosomes. The DHX35–GPATCH1 binding surface on PRP8 is occupied by other spliceosomal factors in all earlier spliceosome states: the SF3B complex of the U2 snRNP in the B^act^ complex^56^, the RNA helicase DHX38 in the C complex^56^, and the state-specific factors Cactin and C9orf78 and the branch helix in the P complex^2^. **d**. Experimental scheme of the DIS release assay. **e**. DIS release assay coupled to mass spectrometry of DIS complexes purified on either wild-type (wt) DHX35 (top) or catalytic mutant (K83A) DHX35 (bottom), DIS-associated proteins are released upon ATP-treatment in the presence of wild-type but not mutant DHX35. DHX35, GPATCH1, WDR83 are not released and remain on the beads (Extended Data Fig. 8f). These data indicate that DHX35 could catalyse spliceosome disassembly. Differences between groups were statistically evaluated using limma with a moderated two-sided t-test. The multiple testing correction used the Benjamini-Hochberg procedure. The horizontal dashed line represents a significance threshold of p-value = 0.05, the vertical dashed line marks a log_2_-fold change threshold of one (two-fold change on a linear scale). **f**. Western blot analyses of the DIS release assay. One replicate of each sample from experiment in panel e was analysed by western blotting. BRR2, PRP19 and CWC15 were specifically probed and released from the bead-immobilized DIS complex in the presence of wild-type (wt) DHX35 upon addition of ATP, consistent with the quantitative mass spectrometry data. In contrast, DHX35 and GPATCH1 were probed and not released, consistent with neither protein being detected among the released proteins by mass spectrometry. This shows that the release of the spliceosomal proteins is not caused by non-specific dissociation of DHX35 from the beads. Two independent experiments were performed. **g**. Heatmap of the mass spectrometry data of the immuno-purified spliceosomes containing either wild-type (wt) DHX35-GFP or catalytic mutant (K83A) DHX35-GFP. DHX35^K83A^ contains a mutation in the highly conserved P-loop region^81^, abrogating ATP binding and RNA helicase activity. Both wild-type and catalytic mutant DHX35 co-purify essentially identical spliceosome complexes, which match the composition of the DIS. This shows the suitability of the DHX35^K83A^-containing DIS as a negative control in DIS release assays and strongly suggests that the DIS represents a spliceosome state before DHX35 RNA helicase action.

To disassemble and recycle the spliceosome^9,10^, the debranched intron must be ejected from the DIS, which may require the correct positioning and activation of the DIS-specific RNA helicase DHX35. In the DIS, DHX35 is anchored primarily through its contacts with GPATCH1 and the PRP17 WD40 domain (Fig. 3d and Extended Data Fig. 8a,b). GPATCH1 interacts with DHX35 through several regions, including its regulatory G-patch and I-patch domains that bind across the RecA1 domain and the C-terminal domain of DHX35 (Extended Data Fig. 8a) similar to how other co-factors interact with their cognate DEAH-box RNA helicases^42,43^. The DHX35-interacting segments of GPATCH1 are interspersed by PRP8-binding regions that wind around the endonuclease, RNaseH and finger-palm domains (Fig. 3d and Extended Data Fig. 8a,b). A C-terminal GPATCH1 motif tethers WDR83 (Fig. 3a,b). Of note, the surfaces that bind to DHX35 and GPATCH1 are available in the DIS but occluded in earlier on-pathway spliceosome states, which could prevent premature termination (Extended Data Fig. 8c). Moreover, DHX35–GPATCH1 binding to PRP8 in the DIS would clash with C19L2, the RNA active site and the intron–lariat BP in the ILS^AQR^ (Fig. 3d,e), rationalizing why ILS^AQR^ remodelling is required to form the DIS with correctly positioned DHX35.

To understand how DHX35 may act in the DIS, we examined its RNA network. In the preceding states (from pre-B^act^ to ILS^AQR^; Extended Data Fig. 8c), U6 snRNA forms an internal stem loop and pairs through its ACAGA region with the intron 5′ SS, forming the U6–5′ SS helix^44–46^. This tethers the 5′ SS to the RNA active site through approximately 2.5 helical turns (Fig. 3e). In the DIS cryo-EM density, however, the U6 internal stem loop is not visible, suggesting that it is mobile or unwound, and only one helical turn of the U6–5′ SS helix remains (Fig. 3d). Although we did not resolve RNA density in the DHX35 active site, the U6 snRNA regions 3′ of the ACAGA sequence are well positioned to dock into it. DHX35 could then translocate along the U6 snRNA in the 3′-to-5′ direction, unwinding the remaining helical turn of the U6–5′ SS helix to eject the intron and disassemble the spliceosome (Fig. 3c,d).

This predicts that the DIS can disassemble without additional factors. We therefore immobilized the purified DIS on beads via GFP-tagged DHX35, supplied ATP and analysed the released proteins (Extended Data Fig. 8d). Indeed, the U5 snRNP, NTC, NTR, but not DHX35, GPATCH1 or WDR83, were released upon ATP addition (Extended Data Fig. 8e,f and Supplementary Table 5). To probe the role of DHX35 in this assay, we also purified DIS complexes using the catalytically inactive GFP–DHX35 mutant (K83A). These spliceosomes were compositionally indistinguishable from the wild-type DIS (Extended Data Fig. 7g and Supplementary Table 2), indicating that the DIS forms before DHX35 action. Adding ATP to the immobilized DIS containing DHX35(K83A) did not release spliceosome proteins, supporting a role for DHX35 in disassembly (Extended Data Fig. 8e and Supplementary Table 5). These data indicate that DHX35 may translocate along U6 snRNA to unwind the partial U6–5′ SS duplex, ejecting the debranched intron for decay and freeing the spliceosome for recycling.

## Recognition of defective spliceosomes

In addition to successful pre-mRNA splicing, defective spliceosomes erroneously assembled on aberrant pre-mRNA introns must also terminate during spliceosome quality control (SQC)^47–49^. How such defective human spliceosomes are recognized and terminated has remained unclear, although both DHX15 and DHX35 have been implicated^50–54^. Recent structures of *Schizosaccharomyces* *pombe* and *Chaetomium thermophilum* spliceosomes^5,23^ revealed both helicases in the same SQC complex, corresponding to a defective B* spliceosome (B*^D^) stalled before branching on introns with an aberrant 5′ SS^5,23^. In these structures, DHX15 and DHX35 are poised to act on U6 and U2 snRNAs, respectively, to terminate the defective complex and reject the pre-mRNA. Given the structural homology between *S. pombe* and human splicing machineries^8,23,53^ and our ILS^AQR^ and DIS data, DHX15 and DHX35 may be central to ejecting both excised introns and aberrant pre-mRNAs from the spliceosome.

**Fig. 4:**
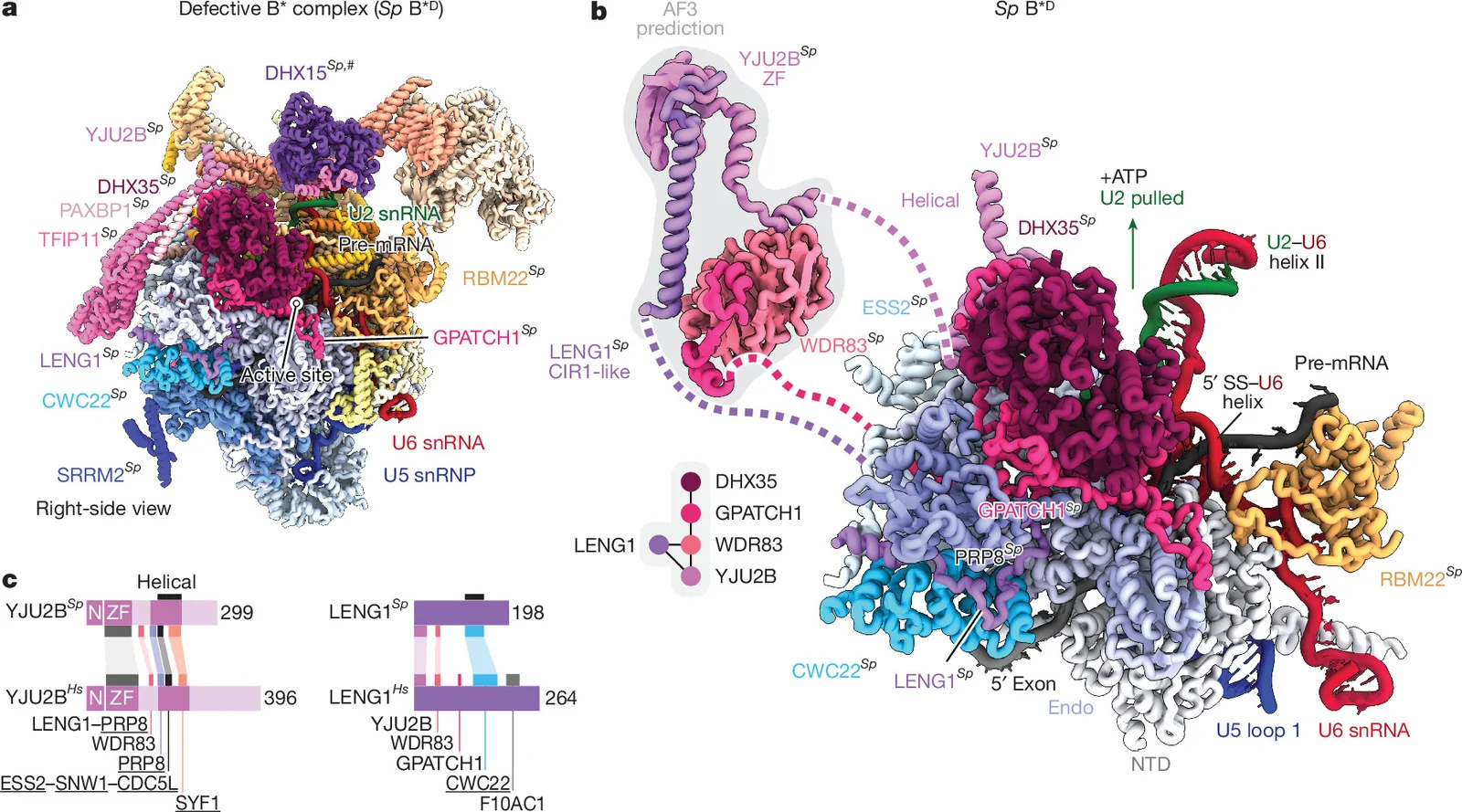
Targeting of defective spliceosomes for termination. **a**, Revised structure of the B*^D^ from *S. pombe*^23^ reveals YJU2B and LENG1 as B*^D^-specific proteins, and includes additions for GPATCH1 (*S. pombe* name C20H4.06c), CDC5L (Cdc5), CCDC12 (Cwf18) and SNW1 (Prp45). DHX15 was modelled based on its position in *C. thermophilum*^5^ B*^D^ and the ILS structures from human (here and refs. ^2,3^), *Saccharomyces cerevisiae*^4^ and *C. elegans*^2^, as well as its presence in the *S. pombe* B*^D^ complex^5^. *Sp*, *Schizosaccharomyces pombe*. **b**, Interactions of termination factors within the revised B*^D^ and view of its RNA network. An AF3 prediction of the *S. pombe* LENG1–YJU2B–WDR83–GPATCH1 complex is shown in a grey outline and connected to the experimental structure by dotted lines. *Hs*, *Homo sapiens*. **c**, Conserved domain architectures and interactomes of human and *S. pombe* YJU2B and LENG1. Interactors observed in cryo-EM structures are underlined. Protein regions observed in the structure of the *S. pombe* B*^D^ are marked with black lines above the *S. pombe* diagrams. The inset (right) summarizes the predicted interactions among the newly identified termination factors in humans and their *S. pombe* counterparts (black lines).

**Extended Data Fig. 9:**
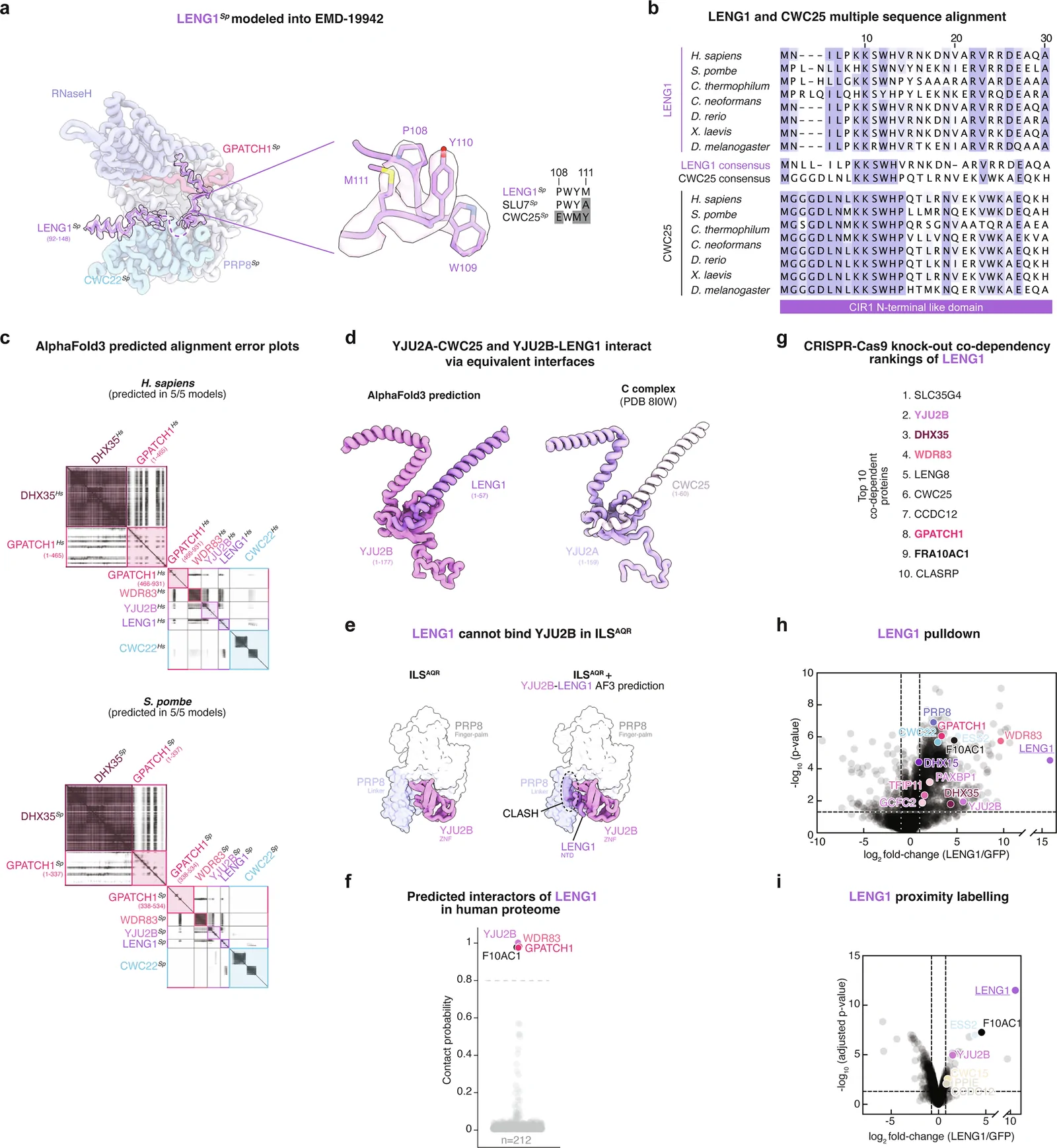
LENG1 is an SQC factor. **a**. An unknown density in the *S. pombe* B*^D^ spliceosome belongs to the uncharacterized protein LENG1. Left: Overall fit of the modelled LENG1^*Sp*^ region (*S. pombe* residues 92-148) in the previously unassigned density of the cryo-EM map of the *S. pombe* B*^D^ spliceosome (also known as B^d^-II; EMD-199942). LENG1^*Sp*^ runs along the surface of CWC22^*Sp*^ and comes into contact with the RNaseH domain of PRP8^*Sp*^. Right: Close-up view of the cryo-EM density segment that could be unambiguously assigned as the PWYM motif of LENG1^*Sp*^. SLU7^*Sp*^ and CWC25^*Sp*^ contain highly similar motifs, depicted in a sequence alignment on the right. However, in CWC25^*Sp*^ the two aromatic residues are not consecutive, and in SLU7^*Sp*^ the final residue in the motif is alanine, which is too small to fit into the observed density. As a summary, the residues of CWC25^*Sp*^ and SLU7^*Sp*^ that are incompatible with the observed density are marked in grey in the sequence alignment. **b**. Multiple sequence alignment (MSA) of the N-terminal CIR1-like domain of LENG1 and CWC25. A comparison of the consensus sequences of LENG1 and CWC25 is also shown. Both proteins are highly conserved across eukaryotes, and their shared sequence features reveal LENG1 and CWC25 as paralogues. Residues are coloured according to their BLOSUM62 conservation scores. **c**. Predicted alignment error (PAE) plots of the DHX35–GPATCH1–WDR83–YJU2B–LENG1–CWC22 complex predictions from AlphaFold3 with human (left), *S. pombe* (right) proteins. Each binary interaction was confidently predicted in five out of five models. *S. pombe* was chosen, because the DHX35-mediated spliceosome quality control pathway has been shown to occur in this species^5,23^. **d**. Comparison of the AlphaFold3 prediction of the YJU2B-LENG1 dimer and the experimental structure of the YJU2A-CWC25 interface in the C complex spliceosome (PDB 8I0W)^56^. The two pairs of paralogues are likely to interact via equivalent interfaces. YJU2B-LENG1 may discriminate aberrant spliceosomes from on-pathway complexes associated with YJU2A-CWC25. **e**. Overlay of the YJU2B-PRP8 interface determined experimentally as part of ILS^AQR^ (left) with the YJU2B-LENG1 AlphaFold3 prediction (right), showing that LENG1 would clash with PRP8 and could not stably engage with ILS^AQR^. We did not observe the YJU2B–LENG1 interface in B*^D^ densities, possibly owing to mobility. **f**. Identification of putative LENG1 interactors – YJU2B, WDR83, GPATCH1, FRA10AC1 (F10AC1) – in a human proteome-wide in silico interaction screen^20^. ‘n’ equals the number of candidate interactors for each protein. Contact probability is defined in ref. ^20^. **g**. List of the top 10 highest ranked co-dependent proteins of LENG1 in CRISPR-Cas9 knock-out viability screens, according to the DepMap database^24^ (June 2026). Co-dependency means that the indicated proteins are required for growth in the same set of cancer cell lines, which suggests that LENG1, DHX35, YJU2B, WDR83 and GPATCH1 may function in a common cellular pathway. **h**. Pulldown of ectopically expressed GFP-tagged LENG1 coupled to quantitative mass spectrometry in human cells. The bait protein is underlined. Putative SQC factors (DHX35, GPATCH1, WDR83, YJU2B, TFIP11, PAXBP1, GCFC2), predicted LENG1 interactors detected in the proximity labelling experiment in panel i (ESS2, FRA10AC1), and general spliceosome factors (PRP8, CWC22) are enriched in the LENG1 pulldown. Differences between groups were statistically evaluated with two-sided t-tests. The horizontal dashed line marks the significance threshold of p = 0.05. The vertical dashed lines indicate an enrichment threshold of log_2_-fold change of 1 (2-fold enrichment on a linear scale) over the nuclear GFP control. Each experiment was carried out in biological quadruplicates. **i**. Proximity labelling using TurboID fused to LENG1 coupled to quantitative mass spectrometry in human cells. The bait protein is underlined. Spliceosomal proteins FRA10AC1 (F10AC1), ESS2 and, importantly, YJU2B are highly enriched with several other spliceosomal proteins (CWC15, PPIE, CCDC12) detected at lower levels. Differences between groups were statistically evaluated using limma with a moderated two-sided t-test. The multiple testing correction used the Benjamini-Hochberg procedure. The horizontal dashed line marks the significance threshold of p = 0.05, the vertical dashed line indicates an enrichment threshold of log_2_-fold change of 0.75 (68% enrichment on a linear scale) over the TurboID-tagged GFP control. Each experiment was carried out in biological triplicates.

To explore the parallels between regular and defective termination, we revised the cryo-EM structure of the *S. pombe* defective B*^D^ complex (also named B^d^-II)^23^ (Fig. 4a). We identified the *S. pombe* protein Saf4 as the functional orthologue of YJU2B^23^ (Fig. 4c and Extended Data Fig. 6a) and assigned several unidentified densities (Fig. 4a,b), including an uncharacterized protein: LENG1 (*S. pombe* C5E4.10c; Extended Data Fig. 9a). LENG1 runs along the C-terminal domain of CWC22, a catalytic-stage spliceosome subunit absent from ILS and DIS complexes (Fig. 4a). LENG1 is a paralogue of the essential branching factor CWC25 (Extended Data Fig. 9b). AlphaFold3 predicts a high-confidence YJU2B–LENG1 complex for both human and *S. pombe* orthologues, homologous to the YJU2–CWC25 heterodimer in the regular C complex^55,56^ (Fig. 4b and Extended Data Fig. 9c,d). In the ILS^AQR^, the position of the YJU2B zinc-finger domain is incompatible with the predicted LENG1 interaction (Extended Data Fig. 9e) and TFIP11 occupies the CWC22-binding site^2^, which could explain the absence of LENG1 from regular terminating spliceosomes (Fig. 2e).

Given the role of YJU2B in guiding DHX35–GPATCH1–WDR83 to the ILS^AQR^, we explored whether YJU2B–LENG1 may recruit these factors to the B*^D^. Supporting this, LENG1 is confidently predicted to bind to GPATCH1, WDR83 and YJU2B^20^ (Extended Data Fig. 9f), through interfaces compatible with the B*^D^ structure (Fig. 4b). These factors and DHX35 have been previously detected by mass spectrometry in the human C complex^57–60^ and rank among the top LENG1 co-dependencies in human CRISPR–Cas9 screens^24,25^ (Extended Data Fig. 9g). Moreover, GFP-tagged LENG1 co-immunoprecipitated multiple spliceosome factors from human cells, including CWC22 and the SQC-related factors YJU2B, DHX35, GPATCH1 and WDR83, corroborated by LENG1 proximity labelling data (Extended Data Fig. 9h,i and Supplementary Tables 4 and 6). LENG1 may thus act with YJU2B to guide DHX35 via GPATCH1–WDR83 towards SQC complexes. Consistently, our RNA sequencing from native DHX35–GFP pulldowns revealed reads spanning intron–exon junctions (Fig. 3h and Extended Data Fig. 7d,f). The native DHX35 purification could thus be heterogeneous. Although the regular DIS represents the predominant state, DIS-like states could have arisen from defective spliceosomes stalled either before, as in the B*^D^, or after branching. The roles of these SQC factors, and the full scope of states they target, await further investigation.

## Mechanisms of regular and defective spliceosome termination

**Fig. 5:**
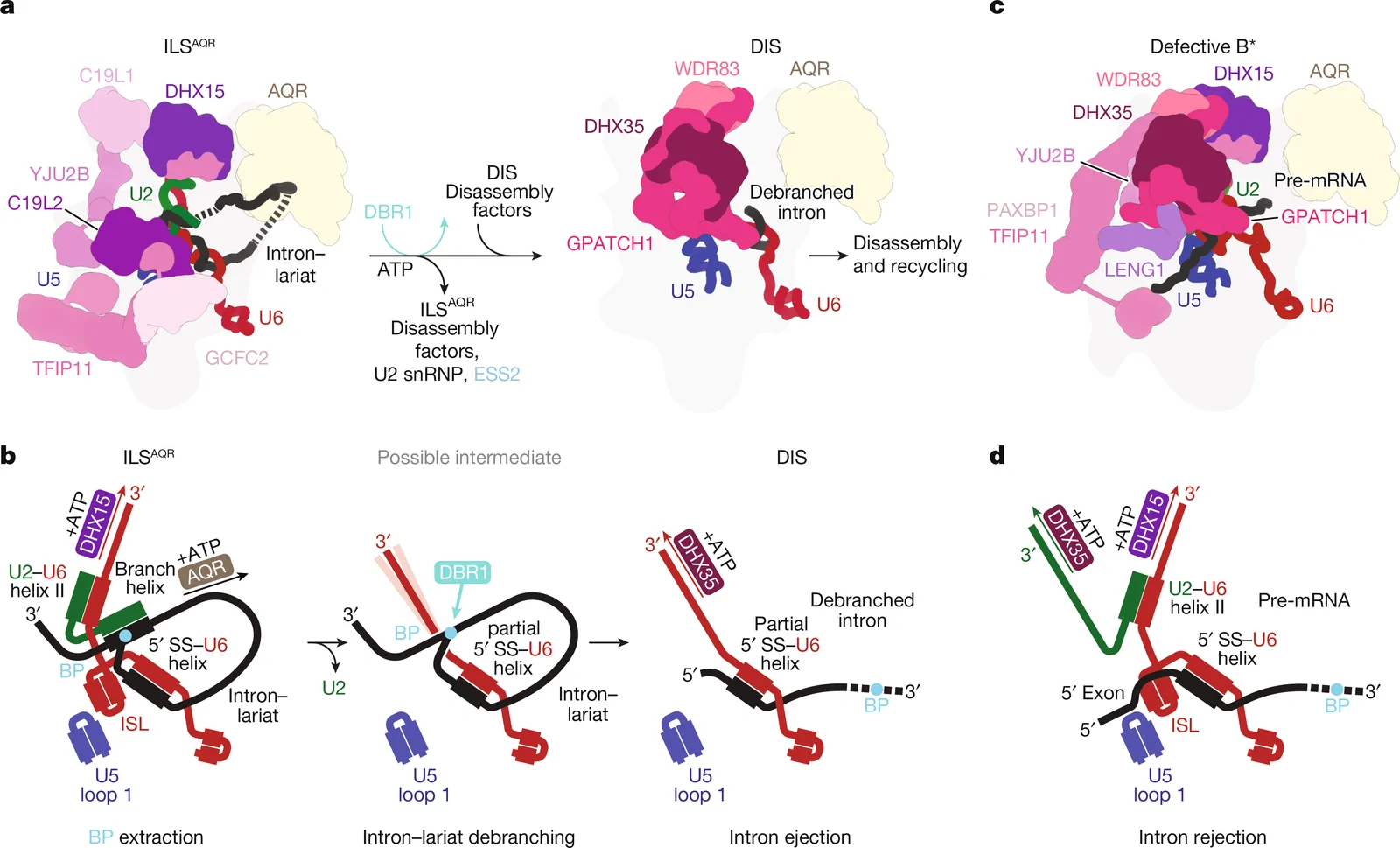
Mechanisms of regular and defective spliceosome termination. **a**, Model of regular spliceosome termination, highlighting ILS^AQR^ and DIS termination factors. **b**, Comparison of ILS^AQR^ and DIS RNA networks shows how three RNA helicases—DHX15, AQR and DHX35—remodel the RNA-based active site in at least two steps, to extract, debranch and eject the excised intron and to disassemble the spliceosome. In a possible intermediate, the extracted intron–lariat BP is engaged with the enzyme DBR1 for debranching on the spliceosome (middle panel). **c**, Termination factors bound to B*^D^ reveal how seven factors shared with the regular termination pathway and the B*^D^-specific LENG1 may facilitate the disassembly of defective spliceosomes. **d**, The B*^D^ RNA network shows how RNA helicases DHX15 and DHX35 would reject an aberrant intron to terminate a defective spliceosome.

The involvement of DHX15 and DHX35 in both regular and defective spliceosome termination highlights mechanistic parallels between these pathways. Both helicases bind simultaneously to the defective B*^D^ complex^5,23^ (Fig. 4a), whereas in regular termination, they are distributed across two sequential states: the ILS^AQR^ and the DIS (Fig. 5a,c). DHX15 occupies the same position in B*^D^ as in ILS complexes^2–5^, loaded onto the U6 snRNA 3′ end. The DHX35–GPATCH1 interfaces are indistinguishable between the DIS and B*^D^ (Fig. 5a,c), but owing to their different RNA network configurations, DHX35 would act on U2 snRNA in the B*^D^ and on U6 snRNA in the DIS. In the B*^D^, DHX35 might also act sequentially on U2 and U6 snRNAs. Moreover, C19L1 and C19L2, which are critical for regular termination, are absent from the defective B*^D^. Conversely, LENG1 would act only in defective spliceosomes, contributing to their recognition for SQC, although the precise mechanism remains to be determined.

We propose that these different RNA network configurations may explain the differences between regular and defective termination. During regular termination, the U2–intron branch helix is stabilized by the ILS-specific C19L2, which buries the branched intron BP in the RNA active site and occludes the PRP8 surface needed for the subsequent binding of DHX35–GPATCH1, thus necessitating ATP-mediated remodelling to form the DIS. This stepwise dismantling may be necessary for debranching on the spliceosome, providing a window in which C19L1-tethered DBR1 can act on the extracted intron–lariat BP (Fig. 5b). By contrast, in the B*^D^ during defective termination, the U2–intron branch helix has already been disrupted^23^ and the intron has not yet undergone branching. Therefore, temporal separation of DHX15 and DHX35 activities would be dispensable, allowing single-step disassembly during SQC (Fig. 5c).

Together, we have captured native human spliceosomes during termination, revealing how three RNA helicases—AQR, DHX15 and DHX35—and their co-factors orchestrate the gradual dismantling of the spliceosomal RNA network, coupling intron–lariat debranching and ejection to regular spliceosome disassembly. We extended this paradigm to defective termination, in which DHX15 and DHX35 are aided by an overlapping but distinct set of factors to reject aberrant introns. Our results show how both regular and defective spliceosomes terminate specifically and efficiently, sustaining the processing of protein-coding mRNAs and the fidelity of gene expression.

## Methods

### ILS disassembly assay in nematode extract

#### Sample preparation

Total nematode extract was prepared from a *C. elegans* strain expressing endogenously 3×FLAG-tagged PRP19 (ref. ^2^).

Disassembly assays in the presence and absence of ATP were performed in technical triplicates. A total of 500 µl nematode extract was supplemented with either 5× splicing buffer with ATP (50 mM HEPES-KOH pH 7.9, 175 mM KCl, 10 mM ATP, 20 mM MgCl_2_, 100 mM creatine phosphate, 250 µg ml^−1^ creatine kinase and 15% PEG 8000) or 5× splicing buffer without ATP (50 mM HEPES-KOH pH 7.9, 175 mM KCl and 20 mM MgCl_2_). The supplemented extracts were incubated at 20 °C for 1 h with gentle agitation. Of magnetic anti-FLAG bead slurry (Sigma) equilibrated in detergent wash buffer (20 mM HEPES-KOH pH 7.9, 150 mM KCl, 1 mM TCEP, 2 mM MgCl_2_, 0.05% Igepal 630 and 10% glycerol), 20 µl was then added to each reaction. The samples were transferred to 4 °C and incubated with the beads for 2 h with gentle rotation. The beads were washed four times with 1 ml detergent wash buffer, transferred to fresh tubes and then washed an additional four times with detergent-free wash buffer (20 mM HEPES-KOH pH 7.9 and 150 mM KCl). The washed beads were resuspended in detergent-free wash buffer, transferred to fresh tubes and submitted to the Vienna BioCenter Mass Spectrometry Facility for on-bead tryptic digestion and liquid chromatography–tandem mass spectrometry (LC–MS/MS) analysis.

#### Assay analysis

The resulting raw MS data were processed for protein identification and label-free quantification. To identify the proteins that were depleted from the beads upon ATP treatment, protein abundances in the samples with and without ATP were compared. Proteins were considered significantly enriched based on statistical tests (moderated *t*-test in limma, *P* < 0.05) and an arbitrary log_2_ fold change cut-off of 1 (twofold enrichment over control on a linear scale). The data were visualized in GraphPad Prism 10.

### Generation of human cell lines

#### K562 cells stably overexpressing DHX35–EGFP or YJU2B–EGFP

N-terminally EGFP–3C-tagged DHX35 and YJU2B overexpression cell lines were generated using a lentivirus system. Lenti-X 293T cells (Takara) were polyethylenimine transfected with lentiviral carrier plasmids containing either pRRL-SFFV-EGFP-3C-DHX35 or pRRL-SFFV-YJU2B-3C-EGFP. These were transfected alongside viral helper plasmids pCMVR8.74 (Addgene #22036) and pCMV-VSV-G (Addgene #8454). After 2 days, supernatant containing the lentiviral particles was collected. Wild-type K562 cells (DSMZ) were infected at limiting dilutions and EGFP-expressing cells were isolated by fluorescence-activated cell sorting using a BD FACSAria III instrument (BD Biosciences). Integration and expression of the constructs were confirmed by in-gel EGFP fluorescence and immunoblotting for DHX35 (CAS HPA062700, Sigma; dilution 1:250) or YJU2B (CAS OAAB02156, Aviva SysBio; dilution 1:1,000). All cultures were routinely tested for mycoplasma contamination.

#### K562 cells inducibly overexpressing DHX35(K83A)–EGFP or DBR1–EGFP

To generate inducible cell lines, C-terminally 3C–EGFP-tagged DBR1 and N-terminally EGFP–3C-tagged mutant DHX35(K83A) constructs were cloned into a PiggyBac transposon donor plasmid containing a doxycycline-inducible TRE Tight promoter. Wild-type K562 cells (DSMZ) were electroporated with equal amounts of transposon donor and PiggyBac transposase plasmids using a Neon NxT Electroporation System (NEON1S, Thermo). Cells were selected using blasticidin (R21001, Invitrogen). Expression of the transgene was induced using 0.1 µg ml^−1^ doxycyclin (D9891, Sigma-Aldrich) for 18 h and EGFP^+^ cells were isolated using fluorescence-activated cell sorting. Expression of the constructs was confirmed by in-gel EGFP fluorescence and immunoblotting for DBR1 (CAS PA5-30616, Invitrogen; dilution 1:500) or DHX35 (CAS HPA062700, Sigma; dilution 1:250). All cultures were routinely tested for mycoplasma contamination.

#### HEK293T cells with endogenous DBR1–dTAG

HEK293T cells were endogenously tagged at the C terminus of DBR1 with GGGS_3_-FKBP12(F36V)-V5-IRES-lox5171-mScarlet3/mNeonGreen-lox5171, where FKBP12 is the protein that brings DBR1 to the cellular ubiquitination machinery in the presence of the dTAG^V^-1 compound, and lox5171 is a Cre recombinase target site. An sgRNA targeting the DBR1 C terminus was designed using CHOPCHOP^61^ and cloned into a modified plasmid #217659 (Addgene), in which the mCherry coding sequence was replaced with a puromycin resistance cassette. The sgRNA plasmid was co-transfected with two donor plasmids. The donor plasmids contained the repair template with the desired tag followed by either mScarlet3 or mNeonGreen and flanked by the DBR1 homology arms. Five days after transfection, cells co-expressing mScarlet3 and mNeonGreen, which indicated homozygous tagging, were sorted by flow cytometry and then allowed to recover for 4 days. The cells were then treated with homemade recombinant Cre recombinase for 2 h at 37 °C in serum-free medium to excise the coding sequences of the fluorescent protein markers from the DBR1 loci. After 3 days of recovery, mScarlet3/mNeonGreen double negative cells were single-cell sorted into medium supplemented with extra 25% of FCS. The selected clones were expanded and genotyped by Sanger sequencing. The homozygous clones were selected, and protein depletion upon addition of the dTAG^V^-1 compound was validated by western blot using an antibody to DBR1 (CAS 66951-1-IG, Proteintech; dilution 1:10,000).

To generate HEK293T DBR1–dTAG cell lines that also inducibly overexpress various DBR1 constructs (wild type, catalytic mutant (H85A) and deletion of peptide I residues 420–434 (Δpep1)), the coding sequences of DBR1 were cloned into the PiggyBac transposon donor plasmid under a doxycycline-inducible TRE Tight promoter. The DBR1 constructs were tagged on the C terminus with a P2A self-cleaving peptide followed by mScarlet. The DBR1-P2A–mScarlet expression cassettes were inserted into the HEK293T DBR1–dTAG cells by electroporation, and the successful clones were selected by fluorescence-activated cell sorting, as described above for the DHX35(K83A)–GFP and DBR1–GFP K562 overexpression cell lines. All cell lines were routinely tested for mycoplasma contamination.

#### HEK293T cells inducibly overexpressing TurboID-tagged DBR1, C19L1, YJU2B, LENG1 or EGFP

To generate stable inducible cell lines for proximity labelling experiments, HEK293 Flp-In T-REx parental cells at approximately 80–90% confluency were co-transfected with a pcDNA5/FRT/TO plasmid, which carried the coding sequence of DBR1, C19L1, YJU2B, LENG1 or nuclear localized EGFP fused on the 3′ end to the TurboID-V5 tag, and the pOG44 plasmid (Thermo Fisher Scientific) at a 1:5 mass ratio (donor:pOG44) using the JetOptimus transfection reagent (Polyplus-transfection). For a 24-well plate format, 350,000 cells were seeded per well onto the transfection complex. Twenty-four hours after transfection, cells were expanded into a six-well plate. Forty-eight hours later, selection was initiated. The cells were seeded to achieve 25–30% confluency and allowed to attach for approximately 2 h. The medium was then replaced with selection medium containing 100 µg ml^−1^ hygromycin B and 15 µg ml^−1^ blasticidin S HCl. The selection medium was refreshed every 3–4 days. The selection process was monitored using the parallel wild-type cell culture, and selection was continued until all wild-type cells had died (approximately 10–14 days), at which point the pooled colonies of resistant cells were expanded without antibiotics. Successful integration was confirmed by western blotting using an antibody to the V5 tag (CAS R960-25, Thermo Fisher Scientific, dilution 1:3,000). All cultures were routinely tested for mycoplasma contamination.

#### RPE-1 cells stably overexpressing LENG1–EGFP

cDNA corresponding to LENG1 was amplified from cDNA by PCR and inserted into a PGK-EGFP-N1-IRES-PURO plasmid, thereby tagging LENG1 at its C terminus with EGFP. The annealed double-stranded DNA fragment of NLS (GGTGGTCCTAAGAAGAAGAGAAAGGTT) was similarly cloned into PGK-EGFP-C1-IRES-PURO as a control.

The plasmids expressing the tagged proteins were transfected into RPE-1 cells^62^ using polyethylenimine reagent (Brunschwig Chemie) following the manufacturer’s instructions. Two days after transfections, antibiotic selection of cells was initiated by adding 1 μg ml^−1^ puromycin to the growth media. The selection medium was replaced every second day until the cells reached confluency. The expression of tagged proteins was verified by western blot using an antibody to GFP (CAS 11814460001, Roche, dilution 1:1,000) and fluorescence microscopy. All cultures were routinely tested for mycoplasma contamination.

### Preparation of human nuclear extract

The nuclear extracts for cryo-EM, RNA sequencing and biochemical assays were prepared from the human K562 cells stably overexpressing EGFP-tagged DHX35 or YJU2B as previously described^2,63^. To prepare nuclear extract from the K562 cells overexpressing EGFP-tagged DHX35(K83A) or DBR1, expression of the tagged protein was first induced by addition of 0.1 µg ml^−1^ doxycycline. The cells were cultured for 48 h before collecting and preparing nuclear extract as previously described^2,63^.

### Cryo-EM grid preparation

#### Purification of DIS (DHX35-containing spliceosome)

Approximately 25 ml of the nuclear extract of K562 cells stably overexpressing DHX35–EGFP was applied to approximately 1.5 ml of GFP-Trap Agarose beads (Chromotek) equilibrated in wash buffer (20 mM HEPES-KOH pH 7.9, 100 mM KCl, 0.5 mM TCEP, 2 mM MgCl_2_, 0.05% v/v Igepal CA-630 and 8% v/v glycerol). The beads were incubated with the nuclear extract for approximately 3.5 h at 4 °C with gentle rotation. The beads were then washed five times in 10 ml wash buffer. The washed beads were incubated twice for approximately 1 h at 4 °C with gentle rotation in 0.5 ml elution buffer (20 mM HEPES-KOH pH 7.9, 100 mM KCl, 0.5 mM TCEP, 2 mM MgCl_2_, 3% w/v sucrose and 0.1 µg ml^−1^ 3C protease). The eluted samples were then applied to a GraFix^64^ 15–30% w/v sucrose gradient containing 0.05% glutaraldehyde in the heaviest fraction in a gradient buffer (20 mM HEPES-KOH pH 7.9, 100 mM KCl, 0.5 mM TCEP and 2 mM MgCl_2_). The gradient samples were spun in an SW60 Ti rotor (Beckman Coulter) at 22,000 rpm for 16 h at 4 °C. The gradients were fractionated manually, and each fraction was quenched with 45 mM lysine. The sedimentation coefficients were estimated using the CowSuite software, and the peak spliceosome fractions were further confirmed by SDS–PAGE. The peak spliceosome fractions were pooled and concentrated using Amicon Ultra 0.5-ml concentrators with 100-kDa molecular weight cut-off (Sigma). During concentrating, the sample was buffer exchanged to a cryo-EM buffer (20 mM HEPES-KOH pH 7.9, 0.5 mM TCEP, 100 mM KCl and 2 mM MgCl_2_) to remove sucrose and excess glutaraldehyde.

#### Purification of ILS^AQR^ (YJU2B-containing spliceosome)

Approximately 25 ml of the nuclear extract of K562 cells stably overexpressing YJU2B–EGFP was incubated overnight with 2 µg ml^−1^ benzonase before proceeding to anti-GFP affinity purification and gradient ultracentrifugation as described above for DHX35-containing spliceosome.

#### Grid preparation

The purified spliceosome samples were incubated with a 2-nm continuous carbon film (prepared in-house) for either overnight (DIS), approximately 7 h (ILS^AQR^; dataset 1) or approximately 3 h (ILS^AQR^; dataset 2) in a homemade humidity chamber at 4 °C. The carbon film was picked up with a glow-discharged R3.5/1 holey carbon grid (Quantifoil). Of ice-cold cryo-EM buffer, 3 µl was applied to the grid before blotting at 4 °C and 80% humidity and plunging into liquid ethane using a Leica EM GP2 instrument.

### Cryo-EM data collection

#### ILS^AQR^ or YJU2B -containing spliceosome

Two datasets of ILS^AQR^ were collected using a Titan Krios G4 equipped with a Falcon 4i direct electron detector (Vienna BioCenter). Dataset 1 contained 26,091 movies, whereas dataset 2 consisted of 27,327 movies. The energy filter was set to a width of 10 eV. The C2 aperture was set to 50 µm, whereas the objective aperture was retracted. In both datasets, the movies were collected at a total dose of 40 e^−^ Å^−^^2^ in EER format with a pixel size of 0.951 Å per pixel. The target defocus of both datasets ranged from −1.0 µm to −2.5 µm.

#### DIS or DHX35-containing spliceosome

A dataset of 12,277 micrographs was collected using a Titan Krios G4 equipped with a Falcon 4i direct electron detector (Vienna BioCenter). The energy filter was set to a width of 10 eV. The C2 aperture was set to 50 µm, whereas the objective aperture was retracted. The movies were collected at a total dose of 50 e^−^ Å^−^^2^ in EER format with a pixel size of 0.951 Å per pixel. The target defocus ranged from −1.25 µm to −2.25 µm.

### Cryo-EM data analysis

#### Pre-processing and particle cleaning of human ILS^AQR^

Pre-processing (gain correction, motion correction and contrast transfer function estimation) of both datasets of the ILS^AQR^ was performed in cryoSPARC live^65^.

For dataset 1, 931,992 particles were picked on-the-fly using the Blob picker in cryoSPARC live with a minimum and maximum particle diameter set to 250 Å and 450 Å, respectively. The picked particles were extracted with a box size of 128 pixels at a binned pixel size of 4.01 Å per pixel. A 2D classification was performed to select the particles representing 2D class averages with clear internal features. The selected particles were subsequently used to generate three 3D ab initio models. Three rounds of heterogeneous refinement were performed using the ab initio models as references. Only the particles from the best class were carried over to the next round of refinement, resulting in the final subset of 232,423 particles. Particles in dataset 1 were also picked in Warp^66^ using a model trained on manually picked particles with an expected particle diameter of 350 Å. The 1,172,730 Warp-picked particles were processed the same as above, resulting in a subset of 294,689 particles. Upon removal of duplicate particles, the combined Blob-picked and Warp-picked particles were re-extracted with a box size of 448 pixels at a binned pixel size of 1.15 Å per pixel and further processed by reference-based motion correction in cryoSPARC. In the end, 310,532 particles were obtained from dataset 1.

For dataset 2, particles were picked in Warp^66^ using a model trained on manually picked particles with an expected particle diameter of 350 Å. The picked 1,068,365 particles were extracted with a box size of 128 pixels with a binned pixel size of 4.01 Å per pixel. The three ab initio models from dataset 1 were used for three rounds of heterogeneous refinement with only the best class being carried over to the next round of refinement. The selected particles were then re-extracted with a box size of 448 pixels at a binned pixel size of 1.15 Å per pixel and further processed by reference-based motion correction in cryoSPARC. In the end, 197,936 particles were obtained from dataset 2.

#### Obtaining maps of human ILS^AQR^

The particles from both datasets were combined, resulting in a complete set of 508,031 particles. A non-uniform refinement generated a consensus map of the ILS^AQR^ (map 1) at a global resolution of 2.40 Å. By performing a local refinement with a mask around the core of the spliceosome, we obtained map 2 at a resolution of 2.34 Å. To further improve the resolution of specific spliceosome regions, we performed local refinements with masks around the zinc-finger domain of YJU2B (map 3 at a nominal resolution of 2.32 Å), the helical bundle of the NTR subunits PRP19–CDC5L–SPF27 (map 9 at a nominal resolution of 3.83 Å), the helical repeats of the NTC subunits SYF1–SYF2 (map 10 at a nominal resolution of 2.59 Å) and the U5 Sm ring (map 11 at a nominal resolution of 2.88 Å).

The GCFC domains and the coiled-coil interaction interface of TFIP11 and GCFC2 were poorly resolved in map 1, probably due to their flexibility and potentially their partial occupancy. To better resolve these termination factors in the ILS^AQR^, we performed two rounds of 3D classification with a mask around TFIP11–GCFC2 on the combined 508,031 particles. First, we performed a hard 3D classification in cryoSPARC. The 139,130 particles from the best 7 out of 50 classes were selected for a 3D classification in RELION5 with Blush regularization^67^ with a *T* value of 40. The resulting best class containing 6,794 particles was locally refined in cryoSPARC with a mask around TFIP11–GCFC2 to generate map 8 at a nominal resolution of 7.37 Å.

For certain spliceosomal regions, combining particles from two datasets did not result in significant improvements in density quality. Specifically, to produce ILS^AQR^ maps 4–7, only the 310,532 particles from dataset 1 were used.

To improve the resolution of the termination factor C19L2, we performed a local refinement with a mask around C19L2, resulting in map 4 at a nominal resolution of 2.84 Å.

To better visualize AQR within ILS^AQR^, we performed a hard 3D classification on the particles from dataset 1 with a mask around AQR. Particles from 3D classes with the best-defined AQR density were used in a local refinement with a mask around AQR before proceeding to 3D variability analysis in cryoSPARC with the same mask. The 46,099 particles from the best cluster out of 10 were used for another local refinement with a mask around AQR, generating map 5 at a nominal resolution of 3.92 Å.

The region of ILS^AQR^ containing the helical repeats of SYF1, the MPP domain of C19L1 and the interacting helix of YJU2B appeared to be flexible and, hence, poorly resolved in map 1. To better visualize this region, we performed 3D variability analysis of the 310,532 particles from dataset 1 after homogeneous refinement with a global mask. One out of 10 clusters with the best-defined region of interest containing 26,265 particles was selected and further locally refined with a mask around SYF1–C19L1–YJU2B. This produced map 6 at a nominal resolution of 6.32 Å.

To better resolve DHX15 that appeared to be flexibly tethered to the ILS^AQR^, we performed several 3D classification jobs varying the class similarity values with a mask around DHX15. The best classes from several classification jobs were combined, duplicate particles were removed and locally refined with a broad mask around DHX15 and the surrounding regions, and further 3D classified with the same broad mask. The 9,659 particles from the two best classes were further locally refined with a mask around DHX15, resulting in map 7 at a nominal resolution of 8.62 Å.

#### Pre-processing and particle cleaning of human DIS

Pre-processing (gain correction, motion correction and contrast transfer function estimation) of the DIS dataset was performed in cryoSPARC live^65^.

Particles were picked on-the-fly using the Blob picker in cryoSPARC live with a minimum and maximum particle diameter set to 250 Å and 350 Å, respectively. The picked particles were extracted with a box size of 256 pixels with a binned pixel size of 1.90 Å per pixel. A 2D classification was performed to select the particles representing 2D class averages with the best internal features. The selected particles were subsequently used to generate three ab initio 3D models. Three rounds of heterogeneous refinement were performed using the ab initio models as references. Only the particles from the best class were carried over to the next round of refinement, resulting in the final subset of 140,678 particles. DIS particles were also picked in Warp^66^ using a model trained on manually picked particles with an expected particle diameter of 350 Å. The 514,099 Warp-picked particles were processed the same as above, resulting in a subset of 130,283 particles. Upon removal of duplicate particles, the combined Blob-picked and Warp-picked particles were re-extracted with a box size of 448 px with a binned pixel size of 1.19 Å per pixel, resulting in the final dataset of 153,470 particles.

#### Obtaining maps of human DIS

We used all 153,470 particles to perform a non-uniform refinement and obtained the overall consensus map of the DIS (map 1) at a global resolution of 3.14 Å. Although the core of the DIS in map 1 could be visualized at high resolution, many peripheral spliceosome regions were poorly defined and required further 3D classifications and local refinements to obtain high-quality cryo-EM densities.

To improve the density of the intron–U6 snRNA duplex, we performed a 3D classification with all 153,470 particles with a mask around the RBM22–intron–U6 snRNA region. The best class from this classification was further refined using non-uniform refinement, resulting in map 2 at a nominal resolution of 4.67 Å.

To improve the density quality of the WD40 domain of PRP17, we performed a local refinement around this domain and obtained map 3 at a nominal resolution of 3.97 Å.

To improve the density quality of DHX35–GPATCH1, we imported all 153,470 particles to RELION5 and performed a 3D classification with Blush regularization^67^ with a mask around DHX35–GPATCH1 and a *T* value of 150. The best class from the 3D classification containing 63,636 particles was imported back into cryoSPARC. After homogeneous reconstruction, a local refinement with a mask around DHX35–GPATCH1 was performed, generating map 4 at a nominal resolution of 2.90 Å.

We performed a 3D classification on all 153,470 particles to assess the homogeneity of the dataset. Although we did not observe multiple distinct spliceosome complexes in the dataset, we identified 45,958 particles that yielded a DIS map with the highest quality peripheral densities. We performed a non-uniform refinement on these particles to obtain a more complete DIS map at a nominal resolution of 3.62 Å with better peripheral density quality than map 1. We used the same subset of particles to perform local refinements with masks around two NTC regions—the PRP19–CDC5L–SPF27 helical bundle and the RBM22–PPIL1–PLRG1 region—resulting in map 5 at a nominal resolution of 5.35 Å and in map 9 at a nominal resolution of 4.04 Å, respectively.

To better visualize AQR within the DIS, we performed a hard 3D classification with a mask around AQR, producing a class with the best AQR density with 15,913 particles. After homogeneous reconstruction, a local refinement with a mask around AQR was performed, generating map 6 at a nominal resolution of 7.34 Å.

We consistently observed a low-resolution density near DHX35 that appeared to be shaped like a WD40 domain. Given our biochemical data and in silico protein interaction predictions (Fig. 1 and Extended Data Fig. 1), we assigned this density as the GPATCH1 interactor WDR83. To improve this cryo-EM density, we performed multiple rounds of 3D classification. Specifically, we performed five 3D classification jobs with variable values of class similarity with a mask around the putative WDR83 density in cryoSPARC. The best classes from all jobs were selected. After removing duplicate particles, the selected particles were used in two rounds of 3D classification in RELION5 with a mask around WDR83 at *T* values of 80 for the first round and of 40 during the second round. The best class containing 1,826 particles was reconstructed using homogeneous reconstruction in cryoSPARC, generating map 7 at a nominal resolution of 9.65 Å. Given the limited resolution of map 7, we could not explicitly assign the orientation of WDR83 in the DIS.

In maps 1 and 2, the helical repeats of the NTR subunits SYF1–SYF3 and the Sm ring of the U5 snRNP were not well resolved, probably due to the inherent flexibility of these spliceosomal regions. Therefore, we performed focus refinements with masks around SYF1–SYF3 and the U5 Sm ring, resulting in map 8 at a nominal resolution of 5.19 Å and map 10 at a nominal resolution of 3.35 Å, respectively.

### Model building into cryo-EM maps

#### Human ILS^AQR^

We generated the composite map of the human ILS^AQR^ from ILS^AQR^ maps 1–11 as previously described^2^.

To prepare a model of the human ILS^AQR^, we started from our previously published model of the human ILS″ (PDB 8RO2)^2^. First, we removed the following protein segments that were not observed in the cryo-EM density of the ILS^AQR^: SDE2, SYF1 residues 791–824, CDC5L residues 57–111 and 357–382. To model the zinc-finger domain of YJU2B (residues 43–118), we predicted its structure together with a Zn^2+^ ion using AlphaFold3 (ref. ^36^) and rigid body fitted the predicted model of this domain. To model the helical region of YJU2B (residues 163–233), we predicted the structures of the YJU2B–SYF1–SYF3–C19L1 and YJU2B–PRP8 complexes in AlphaFold3. Both models were rigid body fitted. We also added BRR2 residues 39–57 (as in PDB 8Q7X)^9^, PPIE RRM residues 1–80 (as in PDB 9FMD)^2^ by rigid body fitting. We also modelled additional ESS2 regions (residues 65–87, 178–189, 211–234 and 240–254) guided by AlphaFold3 predictions and ModelAngelo^68^. The RNA in the active site of AQR was modelled as an unknown RNA sequence. The position of DHX15–TFIP11 G-patch was adjusted by rigid body fitting into map 10. PAXBP1 was replaced with GCFC2 by first deleting the TFIP11–PAXBP1 dimer from the model and then fitting an AlphaFold3 prediction of the TFIP11–GCFC2 dimer into map 6. We truncated side chains for non-proline residues to their Cβ atoms in map regions where side-chain density was lacking.

We modelled the RNA in the active site based on the structure of the human ILS assembled on the MINX pre-mRNA (PDB 6ID1)^3^ but only assigned a sequence for a consensus 5′ SS and a BP adenosine, whereas all other nucleotides were modelled as uridine and truncated to their ribose moiety. The portion of the intron bound to AQR and PPIE was modelled based on AlphaFold3 predictions.

All models were manually adjusted using ISOLDE^69^ in ChimeraX. The final model was real-space refined against the composite map in Phenix^70^. We generated base-pairing and stacking restraints in Phenix to stabilize nucleic acid geometry. We further used the input model to generate reference model restraints. The non-bonded weight parameter was set to 1,000 and rotamers were fitted for side chains with poor density that were also outliers.

#### Human DIS

We generated the composite map of the human DIS from maps 1–10 as previously described^2^.

To prepare a model of the DIS, we started from our previously published model of the human ILS″ (PDB 8RO2)^2^. First, we deleted DHX15, TFIP11, PAXBP1, C19L1, C19L2, SDE2, ESS2 and U2 snRNA, which were not resolved in the cryo-EM density of the DIS. We also truncated U6 snRNA to residues 1–35 and the intron to 10 residues, six of which were base paired with U6 snRNA. Given that a mixture of introns is present in the endogenous DIS sample, no sequence was assigned to the intron.

To model DHX35 and GPATCH1, we predicted the structures of DHX35–GPATCH1, GPATCH1–PRP8 endonuclease domain and the GPATCH1–PRP8 finger-palm domain using AlphaFold2 Multimer^37^. The regions of GPATCH1 that were predicted with low confidence were truncated, and the predicted models were rigid body fitted into map 7. The segments of GPATCH1 towards its C terminus that were confidently predicted to interact with DHX35 but lacked experimental cryo-EM densities were also removed. The fitted models were adjusted using ISOLDE^69^ in ChimeraX. The segments of GPATCH1 that were not confidently predicted but were present in the cryo-EM density were manually built in ISOLDE.

The RNaseH domain of PRP8, which was relocated in the DIS compared with the ILS″, was rigid body fitted into the DIS map. BRR2 residues 39–57 (as in PDB 8Q7X)^9^ and the RRM domain of PPIE (residues 1–80; as in PDB 9FMD)^2^ were added by rigid body fitting followed by manual adjustments in ISOLDE.

We also modelled the WD40 domains of PRP17 and of WDR83 bound to GPATCH1. The orientation of the PRP17 WD40 was assigned based on its position relative to the PRP17 N-terminal domain that anchors the protein on the surface of BUD31. We rigid body fitted the predicted AlphaFold2 Multimer model of WDR83–GPATCH1 into map 9. Owing to the low local resolution of map 9, we could not unambiguously assign the orientation of WDR83–GPATCH1. The resolution of map 9 was also not sufficient to unambiguously determine whether WDR83 was bound by GPATCH1. However, our biochemical data (Extended Data Fig. 6d) and AlphaFold predictions (Fig. 1 and Extended Data Fig. 1) strongly indicate that WDR83 is anchored by GPATCH1 in the DIS. We truncated side chains for non-proline residues to their Cβ atoms in map regions where side-chain density was lacking.

The final model was real-space refined against the composite map in Phenix^70^ as described above for ILS^AQR^.

#### Revision of *S. pombe* B*^D^

To revise the structure of the defective fission yeast spliceosome B*^D^ (PDB 9ESI)^23^, we first focused on identifying the protein chains that were originally modelled as ‘unknown’. We compared the published model to our models of the human ILS^AQR^ and DIS. We noted that all but one of the unidentified chains were modelled in our new human spliceosome structures. We predicted the structures of the *S. pombe* proteins and their interactors in AlphaFold3 (ref. ^36^) and rigid body fitted them into the published map (EMD-19942)^23^. This allowed us to add CDC5L (*S. pombe* name Cdc5) residues 456–476, CCDC12 (Cwf18) residues 101–142 and GPATCH1 (C20H4.06c) residues 260–280.

We also identified an unassigned density on the surface of PPIL1 in the B*^D^ spliceosome that was modelled as an N-terminal domain of SNW1 in our previously revised human P complex spliceosome (PDB 9FMD)^2^. We predicted the structure of *S. pombe* SNW1–PPIL1 (*S. pombe* names Prp45–Ppi1), rigid body fitted the model into the published map and further adjusted it manually using ISOLDE^69^. This allowed us to add SNW1 (Prp45) residues 42–83 to the model of the *S. pombe* B*^D^ spliceosome.

To identify the unknown density on the surface of CWC22 (*S. pombe* name Cwf22), we examined the previously published spliceosome structures for proteins modelled in the same location, as well as searched the human predicted interactome database^20^ for CWC22 interactors. The *S. pombe* orthologues of the proteins that were identified as potential CWC22 interactors were then further tested for their predicted interaction with *S. pombe* CWC22 using AlphaFold3. This narrowed down the list of potential candidates to three proteins: CWC25 (*S. pombe* name Cwf25), SLU7 (Slu7) or LENG1 (SPCC5E4.10c). As described in the main text and in Extended Data Fig. 9a, in the published map, we observed a segment of four residues with unambiguous side-chain densities: P-Y/F-W followed by an amino acid with a side chain of at least two non-hydrogen atoms. Only the sequence of LENG1 matched the unambiguously resolved side-chain density (Extended Data Fig. 9a). The *S. pombe* CWC22–LENG1 prediction was rigid body fitted into the published map and then manually adjusted in ISOLDE, using a more confident prediction of the human proteins as a guide.

We also adjusted the RNA network of the B*^D^ spliceosome. Specifically, we noticed implausible base-pairing geometry in the 5′ SS–U6 snRNA region, and adjusted the model based on an earlier *S. pombe* spliceosome structure (PDB 3JB9)^71^. Finally, we truncated intron nucleotides that were poorly resolved and outside of known consensus sequences to their ribose moiety.

We truncated side chains for non-proline residues to their Cβ atoms in map regions where side-chain density was lacking. The final model was real-space refined against the published map (EMD-19942)^23^ in Phenix^70^ as described above.

### Endogenous DBR1 pulldown

Pulldowns with nuclear extracts from both induced and uninduced (negative control) K562 doxycycline-inducible DBR1–EGFP cells were performed in technical triplicates. Of nuclear extract, 1 ml was added to the GFP Trap beads slurry (Chromotek) equilibrated in wash buffer (20 mM HEPES-KOH pH 7.9, 50 mM, 0.5 mM TCEP, 2 mM MgCl_2_, 0.05% v/v Igepal CA-630 and 8% v/v glycerol). The beads were incubated for 3.5 h at 4 °C with gentle rotation. The supernatant was removed, and the beads were washed five times with 1 ml wash buffer. After removing residual buffer, bead-bound proteins were eluted by addition of 150 µl SDS loading dye. All three replicates of the pulldowns from extracts of both induced and control cells were run for 3 min at 180 V on a Novex Tris-glycine 4–12% 1.0 mm WedgeWell gel (Thermo Fisher Scientific) and submitted to the Vienna BioCenter Mass Spectrometry Facility for in-gel tryptic digestion and LC–MS/MS analysis. Protein abundances in both DBR1–GFP and control samples were compared, and the data were visualized in GraphPad Prism 10.

### Endogenous LENG1 pulldown

Cell pellets were solubilized in EBC-1 (50 mM Tris pH 7.5, 150 mM NaCl, 0.5% NP-40, 2 mM MgCl_2_, with protease and phosphatase inhibitor cocktails (Roche)) supplemented with 500 U benzonase for 1 h at 4 °C on a rotator. The lysates were cleared from insoluble chromatin by centrifugation and were subjected to immunoprecipitation with GFP Trap beads (Chromotek) for 1.5 h at 4 °C under rotation. The beads were then washed five times with EBC-2 buffer (50 mM Tris pH 7.5, 150 mM NaCl, 0.5% NP-40 and 1 mM EDTA) and boiled in Laemmli buffer for 10 min at 95 °C.

After the pulldown, the GFP beads were washed three times with 50 mM ammonium bicarbonate, followed by overnight digestion using 0.5 μg trypsin at 37 °C under constant shaking. Digested peptides were separated from the beads using a 0.45-µm filter column (Merck) that was pre-washed with 50 mM ammonium bicarbonate. Trypsin activity was quenched by acidifying the sample with trifluoroacetic acid to a final concentration of 1%. Peptides were desalted and concentrated using in-house-assembled triple-disc C18 stage-tip columns (66883-U; 3 M) as previously described^72^. In brief, the columns were first washed with methanol, then with buffer B (80% acetonitrile and 0.1% formic acid in water), followed by buffer A (0.1% formic acid in water). The samples were loaded using vacuum centrifugation. Finally, peptides were eluted with 32% acetonitrile and 0.1% formic acid in water, lyophilized and dissolved in 0.1% formic acid in water. All solvents were LC–MS/MS grade.

Mass spectrometry data were acquired at the proteomics facility of the Andalusian Center for Molecular Biology and Regenerative Medicine (CABIMER) using a Vanquish Neo liquid chromatographer (Thermo Scientific) coupled to an Orbitrap Astral mass spectrometer. Mass spectrometry RAW data were analysed using DIA-NN (v2.3.2 Academia)^73^. Protein abundances in both LENG1–GFP and control samples were compared, and the data were visualized in GraphPad Prism 10.

### Proximity labelling

#### Large-scale proximity labelling and affinity purification

For proteomic analysis, each stable HEK293 Flp-In T-REx cell line that can inducibly overexpress a TurboID-tagged protein of interest (DBR1, C19L1, YJU2B or LENG1) and a control cell line (nuclear EGFP–TurboID) were processed in biological triplicates. Three million cells per 10-cm dish were seeded. After 24 h, TurboID expression was induced with 100 ng ml^−1^ doxycycline for another 24 h. This was followed by biotin labelling with 500 µM d-biotin for 15 min. After labelling, the reaction was stopped by placing dishes on ice. The medium was removed, and the cells were washed once with PBS. The cells were then collected by scraping them off in 5 ml ice-cold PBS and transferred to a fresh tube. The cell pellet was washed for a total of four more times by centrifugation at 300*g* for 4 min at 4 °C before getting resuspended in 1 ml ice-cold PBS. The final pellets were flash-frozen in liquid nitrogen and stored at −80 °C.

The thawed cell pellets were lysed by addition of 120 µl ice-cold RIPA buffer with Halt protease inhibitor cocktail (Thermo Fisher Scientific), followed by vigorous vortexing and incubation on ice for 20 min. The lysates were clarified by centrifugation at 20,000*g* for 15 min at 4 °C. Protein concentration in the supernatant was determined using the Pierce BCA Protein Assay Kit (Thermo Fisher Scientific). Of total protein, 300 µg from each lysate was incubated for 3 h at 4 °C with 30 µl of the MagReSyn Streptavidin MS magnetic bead slurry (ReSyn Biosciences) equilibrated in ice-cold RIPA buffer. The beads were washed three times with RIPA buffer supplemented with a protease inhibitor cocktail (Thermo Fisher Scientific) with a 10-min rotation for each wash. This was followed by one wash in MS-grade PBS, during which the bead slurry was transferred to a fresh low-bind tube to minimize detergent carryover. Finally, five additional washes were performed with MS-grade PBS. After the final wash, all residual PBS was removed, and the dry bead pellets were flash-frozen in liquid nitrogen and stored at −80 °C.

#### MS and data analysis

The final washed bead pellets were submitted either to the Vienna BioCenter Mass Spectrometry Facility or to the Max Perutz Labs Mass Spectrometry Facility on dry ice for on-bead tryptic digestion and analysis by LC–MS/MS. The resulting raw data were processed for protein identification and label-free quantification. To identify high-confidence proximity partners, protein abundances in each bait-TurboID sample were statistically compared against those from the control samples (nuclear-localized EGFP–TurboID). Proteins were considered significantly enriched based on statistical tests (moderated *t*-test in limma, *P* < 0.05) and an arbitrary log_2_ fold-change cut-off of 0.75 (approximately 68% enrichment over control on a linear scale). Data visualization was performed in GraphPad Prism 10.

### Purification of recombinant proteins

#### C19L1 MPP domain

The recombinant human C19L1 MPP domain (residues 1–278), which carried an N-terminal His_10_-MBP tag followed by a 3C protease cleavage site, was expressed in *Escherichia coli* BL21 DE3 in LB media at 18 °C overnight using a pOPINB^+^ plasmid.

The cell pellet was resuspended in lysis buffer (25 mM HEPES-NaOH pH 7.9, 500 mM NaCl and 5% glycerol) supplemented with cOmplete protease inhibitor tablets (Roche). The cells were lysed by sonication, and the lysate was cleared by centrifugation. The lysate was then applied to a 5-ml MBP Trap HP column (Cytiva) equilibrated in lysis buffer. The column was washed with lysis buffer before elution of the protein with a step gradient of 12 mM maltose. The eluate was diluted fivefold with buffer A (25 mM HEPES-NaOH and 5% glycerol) to reduce the salt concentration in the sample to 100 mM NaCl. The diluted sample was applied to a 5-ml HiTrap Q anion exchange column (Cytiva) equilibrated in buffer M (25 mM HEPES-NaOH, 100 mM NaCl and 5% glycerol). The column was washed with buffer M before elution of the protein over a linear gradient of 100–800 mM NaCl. The peak fractions were concentrated using a Sartorius centrifugal concentrator with a 50-kDa molecular weight cut-off (Sigma) and subsequently loaded onto a HiLoad Superdex 75 16/60 size-exclusion column (Cytiva) equilibrated in size-exclusion buffer (25 mM HEPES-NaOH pH 7.9, 250 mM NaCl, 1 mM dithiothreitol and 5% glycerol). The peak size-exclusion fractions were pooled, concentrated and flash-frozen in liquid nitrogen.

#### DHX35

Human DHX35 was cloned into a pGB10 plasmid with an N-terminal His_10_−3×FLAG tag followed by a 3C protease cleavage site. The plasmid was introduced into *E. coli* DH10EMBacY cells to generate bacmids^74^. The purified bacmid was transfected into Sf9 insect cells to produce a baculovirus that was then further amplified in Sf9 cell. The resultant virus was used to infect Hi5 insect cells at a volume ratio of 1:400. The cells were grown for approximately 72 h before collecting by centrifugation.

The cell pellet was resuspended in lysis buffer (25 mM HEPES pH 7.9, 500 mM NaCl, 5% glycerol and 20 mM imidazole) supplemented with cOmplete protease inhibitor tablets (Roche) and DNaseI (Sigma). The cells were broken open by sonication. The lysate was cleared by two rounds of centrifugation and subsequently applied to a 5-ml HisTrap column (Cytiva) equilibrated in lysis buffer. The column was washed with lysis buffer, and the protein was eluted over a linear gradient of 20–350 mM imidazole. The peak fractions containing DHX35 were pooled and diluted fivefold in buffer A (25 mM HEPES-NaOH and 5% glycerol) to reduce the salt concentration in the sample to 100 mM NaCl. The diluted sample was then loaded onto a 5-ml HiTrap Q anion exchange column (Cytiva) equilibrated in buffer M (25 mM HEPES-NaOH, 100 mM NaCl and 5% glycerol). The column was washed with buffer M before elution of the protein over a linear gradient of 0.1–1.4 M NaCl. The peak fractions were concentrated using a Sartorius centrifugal concentrator with a 50-kDa molecular weight cut-off (Sigma) and subsequently loaded onto a HiLoad Superdex 200 16/60 size-exclusion column (Cytiva) equilibrated in size-exclusion buffer (25 mM HEPES-NaOH pH 7.9, 250 mM NaCl, 1 mM dithiothreitol and 5% glycerol). The peak size-exclusion fractions were pooled, concentrated and flash-frozen in liquid nitrogen.

#### GPATCH1 G-patch domain

The recombinant human wild-type and mutant (M163K V171R L236E) versions of the GPATCH1 G-patch domain (residues 137–247), which carried an N-terminal MBP tag and a C-terminal His_6_ tag, were expressed in *E. coli* BL21 DE3 in LB media at 37 °C for 3 h using a pET plasmid.

Each cell pellet was resuspended in lysis buffer (25 mM HEPES-NaOH pH 7.9, 500 mM NaCl, 5% glycerol and 20 mM imidazole) supplemented with cOmplete protease inhibitor tablets (Roche). The cells were lysed by sonication, and the lysate was cleared by centrifugation. The lysate was then applied to a 5-ml HisTrap column (Cytiva) equilibrated in lysis buffer. The column was washed with lysis buffer before elution of the protein over a linear gradient of 20–350 mM imidazole. The eluate was diluted fivefold with buffer A (25 mM HEPES-NaOH and 5% glycerol) to reduce the salt concentration in the sample to 100 mM NaCl. The diluted sample was applied to a 5-ml HiTrap Q anion exchange column (Cytiva) equilibrated in buffer M (25 mM HEPES-NaOH, 100 mM NaCl and 5% glycerol). The column was washed with buffer M before elution of the protein over a linear gradient of 100–800 mM NaCl. The peak fractions were concentrated using a Vivaspin centrifugal concentrator with a 30-kDa molecular weight cut-off (Sigma) and subsequently loaded onto a HiLoad Superdex 200 16/60 size-exclusion column (Cytiva) equilibrated in size-exclusion buffer (25 mM HEPES-NaOH pH 7.9, 200 mM NaCl, 1 mM dithiothreitol and 5% glycerol). The peak size-exclusion fractions were pooled, concentrated and flash-frozen in liquid nitrogen.

#### WDR83–GPATCH1 complex

Human full-length WDR83 carrying an N-terminal His_10_-3×FLAG-3C tag and its binding region of human GPATCH1 (residues 560–651) were cloned into a pGB10 plasmid. The plasmid was then used to generate a baculovirus and to express the complex in Hi5 insect cells, as described above for DHX35.

The cell pellet was resuspended in lysis buffer (25 mM HEPES-NaOH pH 7.9, 300 mM NaCl, 1 mM TCEP, 5% glycerol and 20 mM imidazole) supplemented with cOmplete protease inhibitor tablets (Roche) and DNaseI (Sigma). The cells were broken open by sonication. The lysate was cleared by two rounds of centrifugation and subsequently applied to a 5-ml HisTrap column (Cytiva) equilibrated in lysis buffer. The column was washed with lysis buffer, and the protein was eluted over a linear gradient of 20–350 mM imidazole. The peak fractions containing WDR83–GPATCH1 were pooled and diluted fivefold in buffer A (25 mM HEPES-NaOH, 0.5 mM TCEP and 5% glycerol) to reduce the salt concentration in the sample to 100 mM NaCl. The diluted sample was then loaded onto a 5-ml HiTrap Q anion exchange column (Cytiva) equilibrated in buffer M (25 mM HEPES-NaOH, 100 mM NaCl, 0.5 mM TCEP and 5% glycerol). The column was washed with buffer M before elution of the protein over a linear gradient of 0.1–1.4 M NaCl. The peak fractions were concentrated using an Amicon centrifugal concentrator with a 30-kDa molecular weight cut-off (Sigma). Of the WDR83–GPATCH1 complex at 79 µM, 2 ml was subsequently loaded via a 5-ml loop onto a HiLoad Superdex 200 16/60 size-exclusion column (Cytiva) equilibrated in size-exclusion buffer (25 mM HEPES-NaOH pH 7.9, 200 mM NaCl, 0.5 mM TCEP and 5% glycerol). SDS–PAGE analyses of each chromatographic run showed that WDR83 and GPATCH1 co-purified throughout the procedure, indicating the formation of a stable protein complex. The peak size-exclusion fractions were pooled, concentrated and flash-frozen in liquid nitrogen.

#### DHX35(K83A)–GPATCH1 1–440 complex

Full-length human DHX35 carrying a K83A mutation and an N-terminal His_10_-3×FLAG tag, and the GPATCH1 construct encompassing the binding regions to both DHX35 and PRP8 (residues 1–440) were cloned into a pGB10 plasmid. The plasmid was then used to generate a baculovirus and express the complex in Hi5 insect cells.

The 3×FLAG–DHX35(K83A)–GPATCH1(1−440)–TwStrep complex was purified as described for the WDR83–GPATCH1 complex above, except for the size-exclusion step, in which the DHX35(K83A)–GPATCH1(1−440) complex was loaded onto a HiLoad Superdex 200 16/60 size-exclusion column (Cytiva) equilibrated in size-exclusion buffer (25 mM HEPES-NaOH pH 7.9, 250 mM NaCl, 0.5 mM TCEP and 5% glycerol).

### Peptide synthesis

The peptides used in in vitro pulldown assays were synthesized at the Vienna BioCenter Peptide Synthesis facility as previously described^2^. In brief, the peptides were synthesized with an N-terminal fluorescein and a C-terminal biotin tag on a Liberty Blue peptide synthesizer (CEM) using Fmoc chemistry. The lyophilized peptides were dissolved in 25 mM HEPES-NaOH pH 7.9 to a concentration of 1 mg ml^−1^. The following peptides were synthesized: DBR1 peptide I (420–434) wild type: fluo-ALSSINPDEIMLDEE-biotin; DBR1 peptide I (420–434) scrambled: fluo-EEIEMISLADDLSNP-biotin; YJU2B wild type (134–178): fluo-EHEKKQKLETDAMFRLEHGEADRSTLKKALPTLSHIQEAQSAWKD-biotin; YJU2B(M146A L149K E150K) (134–178): fluo-EHEKKQKLETDAAFRKKHGEADRSTLKKALPTLSHIQEAQSAWKD-biotin.

### Pulldowns with purified proteins

#### MBP–C19L1 MPP domain and DBR1 peptide I

Of MBP-tagged C19L1 MPP, 5 µM was mixed with 50 µM of either wild-type DBR1 peptide I or its scrambled version in the final reaction volume of 50 µl. Reactions containing either MBP–C19L1 MPP alone or the DBR1 peptides mixed with MBP as background binding controls were also prepared. The salt concentration in all reactions was adjusted to 100 mM NaCl. Of each sample, 5 µl was removed for SDS–PAGE analysis as input. The binding reactions were incubated for 40 min at 4 °C while rotating. Subsequently, the samples were added to 30 µl of amylose bead slurry (NEB) equilibrated in wash buffer (25 mM HEPES-NaOH pH 7.9, 100 mM NaCl, 1 mM TCEP, 0.1% v/v Igepal CA-630 and 5% v/v glycerol). The beads were incubated for 2 h rotating at 4 °C. The supernatant was removed, and the beads were washed five times with 500 µl wash buffer. Upon removal of residual buffer, 50 µl elution buffer (25 mM HEPES-NaOH pH 7.9, 50 mM NaCl, 0.5 mM TCEP, 5% v/v glycerol and 20 mM maltose) was added to each sample and incubated at 4 °C for 30 min while rotating. The eluates were collected, mixed with SDS loading dye and ran on a Novex Tris-Glycine 4–12% 1.0 mm WedgeWell gel (Thermo Fisher Scientific) for 20 min at 180 V. The DBR1 peptides were visualized by fluorescence signal at 488 nm on a ChemiDoc imager (Bio-Rad), whereas MBP–C19L1 MPP and MBP were stained afterwards with Coomassie blue (Vienna BioCenter Molecular Biology Service).

#### DHX35 and MBP–GPATCH1 G-patch domain

Of DHX35, 4 µM was mixed with 1 µM of either wild-type or mutant (M163K V171R L236E) GPATCH1 G-patch domain (residues 137–247) in the final reaction volume of 50 µl. Reactions containing individual proteins were also prepared as background binding controls. The salt concentration in all reactions was adjusted to 50 mM NaCl. All samples were incubated at 4 °C for 35 min while rotating. Of each sample, 5 µl was removed for SDS–PAGE analysis as input. Subsequently, each sample was added to 40 µl of amylose bead slurry (NEB) equilibrated in wash buffer (25 mM HEPES-NaOH pH 7.9, 50 mM NaCl, 0.5 mM TCEP, 0.03% v/v Igepal CA-630 and 5% v/v glycerol). The proteins were incubated with the beads for 1.5 h at 4 °C while rotating. The beads were then washed five times with 0.5 ml wash buffer. After removing residual wash buffer, 50 µl of wash buffer supplemented with 40 mM maltose was added to the beads and incubated for 1 h at 4 °C while rotating. The eluates were collected, mixed with SDS loading dye and ran on a Novex Tris-Glycine 4–12% 1.0 mm WedgeWell gel (Thermo Fisher Scientific) for 30 min at 180 V. The gel was stained with Coomassie blue (Vienna BioCenter Molecular Biology Service).

#### WDR83–GPATCH1 and biotin-tagged YJU2B peptide

Of either wild-type or mutant (M146A L149K E150K) YJU2B peptide (134–178), which was predicted to interact with WDR83, 80 µl was added at 25 µM to 80 µl StrepTactin Sepharose bead slurry (IBA) equilibrated in wash buffer (25 mM HEPES-NaOH pH 7.9, 50 mM NaCl, 1 mM TCEP, 5 mM MgCl_2_, 0.05% v/v Igepal CA-630 and 10% v/v glycerol). The beads were incubated with the peptides for 1 h at 4 °C while rotating and then washed four times with 0.4 ml of wash buffer. The washed beads containing the immobilized peptides were split into two samples and incubated either with or without 80 µl of the WDR83–GPATCH1(560–651) complex at 29 µM. The WDR83–GPATCH1(560–651) complex was also incubated with the beads lacking immobilised peptides as a background binding control. The salt concentration in all samples was adjusted to 50 mM NaCl and incubated at 4 °C for 2 h while rotating. The beads were then washed five times with 0.5 ml wash buffer. After removal of residual wash buffer, 80 µl of wash buffer supplemented with 50 mM biotin was added to each reaction and incubated for 1 h at 4 °C while rotating. The eluates were collected, mixed with SDS loading dye and ran on a Novex Tris-Glycine 4–12% 1.0 mm WedgeWell gel (Thermo Fisher Scientific) for 25 min at 180 V. The YJU2B peptides were visualized by fluorescence signal at 488 nm on a ChemiDoc imager (Bio-Rad). The gel was then stained with Coomassie blue (Vienna BioCenter Molecular Biology Service) to visualize WDR83. The band corresponding to GPATCH1 in a Coomassie-stained gel could not be unambiguously identified, because its band overlaps with the bands of the YJU2B peptides.

### DBR1 depletion rescue experiment

HEK293T homozygous DBR1–dTAG-V5 cells containing the doxycycline-inducible mScarlet-tagged DBR1 constructs (wild type, catalytic mutant (H85A) and deletion of peptide I residues 420–434 (Δpep1)) as well as a control line lacking DBR1 overexpression were mixed 1:1 with wild-type HEK293T cells that constitutively expressed GFP. Cells were split into two wells: one well was treated with the 500 nM dTAG^v^-1 compound, whereas the other well was left untreated to serve as a control. Expression of the DBR1 constructs was induced using 1 µg ml^−1^ doxycycline. Cells were split at a ratio of 1:3 every day to prevent them from reaching confluency. The percentage of GFP^+^ cells was measured using an iQue Screener Plus high-throughput cytometer (Sartorius AG) immediately after mixing and again after 8 days. The average percentage of GFP^−^ cells per line was calculated from technical triplicates across three biological replicates and served as a proxy for cell growth phenotype.

Relative expression levels of the inducible DBR1 constructs were measured as the mScarlet signal in each well after 1 day, when the GFP signal, and hence, cell viability, did not differ across conditions. The average mScarlet signal was then normalized to that of the wild-type DBR1 construct.

### In vitro ILS^AQR^-to-DIS remodelling assay

Of nuclear extract prepared from K562 cells stably overexpressing EGFP-tagged YJU2B, 120 µl was incubated with the recombinant 3×FLAG–DHX35(K83A)–GPATCH1(1−440)–TwStrep complex at various final concentrations (0, 0.5 µM or 4.5 µM) either in the presence or absence of 5 mM ATP. The reactions were incubated at 37 °C for 40 min before being applied to 45 µl GFP Trap bead slurry (Chromotek) equilibrated in wash buffer (20 mM HEPES-KOH pH 7.9, 100 mM KCl, 0.5 mM TCEP, 2 mM MgCl_2_, 0.05% v/v Igepal CA-630 and 8% v/v glycerol) supplemented with 2 mg ml^−1^ BSA. The binding reactions were incubated for 1.5 h at 4 °C. The beads were then washed three times with wash buffer and twice with harsh wash buffer (20 mM HEPES-KOH pH 7.9, 200 mM KCl, 0.5 mM TCEP, 2 mM MgCl_2_, 0.1% v/v Igepal CA-630 and 8% v/v glycerol). The proteins immobilized on the beads were eluted in 60 µl detergent-free wash buffer (20 mM HEPES-KOH pH 7.9, 100 mM KCl, 0.5 mM TCEP, 2 mM MgCl_2_, 8% v/v glycerol) supplemented with 0.1 µg ml^−1^ 3C protease for 1 h at 4 °C.

The eluates from all samples were then analysed by western blot using standard protocols with the antibodies to spliceosome proteins YJU2B (CAS OAAB02156, Aviva Systems Biology; dilution 1:100), PRP8 (CAS AB190347, Abcam; dilution 1:1,000), BRR2 (CAS AB241589, Abcam; dilution 1:1,000), AQR (CAS A302-546A, Bethyl Laboratories; dilution 1:1,000), TFIP11 (CAS PA5-49275, Invitrogen; dilution 1:1,000), C19L1 (CAS 31700-1-AP, Proteintech; dilution 1:5,000) and C19L2 (CAS 24872-1-AP, Proteintech, dilution 1:200). 3×FLAG–DHX35(K83A) and GPATCH1(1−440)–TwStrep were detected using antibodies to FLAG (CAS F3165, Sigma-Aldrich; dilution 1:500) and Strep (CAS 2-1509-001, iba; dilution 1:10,000) tags, respectively.

### RNA sequencing assay

#### Extraction of spliceosome-associated RNA

For RNA sequencing, endogenous human DIS was purified by anti-GFP affinity purification and gradient ultracentrifugation as described above for cryo-EM sample preparation, except that no glutaraldehyde was added to the gradient and that the pooled spliceosome-containing gradient fractions were not concentrated.

Endogenous human ILS″ for the deletion rate experiment and endogenous human ILS^AQR^ for the read truncation experiment were purified from K562 cells stably overexpressing TFIP11–GFP or YJU2B–GFP, respectively, using anti-GFP affinity purification and gradient ultracentrifugation as described above and previously^2^, except that no glutaraldehyde was added to the gradient and the peak gradient fractions were not concentrated.

The pooled spliceosome fractions were incubated with 0.4 mg ml^−1^ proteinase K in 2 mM EDTA and 0.04% SDS for 1.5 h at 37 °C with gentle shaking. A 0.1 volume of 7.5 M ammonium acetate was added to the sample and mixed thoroughly. One volume of phenol-chloroform solution (ITW reagents) was added to the sample and briefly vortexed. The sample was spun down at 20,000*g* for 5 min. The top aqueous layer containing nucleic acids was transferred to a fresh tube, mixed with 2 volumes of isopropanol and 0.002 volumes of GlycoBlue co-precipitant (Thermo Fisher Scientific), and incubated overnight at −20 °C. The sample was then centrifuged at 20,000*g* for 5 min at 4 °C. The supernatant was removed, and the remaining pellet was washed in ice-cold 70% ethanol. The washed pellet was briefly air dried and resuspended in 10 µl nuclease-free water.

#### Preparation of short-read sequencing libraries (read truncation experiment)

For each sample, 3 µl of RNA was polyadenylated by incubating in a reaction containing 2 µl 5×SSIV reaction buffer (Invitrogen), 0.25 µl *E. coli* polyA polymerase (NEB), 0.25 µl RNasin (Promega), 0.25 µl 0.1 M dithiothreitol (Invitrogen) and 0.5 µl 0.5 mM ATP (Invitrogen) at 37 °C for 15 min. The samples were placed on ice, diluted with 9.25 µl water, then heated at 95 °C for 4 min to fragment the RNA. To anneal reverse transcription primers, 1 µl of 10 mM dNTPs and 1 µl of 10 µM dT(22)V-P7 primer were added, and the samples were heated to 65 °C for 5 min before cooling to 42 °C. To initiate reverse transcription, 2 µl of 5×SSIV reaction buffer (Invitrogen), 0.5 µl 0.1 M dithiothreitol (Invitrogen) and 0.5 µl SSIV enzyme (Invitrogen) were added to the samples, and the reactions were incubated at 42 °C for 20 min. The reactions were cooled to 37 °C and incubated with 2 µl exonuclease I (NEB) for 15 min to remove excess reverse transcription primers. To further remove excess RT primers, the samples were size selected using SPRI beads with a final PEG8000 concentration of 7.5% and a final isopropanol concentration of 22.5%. The samples were eluted from the beads, then RNA was hydrolysed by the addition of 1 µl 1 M NaOH and incubated at 95 °C for 10 min. The pH of the samples was then neutralized by the addition of 1 µl 1 M HCl and 10 µl 1 M HEPES pH 7.9. The cDNA was then purified using 2 volumes of SPRI beads and 3 volumes of isopropanol. The cDNA was eluted in 5.3 µl water, to which 1 µl DMSO and 2 µl 10 µM L01clip2.0 adapter was added. The mixture was heated to 75 °C for 2 min and then placed on ice. Adapter ligation was initiated by the addition of 2 µl 10× T4 RNA ligase reaction buffer (NEB), 0.2 µl 100 mM ATP (NEB), 9 µl 50% PEG 8000 (NEB) and 0.5 µl T4 RNA ligase 1 high concentration (NEB), and the reactions were incubated overnight at room temperature. The reaction was size selected using 20 µl SPRI beads and 13.33 µl isopropanol (final concentration of 25%). The resulting cDNA was amplified in a 50 µl quantitative PCR using Illumina-compatible i5/i7 indexed primer pairs and NEBNext Ultra II Q5 (NEB) according to the manufacturer instructions until the libraries appeared to amplify linearly. The resulting libraries were purified using 1.5 volumes of SPRI beads, then sequenced using a NovaSeqX 10B XP 2 × 150bp flow cell (Illumina) at the VBCF Next Generation Sequencing Facility.

#### Analysis of short-read sequencing data (read truncation experiment)

Complete reproduction of all data analysis from the raw sequencing data to the final plots can be performed by executing the Snakemake pipeline (https://github.com/rfara/spliceosome_disassembly). In brief, all read pairs were trimmed of adapter and polyA sequences using cutadapt (v4.9) and UMI sequences were extracted using UMI-tools (v1.1.5) pre-mapped against the full human rDNA repeat sequence and GENCODE (v44) basic snRNA sequences using bowtie2 (v2.5.4). Unmapped reads pairs were mapped using STAR (v2.7.11b) against the GRCh38 primary assembly and GENCODE (v44) basic annotation. Uniquely mapping reads were deduplicated using umitools (v1.1.5). Introns and exons were defined using MANE Select transcripts from GENCODE (v44). Branchpoint locations were obtained from publicly available CoLa-seq data^75^, and the highest scoring branchpoint per intron was used for analysis. Read pairs for which the 3′ end mapped to the 3′ SS were considered to be derived from excised introns.

#### Preparation of long-read sequencing libraries (deletion rate experiment)

Of RNA, 5.5 µl was poly-adenylated by addition of 7 µl of the poly-adenylation reaction mix (4 µl 5X SuperScript IV RT buffer (Invitrogen), 1 µl 10 mM ATP, 1 µl 100 mM dithiothreitol, 0.5 µl RNasin (Promega) and 0.5 µl *E. coli* polyA polymerase (NEB)) and incubation at 37 °C for 20 min. To begin primer annealing, 1 µl of 25 mM dNTPs and 1 µl of 10 µM barcoded reverse transcription primer were added to the reaction, and the reaction was heated to 65 °C for 3 min. The reaction was cooled to 42 °C, and reverse transcription was initiated by the addition of 5.5 µl of the pre-heated reverse transcription mix (4 µl 5 M betaine, 0.5 µl 40 µM template switching oligo, 0.5 µl 200 mM MgCl_2_ and 0.5 µl SuperScript IV (Invitrogen)). The reaction was incubated at 42 °C for 1 h, then purified using homemade SPRI beads at a ratio of 1.8X. Library amplification quantitative PCR was performed using PCR mix (NEBNext Ultra II Q5 Master Mix, 1X EvaGreen (Biotium) and 500 mM partial P5 and P7 primers) with a long extension time (98 °C for 10 s, 69 °C for 20 s and 72 °C for 3 min 30 s). Samples were removed when linear amplification was observed and subsequently purified with 1.5X SPRI beads. The resulting libraries were quantified using the Qubit HS DNA kit, and the distribution of fragment sizes was quantified using the Fragment Analyzer. The libraries were sequenced using a Nanopore R10.4.1 Flow Cell.

#### Analysis of long-read sequencing data (deletion rate experiment)

The raw Nanopore data were base called using Dorado (v0.9). The libraries were demultiplexed and trimmed of adapter sequences and polyA sequences using cutadapt (v5.0). The reads were then mapped sequentially with minimap2 (v2.29)^76^ to rRNA sequences, snRNA sequences, spliced transcriptomic sequences and the genome, with the unmapped reads from mapping to each reference being passed to the next mapping. All reference sequences were derived from the hg38 human genome and the GENCODE (v44) annotation.

Branchpoint locations were annotated by CoLa-seq^75^. Deletion positions within reads were identified, with the value of a deletion being divided by the length of the deletion, and metaprofiles of the deletions were calculated relative to these branchpoint positions by summing the deletions at every position and dividing by the number of input reads. For the proportion of spliced transcripts to intronic reads, the total number of transcriptomic mapping reads and intron mapping reads was used.

### DIS release assay

#### Sample preparation

Of nuclear extract from K562 cells expressing either wild-type DHX35 or mutant DHX35(K83A), 3 ml was applied to 500 µl GFP Trap bead slurry (Chromotek) equilibrated in wash buffer (20 mM HEPES-KOH pH 7.9, 100 mM KCl, 0.5 mM TCEP, 2 mM MgCl_2_, 0.05% v/v Igepal CA-630 and 8% v/v glycerol). The beads were incubated for 3 h at 4 °C with gentle mixing. The beads were then washed five times with 3 ml wash buffer, transferred to fresh tubes and washed five more times with 1.5 ml detergent-free wash buffer (20 mM HEPES-KOH pH 7.9, 100 mM KCl, 0.5 mM TCEP, 2 mM MgCl_2_ and 8% glycerol). A small volume of both wild-type and mutant DHX35 bead samples was removed, incubated with 180 µl detergent-free wash buffer supplemented with 0.1 µg ml^−1^ 3C protease overnight at 4 °C to elute the bound DIS complexes that represented an assay input. The rest of the beads of the wild-type and mutant DHX35 samples were both distributed across six fresh tubes each, resulting in three technical replicates per sample. After removing residual wash buffer, 120 µl detergent-free wash buffer was added to each tube and incubated for 15 min at 37 °C to allow temperature equilibration, which may cause nonspecific dissociation of various proteins from the beads. The supernatant was then removed and 120 µl detergent-free wash buffer supplemented with 20 mM MgCl_2_ and either 10 mM ATP or 10 mM ADP was added to three tubes each of both the wild-type and mutant DHX35 samples. The assays were incubated for 30 min at 37 °C shaking at 800 rpm. The samples were then spun down, and the supernatants containing the proteins released from the beads were collected, run for 3 min at 180 V on a Novex Tris-glycine 4–12% 1.0 mm WedgeWell gel (Thermo Fisher Scientific) and submitted to the Vienna BioCenter Mass Spectrometry Facility for in-gel tryptic digestion and LC–MS/MS analysis.

#### Release assay analysis

The resulting raw MS data were processed for protein identification and label-free quantification. We found that one replicate of the DHX35(K83A) ADP sample had not been injected correctly for LC–MS/MS, leaving two instead of three technical replicates for this condition. To identify the proteins that are released from the beads upon ATP treatment, protein abundances in each ATP sample were compared with protein abundances in the corresponding ADP samples. The samples of wild-type DHX35 and DHX35(K83A) were compared separately to account for potential differences in the starting protein amounts on the beads. Proteins were considered significantly enriched based on statistical tests (moderated *t*-test in limma, *P* < 0.05) and an arbitrary log_2_ fold-change cut-off of 1 (twofold enrichment over control on a linear scale). Data were visualized in GraphPad Prism 10.

Release assays were also analysed by western blot using standard protocols to validate that DHX35 that could not be detected by MS was not non-specifically released from the beads. Input samples as well as the released protein samples form a single replicate of wild-type DHX35 and DHX35(K83A) each were probed with the antibodies to the spliceosomal proteins DHX35 (CAS HPA062700, Sigma; dilution 1:250), WDR83 (CAS PA-60143, Invitrogen; diluted 1:500), BRR2 (CAS AB241589, Abcam; dilution 1:1,000), PRP19 (CAS AB228694, Abcam; dilution 1:1,000) and CWC15 (CAS 25293-1-AP, Proteintech; dilution 1:1,500). The primary antibodies were incubated with the milk-blocked membranes overnight at 4 °C. A horseradish peroxidase-coupled goat anti-rabbit secondary antibody (CAS 31466, Invitrogen; dilution 1:10,000) was applied to each membrane before developing with Pierce ECL reagent (Thermo Fisher Scientific).

### Reporting summary

Further information on research design is available in the Nature Portfolio Reporting Summary linked to this article.

## Online content

Any methods, additional references, Nature Portfolio reporting summaries, source data, extended data, supplementary information, acknowledgements, peer review information; details of author contributions and competing interests; and statements of data and code availability are available at https://doi.org/10.1038/s41586-026-11101-6.

## Supplementary information

Supplementary Information Supplementary Figs. 1–8.

Reporting Summary

Supplementary Tables Supplementary Tables 1–6.

Peer Review File

## Data Availability

Three-dimensional cryo-EM composite density maps of the human ILS^AQR^ and the human DIS have been deposited into the Electron Microscopy Data Bank under the accession numbers EMD-54493 and EMD-54494, respectively. The individual maps of the ILS^AQR^ and the DIS have been deposited under the accession numbers EMD-54467–EMD-54477 and EMD-54457–EMD-54466, respectively. The cryo-EM datasets have been deposited in the Electron Microscopy Public Image Archive (EMPIAR) under accession codes EMPIAR-14002 (ILS^AQR^) and EMPIAR-14001 (DIS). The coordinate files of the human ILS^AQR^, human DIS and the revised *S. pombe* B*^D^ spliceosome have been deposited into the PDB under the accession numbers 9S2E, 9S2F and 9S2G, respectively. The MS data were deposited to the PRIDE database under the accession numbers PXD078004 and PXD081218. The RNA sequencing data were deposited to the Gene Expression Omnibus repository under the accession number GSE329374. The pipeline for RNA sequencing data analysis can be found on GitHub (https://github.com/rfara/spliceosome_disassembly).
