# Supplementary material for: Mechanism of spliceosome termination
Source: Nature. 2026 Oct 7;659(8139):248–55. doi: 10.1038/s41586-026-11101-6 (PMC13736545; doi:10.1038/s41586-026-11101-6)
Supplement: Supplementary file 1 [file 41586_2026_11101_MOESM1_ESM.pdf]

nature portfolio

https://doi.org/10.1038/s41586-026-11101-6

Supplementary information

Mechanism of spliceosome termination

In the format provided by the authors and unedited

SUPPLEMENTARY INFORMATION

Mechanism of spliceosome termination

Vytaute Boreikaite[1], Rupert Faraway[1,2,3], Matthias K. Vorländer[1], Alexander W. Phillips[1], Leonie Opitz[1], Moritz Wanke[2,3], George Yakoub[4], Gerald Raffl[2,3,5], Laura Fin[1], Román González-Pieto[6,7], Martijn S. Luijsterburg[4], Stefan L. Ameres[2,3], and Clemens Plaschka[1,*]

[1]Research Institute of Molecular Pathology (IMP), Vienna BioCenter (VBC), Campus-Vienna-Biocenter 1, 1030, Vienna, Austria.

[2]Max Perutz Labs, Vienna BioCenter (VBC), Dr.-Bohr-Gasse 9, A-1030, Vienna, Austria

[3]University of Vienna, Max Perutz Labs, Department of Biochemistry and Cell Biology, Dr.-Bohr-Gasse 9, A-1030 Vienna, Austria

[4]Department of Human Genetics, Leiden University Medical Center, Leiden, The Netherlands

[5]Vienna BioCenter PhD Program, Doctoral School of the University of Vienna and Medical University of Vienna, 1030, Vienna, Austria

[6]Andalusian Centre for Regenerative Medicine and Molecular Biology (CABIMER), Universidad de Sevilla-CSIC-Universidad Pablo de Olavide-Junta de Andalucía, Sevilla, Spain

[7]Departament of Cell Biology, Faculty of Biology, University of Sevilla, Sevilla, Spain

**Correspondence to: clemens.plaschka@imp.ac.at*

SUPPLEMENTARY TEXT

Supplementary Text 1 | ILS^{AQR} represents a distinct state of the ILS.

The ILS^{AQR} described here is a distinct state in the spliceosome termination pathway compared to previously determined structures of ILS complexes from several species[2,3,5]. We here compare the human ILS^{AQR} to the human ILS″ structure[2], which is the most likely to precede the ILS^{AQR} and whose final coordinate model is the most complete among previously published human ILS complexes. We observe three major differences: First, the ILS^{AQR} contains the novel termination factor YJU2B, which displaces a CDC5L α-helix (residues 357-382) from the combined PRP8-ESS2-SNW1 interface in ILS″. Second, in the ILS^{AQR}, the C19L1 metallophosphatase domain is repositioned owing to interactions between YJU2B with SYF1, which anchors C19L1. Third, the ILS^{AQR} contains unambiguous intron RNA density bound to AQR and PPIE, which is absent in the ILS″ (Extended Data Fig. 4e). We, therefore, observe changes to the existing protein interfaces, that go beyond the isolated gain of a subunit and indicate that the ILS^{AQR} does not result from an improved resolution of an earlier ILS complex structure, but that the ILS^{AQR} represents a distinct ILS state.

Supplementary Text 2 | GCFC2 and PAXBP1 are paralogous spliceosome termination factors.

Spliceosome termination factors GCFC2 and PAXBP1 are metazoan-specific paralogs. An α-helix of either protein can interact with an α-helix of the termination factor TFIP11, forming an anti-parallel coiled-coil and positioning both proteins on the surface of the ILS (here and refs. 2 and 3). The dimers of TFIP11 with either GCFC2 or PAXBP1 thereby recognize exterior features of the ILS and deliver the RNA helicase DHX15 to the spliceosome for disassembly[2]. Although both GCFC2 or PAXBP1 are detected by mass spectrometry in the

ILS^{AQR}, owing to the higher abundance of GCFC2, we refer to GCFC2, rather than PAXBP1, as a component of the ILS^{AQR} complex throughout this paper. We note that we could not discriminate the two paralogs in the cryo-EM densities of the ILS^{AQR} complex, owing to the low local resolution of the PAXBP1/GCFC2 region. The human ILS″ (ref.[2]) primarily contained PAXBP1, while the B*^{D} complexes from *S. pombe* and *C. thermophilum* contained only one ortholog in each species[5,23]. The specific identity of the paralog does, therefore, not appear to distinguish regular spliceosome disassembly intermediates from aberrant SQC complexes. Whether GCFC2 and PAXBP1 serve different functions in different spliceosome complexes remains to be determined.

SUPPLEMENTARY DATA TABLES

Supplementary Data Table 1 | Mass spectrometry data of the ILS disassembly assay in total nematode extract.

Abundances of proteins that remain associated with the bead-bound nematode *C. elegans* ILS in the presence or absence of ATP treatment are shown.

Supplementary Data Table 2 | Mass spectrometry data of the IPs from human cells.

Protein abundances in anti-GFP IPs using as baits wild-type DHX35, YJU2B, DBR1 or mutant DHX35 K83A are shown. Wild-type extract as a negative control is shown for each experiment. Protein abundances in wild-type DHX35 and DHX35 K83A IPs were compared in the same experiment. The DBR1-GFP pulldown was performed in triplicates, allowing to derive p-values of protein enrichment. Data from a single experiment is shown for the rest of the bait proteins, but the results of these IPs were reproduced at least twice independently for each bait.

Supplementary Data Table 3 | Cryo-EM data collection and model refinement statistics.

Supplementary Data Table 4 | Mass spectrometry data of the proximity labeling experiments in human cells.

Biotin proximity labelling experiments of TurboID-tagged DBR1, C19L1, YJU2B, and LENG1 were performed in technical triplicates in parallel with a TurboID-tagged nuclear GFP control.

Supplementary Data Table 5 | Mass spectrometry data of the human DIS release assay.

Abundances of proteins that were released upon incubation with either ATP or ADP from the bead-immobilized human DIS containing either wild-type or catalytic mutant DHX35 were analyzed in technical triplicates. Some proteins were absent from at least one sample, most often from the ADP-treated samples. In these cases, to calculate a fold change of ATP over ADP samples, the abundance of a missing protein was imputed from the lower tail of the intensity distribution in that particular sample. If the protein was absent from all samples, for example, DHX35 in the wild-type DHX35 DIS samples, no fold change was calculated, resulting in blank cells in Extended Data Table 5.

Supplementary Data Table 6 | Mass spectrometry data of the LENG1-GFP IP.

Mass spectrometry data of the LENG1-GFP pulldown from human cells. The experiment was performed in quadruplicates, and the fold-change values compare the LENG1-GFP pulldown sample with the nuclear GFP control sample.

SUPPLEMENTARY DATA FIGURES

Supplementary Data Figure 1 | Uncropped gel images of ED Fig. 1f. The dashed boxes indicate the regions displayed in ED Fig. 1f.

Supplementary Data Figure 2 | Uncropped gel image of ED Fig. 5a. The dashed box indicates the region displayed in ED Fig. 5a.

Supplementary Data Figure 3 | Uncropped gel images of ED Fig. 5g. The same gel was first imaged using fluorescence signal and then stained in Coomassie Blue. The dashed boxes indicate the regions displayed in ED Fig. 5a.

Supplementary Data Figure 4 | Uncropped gel images of ED Fig. 6c. The dashed boxes indicate the regions displayed in ED Fig. 6c.

Supplementary Data Figure 5 | Uncropped gel image of ED Fig. 6d. The dashed box indicates the region displayed in ED Fig. 6d.

Supplementary Data Figure 6 | Uncropped gel images of ED Fig. 6e. The same gel was first imaged using fluorescence signal and then stained in Coomassie Blue. The dashed boxes indicate the regions displayed in ED Fig. 6e.

Supplementary Data Figure 7 | Uncropped scans of Western blot membranes of ED Fig. 7b. The membranes had to be exposed for different times to visualize signal of all proteins. The dashed boxes indicate the regions displayed in ED Fig. 7b. Chemiluminescence signal

merged with images of molecular weight markers is shown for each scan. Each cropped membrane is numbered to identify the original membrane.

Supplementary Data Figure 8 | Uncropped scans of Western blot membranes of ED Fig. 8f. The membranes had to be exposed for different times to visualize signal of all proteins. The dashed boxes indicate the regions displayed in ED Fig. 8f. Chemiluminescence signal merged with images of molecular weight markers is shown for each scan. Each cropped membrane is numbered to identify the original membrane.

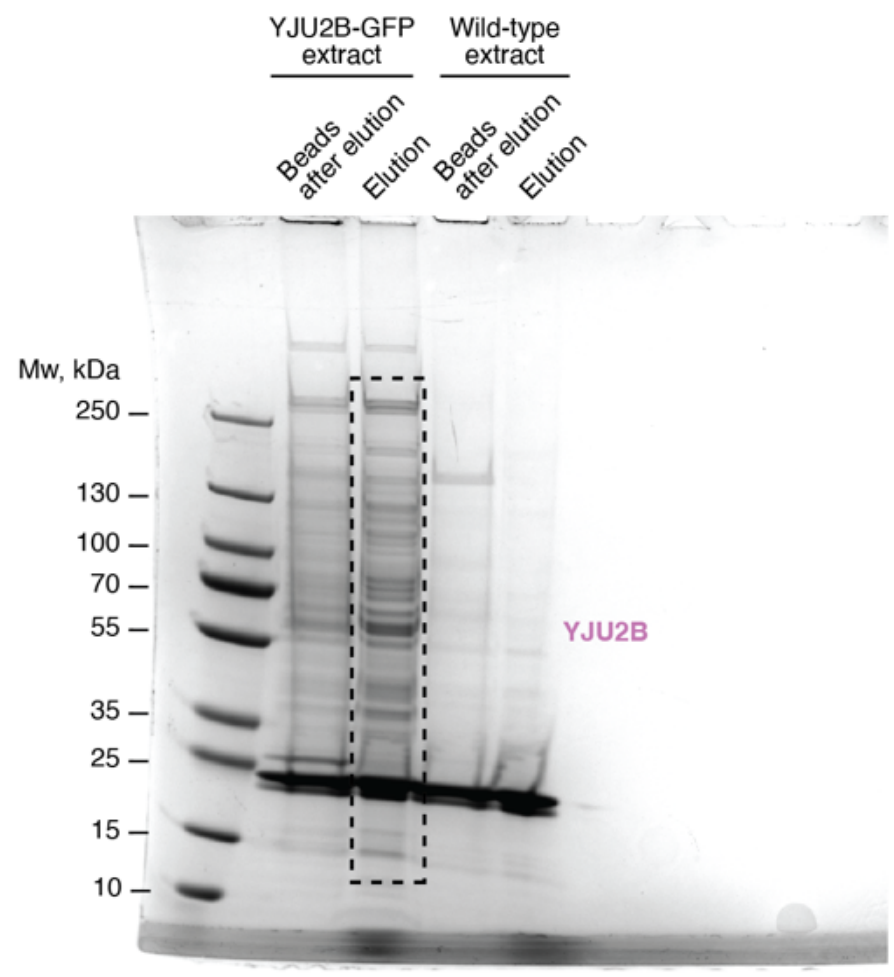

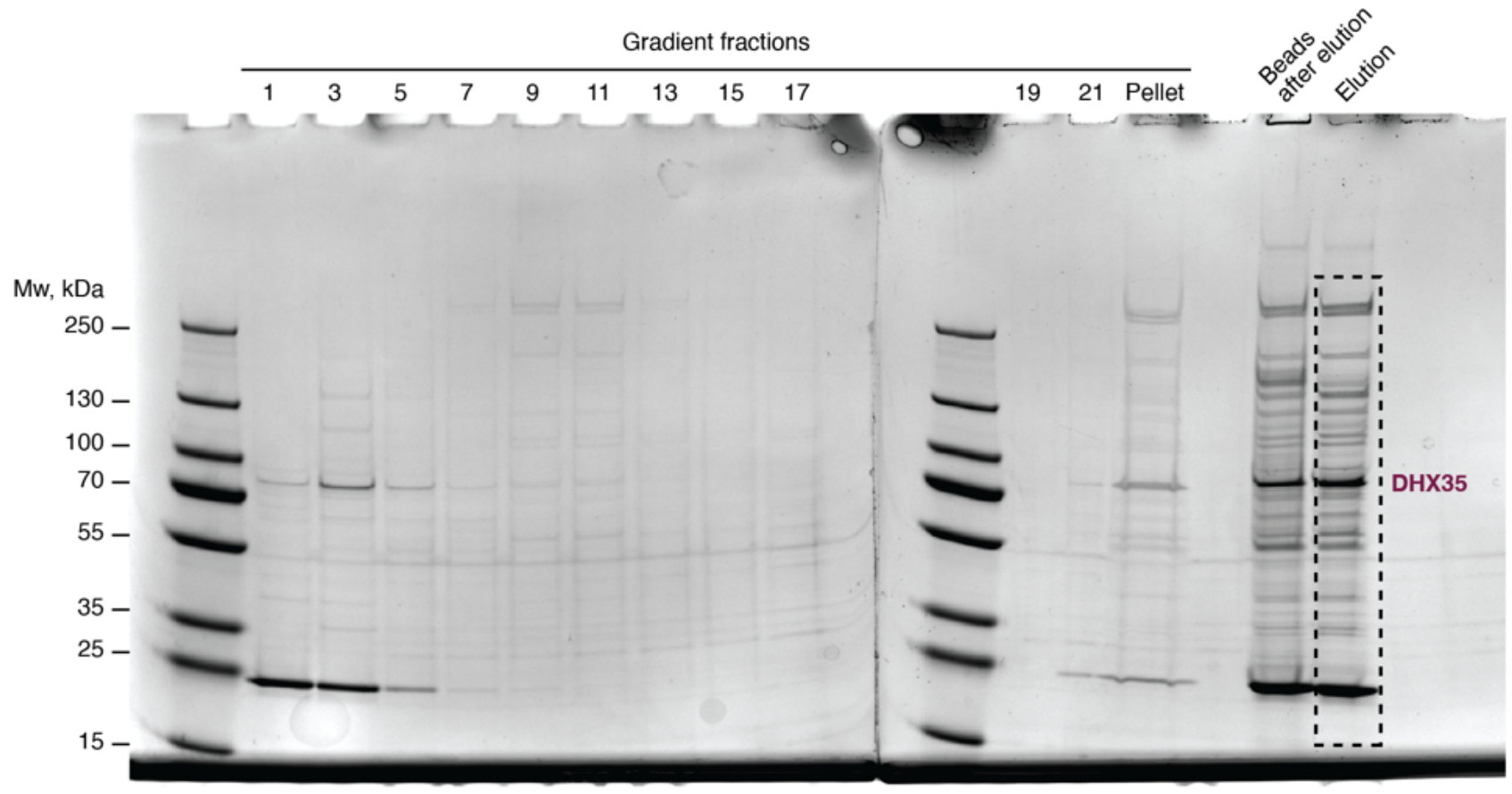

Supplementary Data Figure 1

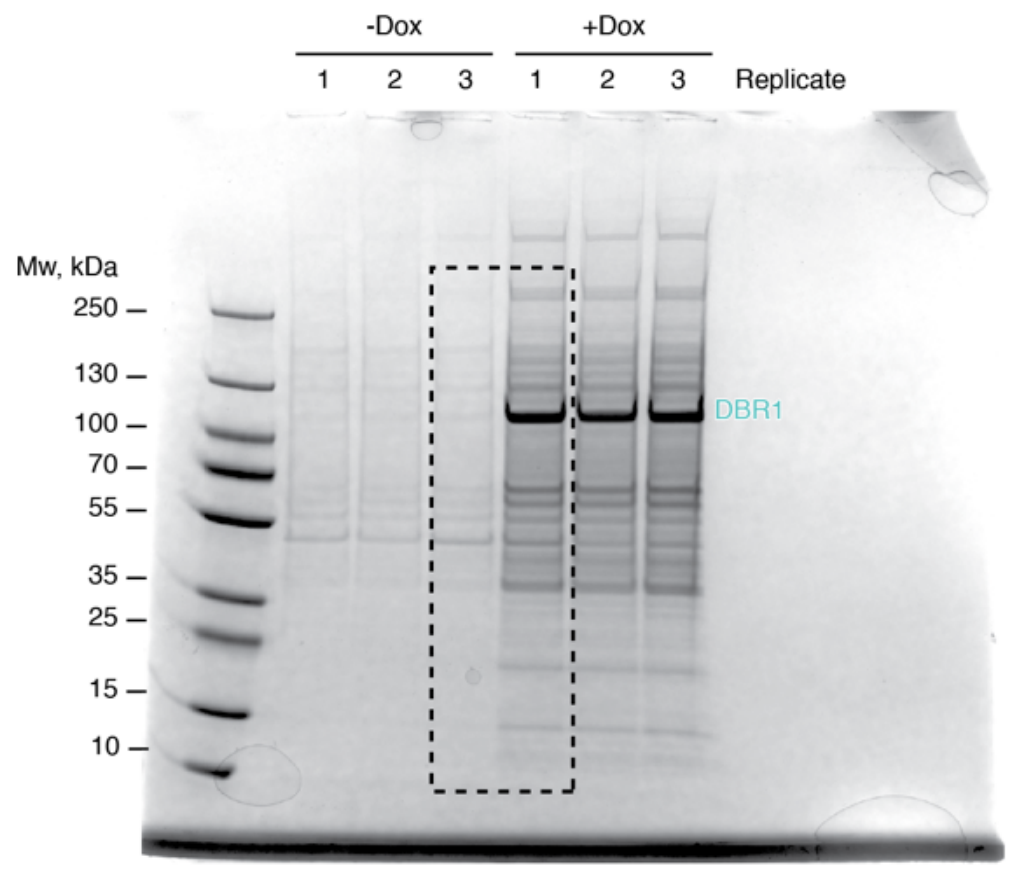

Supplementary Data Figure 2

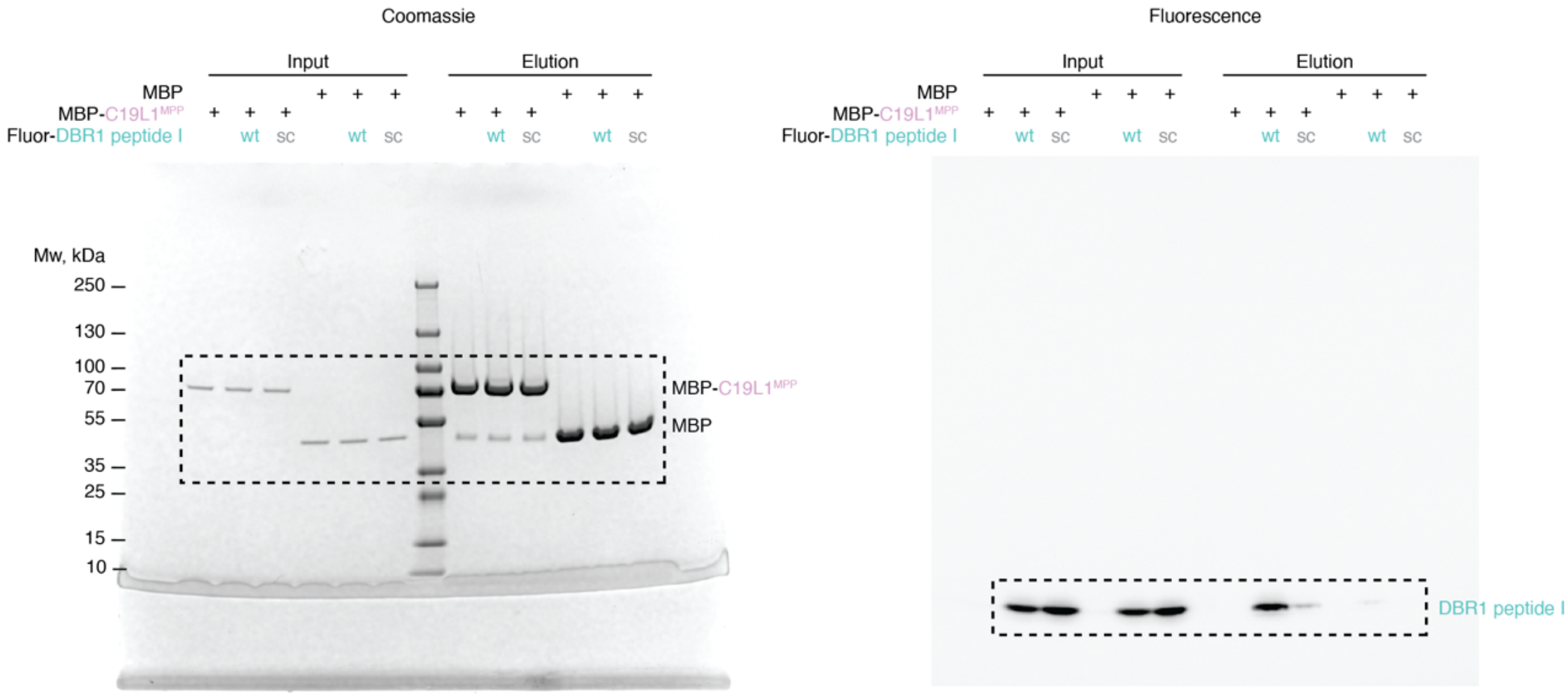

Supplementary Data Figure 3

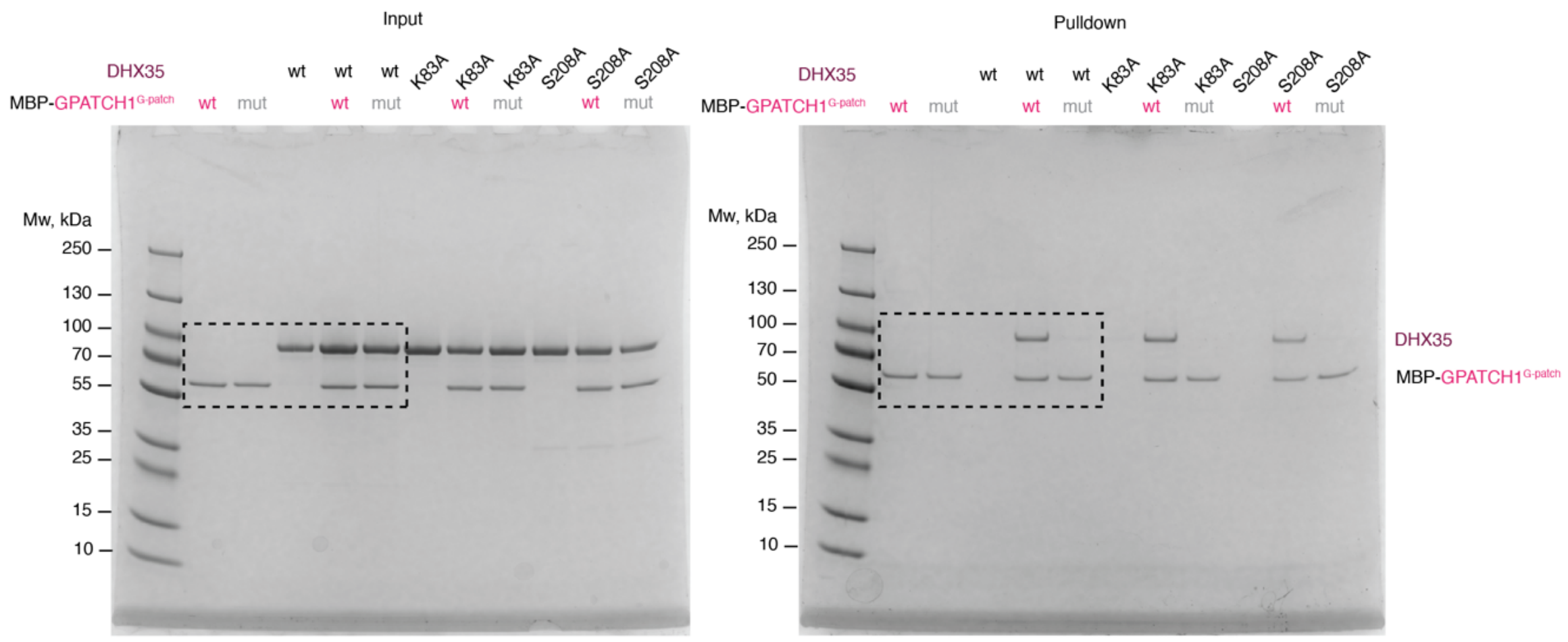

Supplementary Data Figure 4

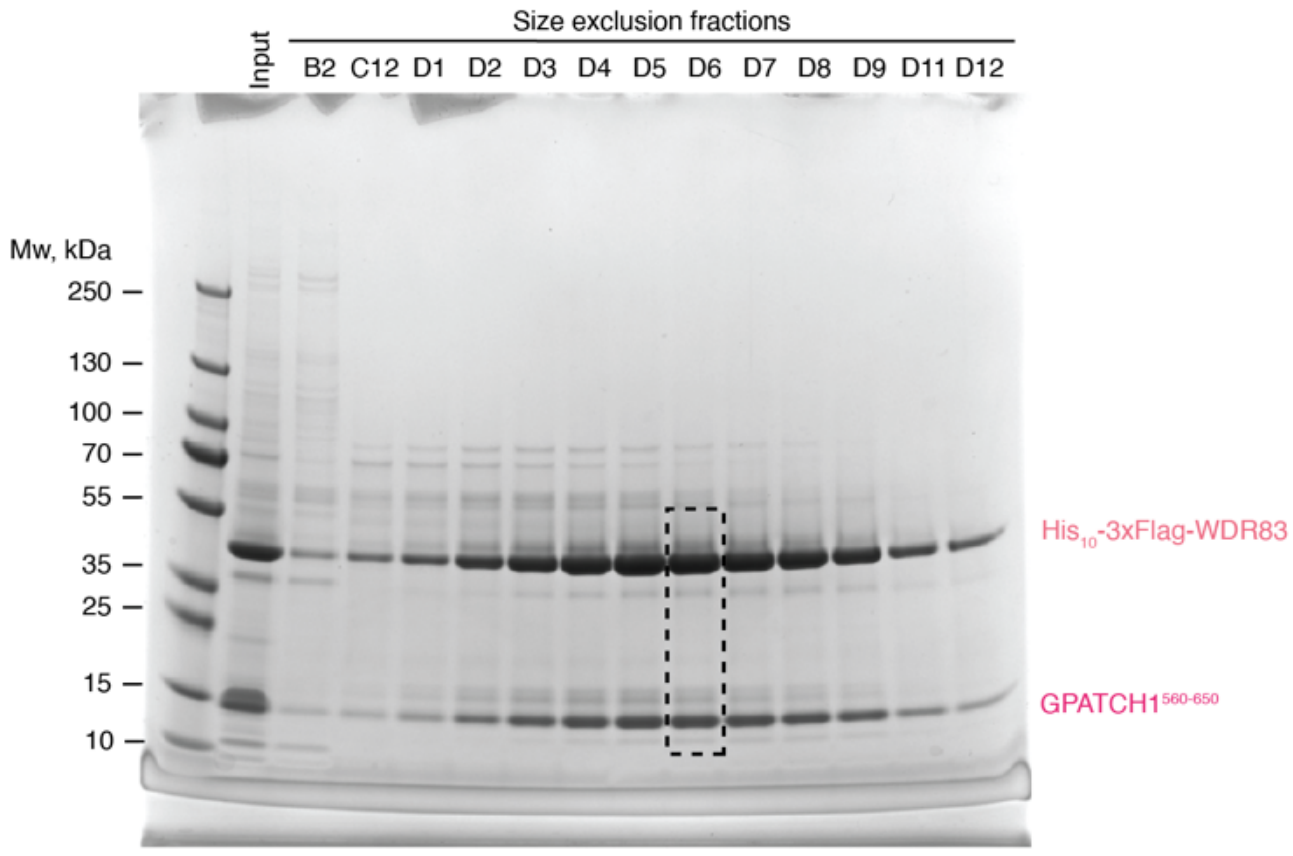

Supplementary Data Figure 5

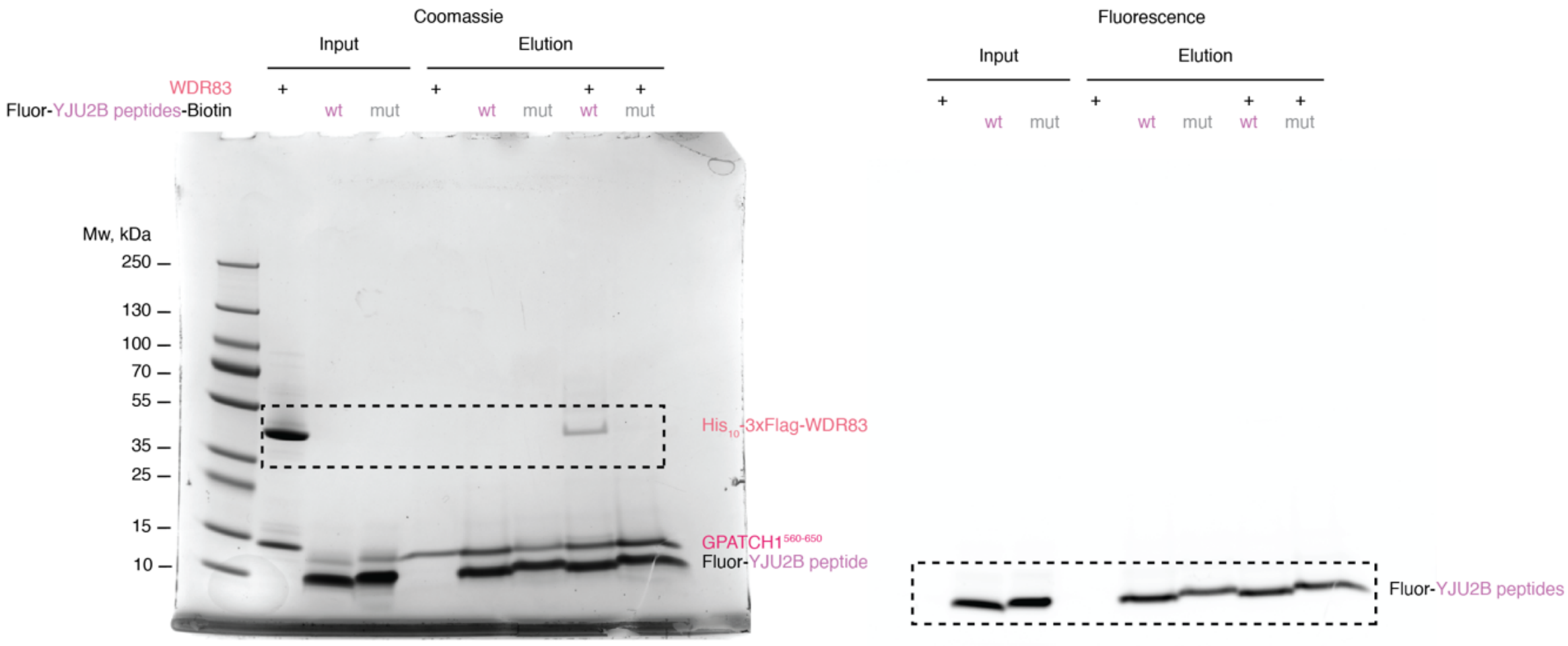

Supplementary Data Figure 6

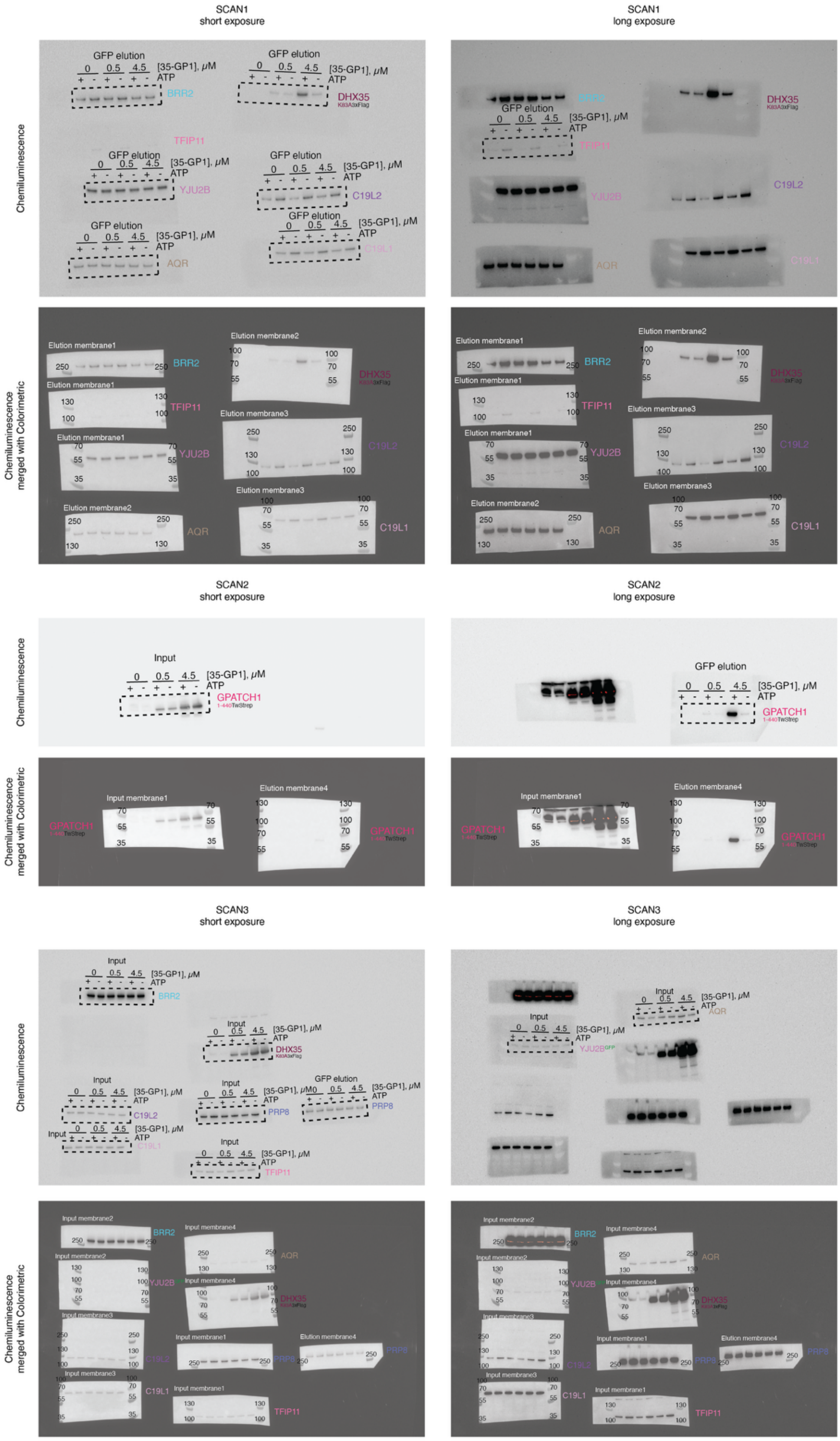

Supplementary Data Figure 7

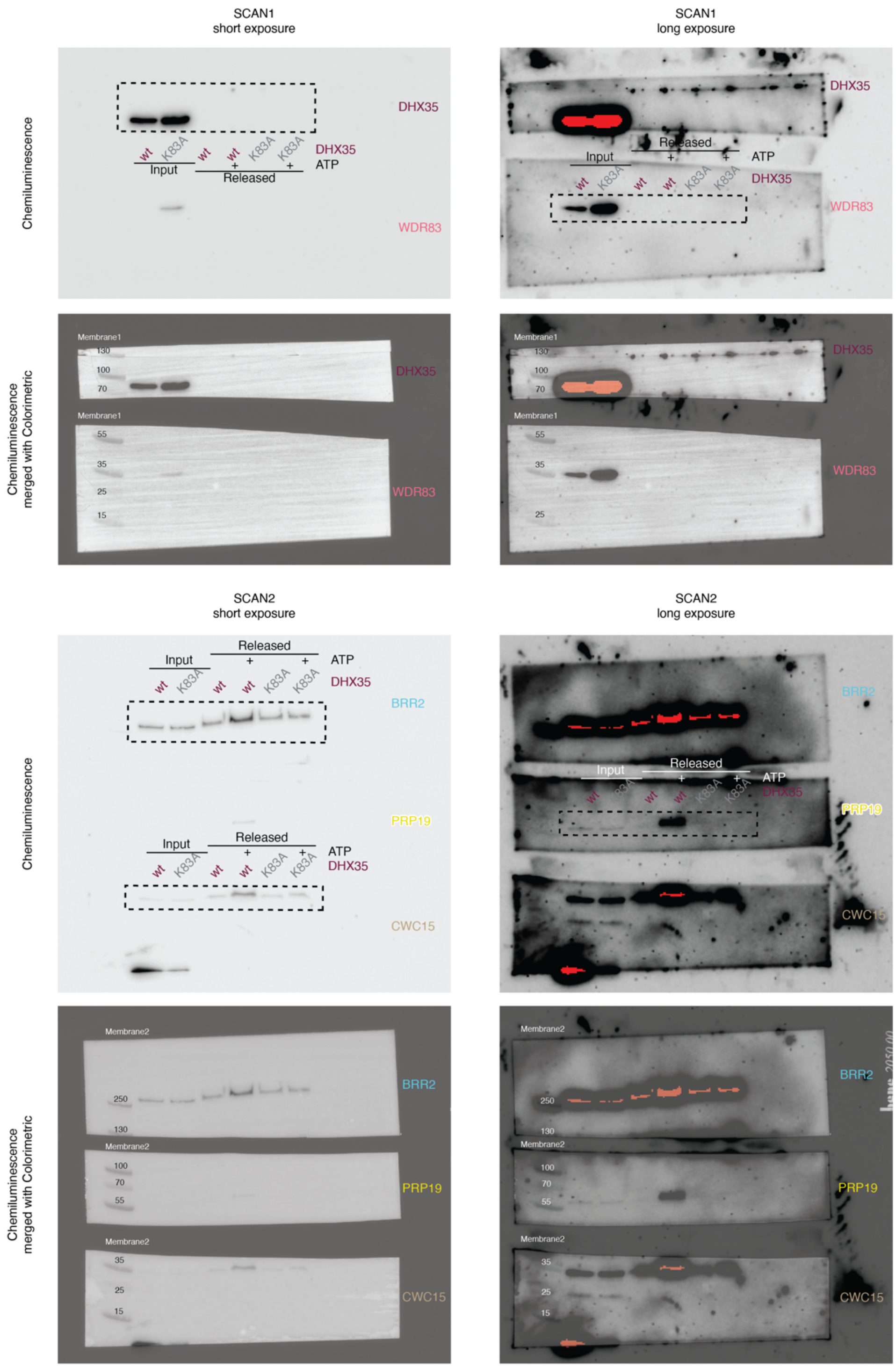

Supplementary Data Figure 8
