# Supplementary material for: Mechanism of spliceosome termination
Source: Nature. 2026 Oct 7;659(8139):248–55. doi: 10.1038/s41586-026-11101-6 (PMC13736545; doi:10.1038/s41586-026-11101-6)
Supplement: Supplementary file 2 [file 41586_2026_11101_MOESM2_ESM.pdf]

nature research

Corresponding author(s): Plaschka

Last updated by author(s): Aug 20, 2026

Reporting Summary

Nature Research wishes to improve the reproducibility of the work that we publish. This form provides structure for consistency and transparency in reporting. For further information on Nature Research policies, see our Editorial Policies and the Editorial Policy Checklist.

Statistics

For all statistical analyses, confirm that the following items are present in the figure legend, table legend, main text, or Methods section.

n/a	Confirmed	
☐	☒	The exact sample size (*n*) for each experimental group/condition, given as a discrete number and unit of measurement
☒	☐	A statement on whether measurements were taken from distinct samples or whether the same sample was measured repeatedly
☐	☒	The statistical test(s) used AND whether they are one- or two-sided<br>*Only common tests should be described solely by name; describe more complex techniques in the Methods section.*
☒	☐	A description of all covariates tested
☒	☐	A description of any assumptions or corrections, such as tests of normality and adjustment for multiple comparisons
☐	☒	A full description of the statistical parameters including central tendency (e.g. means) or other basic estimates (e.g. regression coefficient) AND variation (e.g. standard deviation) or associated estimates of uncertainty (e.g. confidence intervals)
☐	☒	For null hypothesis testing, the test statistic (e.g. *F*, *t*, *r*) with confidence intervals, effect sizes, degrees of freedom and *P* value noted<br>*Give P values as exact values whenever suitable.*
☒	☐	For Bayesian analysis, information on the choice of priors and Markov chain Monte Carlo settings
☒	☐	For hierarchical and complex designs, identification of the appropriate level for tests and full reporting of outcomes
☒	☐	Estimates of effect sizes (e.g. Cohen's *d*, Pearson's *r*), indicating how they were calculated

Our web collection on statistics for biologists contains articles on many of the points above.

Software and code

Policy information about availability of computer code

Data collection	Cryo-EM data were collected with EPU 3.8.
Data analysis	Cryo-EM data were analyzed with WARP 1.0.9, cryoSPRARC 4.6.2, RELION 5, Coot 1.1.15, Phenix 1.21.2, ISOLDE 1.9, ChimeraX 1.9. Mass spectrometry data were analyzed using limma 3.52.1, DIA-NN 2.3.2, and visualized with GraphPad Prism 10. RNA sequencing data were analyzed using cutadapt 4.9, UMI-tools 1.1.5, bowtie2 2.5.4, STAR 2.7.11b, Dorado 0.9, minimap2 2.29.

For manuscripts utilizing custom algorithms or software that are central to the research but not yet described in published literature, software must be made available to editors and reviewers. We strongly encourage code deposition in a community repository (e.g. GitHub). See the Nature Research guidelines for submitting code & software for further information.

Data

Policy information about availability of data

All manuscripts must include a data availability statement. This statement should provide the following information, where applicable:

- Accession codes, unique identifiers, or web links for publicly available datasets
- A list of figures that have associated raw data
- A description of any restrictions on data availability

Three-dimensional cryo-EM composite density maps of the human ILSAQR and the human DIS have been deposited into the EMDB under the accession numbers EMD-54493 and EMD-54494, respectively. The individual maps of the ILSAQR and the DIS have been deposited under the accession numbers EMD-54467-54477 and EMD-54457-54466, respectively. The coordinate files of the human ILSAQR, human DIS and the revised S. pombe B*D spliceosome have been deposited into the PDB under the accession numbers 9S2E, 9S2F and 9S2G, respectively. The mass spectrometry data were deposited to the PRIDE database under the accession numbers PXD078004 and PXD081218. The RNA sequencing data were deposited to the Gene Expression Omnibus repository under the accession number GSE329374. The pipeline for RNA sequencing data analysis can be found at https://github.com/rfara/spliceosome_disassembly.

Field-specific reporting

Please select the one below that is the best fit for your research. If you are not sure, read the appropriate sections before making your selection.

☒ Life sciences ☐ Behavioural & social sciences ☐ Ecological, evolutionary & environmental sciences

For a reference copy of the document with all sections, see nature.com/documents/nr-reporting-summary-flat.pdf

Life sciences study design

All studies must disclose on these points even when the disclosure is negative.

Sample size	No sample size was calculation was performed. Quantitative biochemical experiments were performed in at least three technical replicates.
Data exclusions	No data were excluded.
Replication	All replication attempts were successful. The number of replicate experiments is indicated in the figure legends of the manuscript.
Randomization	There was no allocation of test subjects for any experiments, thus randomization was not applicable to our study.
Blinding	Blinding is not applicable to the experiments reported in this manuscript, because the outcomes measured are objective and not influenced by investigators' expectations. This ensures that integrity of the data is maintained without a bias risk.

Reporting for specific materials, systems and methods

We require information from authors about some types of materials, experimental systems and methods used in many studies. Here, indicate whether each material, system or method listed is relevant to your study. If you are not sure if a list item applies to your research, read the appropriate section before selecting a response.

Materials & experimental systems

n/a	Involved in the study	
☐	☒	Antibodies
☐	☒	Eukaryotic cell lines
☒	☐	Palaeontology and archaeology
☒	☐	Animals and other organisms
☒	☐	Human research participants
☒	☐	Clinical data
☒	☐	Dual use research of concern

Methods

n/a	Involved in the study	
☒	☐	ChIP-seq
☒	☐	Flow cytometry
☒	☐	MRI-based neuroimaging

Antibodies

Antibodies used	DHX35 (CAS HPA062700, LOT R87160, Sigma), YJU2B (CAS OAAB02156, LOT SA10C624AY, Aviva SysBio), DBR1 (CAS PA5-30616, LOT ZJ4509871C, Invitrogen), PRP8 (CAS AB190347, LOT GR174279-2, Abcam), BRR2 (CAS AB241589, LOT 1032329-4, Abcam), AQR (CAS A302-546A, LOT 1, Bethyl Laboratories), TFIP11 (CAS PA5-49275, LOT WK3423919, Invitrogen), C19L1 (CAS 31700-1-AP, LOT 00155609, Proteintech), C19L2 (CAS 24872-1-AP, LOT 00021355, Proteintech), Flag (CAS F3165, LOT 0000375541, Sigma Aldrich), Strep (CAS 2-1509-001, LOT 1509-0100, iba), PRP19 (CAS AB228694, LOT 1007188-7, Abcam), CWC15 (CAS 25293-1-AP, LOT 0022338, Proteintech), HRP-coupled goat anti-rabbit secondary antibody (CAS 31466, Invitrogen).
Validation	All primary antibodies used in this study have been validated for detecting human proteins by Western blot by their respective manufacturer.

Eukaryotic cell lines

Policy information about cell lines

Cell line source(s)	Human leukemia cell line K562 (Muhar et al. 2018, Science, DOI: 10.1126/science.aao2793), human embryonic kidney cell line HEK293 Flp-In™ T-REx™ (R78007, Invitrogen), human retinal pigment epithelial cell line RPE-1 (van der Weegen et al., 2021, Nature Cell Biology, DOI: 10.1038/s41556-021-00688-9)
Authentication	All engineered cell lines were validated by DNA sequencing and Western blot analysis.
Mycoplasma contamination	All cell line were routinely tested for Mycoplasma and were tested negative.

Commonly misidentified lines<br>(See ICLAC register)	No commonly misidentified cell lines were used in this study.
