# Supplementary material for: Mechanism of spliceosome termination
Source: Nature. 2026 Oct 7;659(8139):248–55. doi: 10.1038/s41586-026-11101-6 (PMC13736545; doi:10.1038/s41586-026-11101-6)
Supplement: Supplementary file 3 [file 41586_2026_11101_MOESM4_ESM.pdf]

nature portfolio

Peer Review File

Mechanism of spliceosome termination

Corresponding Author: Dr Clemens Plaschka

Any redactions in this file are there to maintain patient confidentiality, the confidentiality of unpublished data, or to remove third-party material.

This file contains all reviewer reports in order by version, followed by all author rebuttals in order by version.

Version 0:

Reviewer comments:

Referee #1

(Remarks to the Author)
DIS Review 2025
Based on structures, computational predictions, and biochemistry, the authors have attempted to reveal how three RNA helicases AQR, DHX15, and DHX35, along with their cofactors, effect disassembly of the spliceosome. The overall model implicates AQR and DHX15 in first pulling on the branched intron to expose it to DBR1 activity. DHX35 is then suggested to translocate on the U6 snRNA to release the resulting debranched intron for disassembly. For normal spliceosomes, TFIP11 and YJU2B-C19L1/2 are proposed to coordinate initial recruitment of DHX15 and other disassembly factors. For aberrant spliceosomes, the authors propose that DHX15 and DHX35 could be recruited together and act in a coordinated manner due to different networks of interactions involving YJU2B-LENG1 and TFIP11.
Although many of these proteins had been previously implicated in disassembly both by the current group and by other groups, this study provides significant advances in understanding the mechanisms for disassembly of the human spliceosome. I commend the authors for providing a combination of integrated structural, biochemical, and functional data to support their argument. The structural data for the individual ILS and DIS complexes are compelling, and the re-interpretation of the B*D structure provides a strong link between normal and aberrant spliceosome disassembly. However, the proposal that DIS is a complex necessarily downstream of the ILSAQR and the implication that it necessitates DBR1 action for its formation, as well as the proposed model for the role of DHX35 in final disassembly require further evidence and discussion, as outlined in the numbered comments below.
ISLAQR state
1. On p. 6 the authors suggest they are the first to observe Aquarius bound to intron RNA in the ILS complex; this was certainly not unexpected and indeed it was proposed previously that Aquarius and CypE bind the intron upstream of the branch by Yigong Shi's group (Zhang et al. Cell Res, 2019), which led to the suggestion that AQR may be a chaperone for spliceosome disassembly (Fica 2020); while the AQR and RNA density is indeed more clear and convincing here, the authors should acknowledge that they are not the first to link AQR and PPIE to disassembly
2. The ILSAQR state is very reminiscent of the previously reported ILS2 state, which admittedly was very poorly defined, so the authors should clarify, based on observed composition e.g., whether these are actually different complexes, and in the process acknowledge whether they have indeed observed an entirely new state or, equally likely, actually solved the high-resolution structure of a previously observed intermediate and explained its functional role. To be clear, the mechanistic implications are a significant advance regardless, but the authors should be more careful about placing their new work in the context of previous structures and proposed models in the field, given that a role for AQR in disassembly has been at least suggested before, and their structure and biochemical work does not yet provide direct functional evidence for this role. The reviewer acknowledges that an AQR mutant could not be used to establish this role but presumably a similar assay to DIS disassembly incubating ILSAQR with ATP or ADP could provide some insights also into the ordering of the ILSAQR and DIS complexes (see also comments below)
DIS state
3. For the avoidance of doubt, a density figure of the active site for the ISLAQR and DIS complexes with the modelled RNA would be important, since in the maps there is some density continuity from the modelled U6 snRNA/5'SS helix into the Prp8 cavity that normally cradles the active site. Presumably the authors have tried extensively to classify this region, but potentially a refinement with an enforced resolution limit might illuminate how much of the intron RNA is still present in the DIS complex (see also sequencing comment below) and what has actually happened to the rest of U6 snRNA. In the absence of this, a density figure of the focussed and composite maps (at nominal and low pass filtered resolutions) would

help clarify the interpretation.
4. While the DIS and CeILS maps are consistent with the authors' interpretation that the intron is not bound in AQR in these two structures, this is not beyond doubt in my view, given the much lower resolution of the DIS AQR region and the fact that some density in the CeILS could still be consistent with individual nucleotides captured bound to the protein; do the authors have any other direct physical evidence in support of their interpretation, or does the relative distance of the modelled RecA domains in the different structures mimic more of an "open" state (e.g. compared to Upf1 structures, if available ?) – a figure for this would be useful and strengthen the interpretation in the absence of any CLIP type data from the purified complexes used for EM (note I'm not suggesting CLIP data is absolutely necessary) ; more importantly, how do they interpret the absence of RNA in the CeILS structure, would it not make more sense that RNA is still engaged in AQR in that state, but the density is simply weaker than in their Hs ILSAQR ?
5. The authors' analysis of the so-called DIS state is in my view insufficient to support the claim that the complex is simply a state where the intron has been debranched. While the high mutational rate around the BP seen in the ILSAQR is dramatically reduced in the DIS complex, that data does not only support the authors' interpretation but can also be consistent with other scenarios. The RT step can cause mutation but it can also cause stops at the BP, depending on the RT used and the library prep. The authors should at least present an analysis of the average read length from their sequencing for the two samples, and of the distribution of reads for individual introns in the ILS and DIS samples, to show convincingly that the drop in mutational rate is not due e.g. to an increase in reads that stop at or before the branch. An more complete analysis of reads observed for both flanking exons should also be presented (see below pre-mRNA comment). More importantly, the detection in both cases of high levels of exonic reads and a 2-3 fold increase in such reads in the DIS sample (EDFig. 3g, 7b) opens the possibility this could be a convolution of states where the core has been disrupted at different stages of the cycle, including at a stage before exon ligation, where the intron is present as lariat-intermediate, or even in a state where the substrate is pre-mRNA. Indeed almost 30% of the reported reads come from exons in the DIS sample and the authors themselves acknowledge in EDFig. 7d that DHX35 recruitment could occur similarly to that observed in the Spombe B*D spliceosome, where splicing may not even have proceeded past the branching step. In my view a simple primer extension experiment looking at one or two of the most abundantly observed introns/ exons in the sample could also provide more definitive proof of the exact state of the intron, asses the presence of lariat-intermediates and pre-mRNA, and strengthen the arguments regarding the state of the DIS complex, although the more detailed bioinformatic analyses above may answer some of these concerns.
6. The DHX35 disassembly assay is incomplete. The authors do not show what happens to the RNA components, which would be key to conclude disassembly state – e.g. is U6 snRNA released, as implied by the authors' argument on p. 15-16 ? what happens to the associated introns in these assays ? If the authors want to properly support their sequential model, they should actually carry out a similar assay for the ILSAQR state and compare the state of the introns (branched, debranched) in the resulting released fractions. Related to this, would DBR1 dissociate from ILSAQR in this assay ?
Sequential intron debranching and extraction model and aberrant s'some disassembly
7. Can the authors exclude the possibility that their DIS state is actually a combination of multiple complexes in the final stages of disassembly ? The occlusion of the DHX35 binding interface in earlier complexes presented in EDFig. 7e may be disrupted by the action of the specific DEAH-box proteins bound to those complexes during putative proofreading steps, making the interface accessible to engagement by DHX35. Indeed, the previous S.pombe DHX35 study proposed exactly such a mechanism at the B* stage. In my view such a possibility would actually strengthen the authors' argument for a final disassembly mechanism common throughout the splicing pathway and for both canonical and aberrant splicing. Such a possibility is also not inconsistent with a predominantly debranched state of the substrate, especially if discard would occur after chemistry at earlier stages such as C. Related to this, the MS data suggest DBR1 associates with the proposed PAXBP1, which is the GCFC2 paralog and proposed to be involved in complexes containing both DXH35 and DHX15, at least based on the C. termophilum structures (and the authors' own suggestion if Figure 5c). These observations again point to the possibility that the pathway ordering may not be as direct as the authors imply in the model Figure 5b and that DHX35 and DHX15 might co-exist in complexes containing DBR1 (the authors don't mention if any DHX35 is detected in MS of DBR1 IP). I think the authors need to show the relative abundance of all relevant disassembly factors in all of their MS data, for transparency, and discuss more explicitly what would prevent GPATCH1/DXH35 recruitment to a defective (or for that matter normal) spliceosome containing a branched intron during the catalytic stage. Is the implication that GPATCH1 would only recognise linear introns? If so, I think it is important to make this point explicitly (and provide more evidence that DHX35 does not associate with complexes containing branched RNAs).
8. The revised modelling of LENG1 in the S.pombe B*D structure is compelling, and the additional predictions and proximity labelling in the human system provide some support for LENG1 interacting with disassembly factors. However, in my view it remains unclear whether a similar B*D complex actually exists in humans. This is important to determine because in B*D both so-called ILS factors (DHX15, TFIP11) and DIS factors (DHX35,GPATCH1) are all present at the same time as the newly identified LENG1. However, in the current human study, the authors suggest that ILS factors dissociate during DHX15/AQR-mediated remodelling to allow DHX35 engagement. The existence of a similar B*D complex in humans would imply that either the pathway for disassembly of defective complexes is somewhat different, as the authors do allude to in their final discussion on p. 20-21, or that the authors' sequential model needs to be revised and one needs to potentially rethink to what extent DBR1 may be acting during the transition, on the spliceosome, or potentially after release of the intron in the case of aberrant spliceosomes that arrest following branching e.g.. Are the authors implying that defective spliceosomes that have undergone branching but cannot complete exon ligation would not be targeted by the mechanisms they describe ? The authors should at least clarify whether in their MS experiments they have observed any evidence of LENG1 associating with either ILSAQR or DIS. Related to this it isn't immediately clear why LENG1 could not associate with YJU2B ZnF in the ILS state; is it really impossible that LENG1 may be loosely associated but only engage the core if a particular intron configuration is present in the core and if CWC22 is still engaged with the spliceosome ? A figure to clarify this would be important. It is possible that in the S. pombe system the observed B*D complex can only be captured associated with the 3'-5' degradation machinery, and this might explain why LENG1 would not associate with YJU2B or DHX35 complexes at steady state. The authors should try to purify, for MS purposes, LENG1-associated complexes, to help

address the points raised above about ordering in the pathway for normal and aberrant splicing. Additionally, a more detailed analysis of the introns observed in the ILSAQR and DIS complexes might shed light on whether potentially aberrantly spliced introns elicit formation of these complexes.

Referee #2

(Remarks to the Author)
In this manuscript, Boreikaite and colleagues investigated the molecular mechanisms underlying spliceosome termination in humans, focusing on the sequential disassembly of spliceosomal complexes, intron-lariat debranching, and intron ejection. Using an integrated approach combining cryo-EM, biochemistry, RNA sequencing, and genetics, the authors identify two new termination related spliceosome states and multiple protein factors that have not been previously linked to spliceosomal termination. The first state is ILS-Aquarius (ILSAQR) which contains excised intron-lariat, termination factors, and RNA helicases DHX15 and AQR, representing an intermediate committed to disassembly. The second state is Debranched Intron Spliceosome (DIS) which contains a debranched intron tethered by partial RNA contacts and the RNA helicase DHX35 with cofactors GPATCH1 and WDR83, representing a later stage poised for intron ejection and recycling of the spliceosome. Additionally, the paper explores defective spliceosome termination, highlighting the role of YJU2B–LENG1 in recognizing aberrant pre-mRNA and guiding DHX35–GPATCH1–WDR83 to defective complexes. Overall the experiments are well designed, clearly presented, and the conclusions justified. The manuscript provides many novel insights into spliceosome termination, an understudied process of the splicing cycle, representing a significant contribution to our understanding of the molecular mechanism of pre-mRNA splicing. Below are a number of suggestions that can further strengthen the manuscript.

1. The authors identified the human termination complexes by examining the effects of incubating C. elegans ILS complexes were incubated with ATP which led to the enrichment of YJU2B and DHX35 among other proteins. They then isolated YJU2B and DHX35 containing complex from human cells. This raises the question of what would happen if human ILS complexes were incubated with ATP. Would it also lead to YJU2B and DHX35 containing complexes? This would provide strong evidence that these represent downstream complexes in the spliceosome disassembly pathway in humans.

2. Line 100. Extended Data Fig. 1e, f does not seem to be the correct figures. Please check.

3. Line 152-153. It is difficult to see how the AQR RecA2 domain shifts position before and after RNA binding. It would be helpful to include a superimposition of the two structures.

4. Line 184, "… containing a debranched intron (see below)". Please indicate the specific section below where this is discussed.

5. Line 206-211, regarding the alphafold2 multimer prediction, MPLKIP shows a higher iPTM score than SYF1 and C19L1. Doesn't this indicate that MPLKIP is a more likely interaction partner than SYF1 and C19L1? How did the authors arrive at the opposite conclusion?

6. Line 220-222: "We note that the NTR subunit SYF1 carries a highly similar motif to DBR1 peptide I (SYF1 residues 812–824), which may indicate an exchange among the two peptides at C19L1 during termination." Do the authors mean that DBR1 replaces SYF1 in binding to C19L1? It would be helpful if the authors could clarify this point and elaborate on the functional implications of this exchange.

7. Line 252: "Although we did not observe density for the late termination factors WDR83, GPATCH1 and DHX35 in the ILSAQR…" This statement implies that these factors are present in the ILSAQR complex but cannot be observed due to limitation in EM density. However, earlier biochemical data seem to indicate that these factors are not part of the complex. If this is correct, please rephrase the statement to be more accurate.

8. The authors propose that YJU2B guides ILSAQR remodeling by recruiting DHX35, GPATCH1, and WDR83. The in vitro pulldown data using purified proteins support this hypothesis. Since the authors have GFP tagged YJU2B, do these proteins associate with YJU2B by mass spectrometry or Western blot analyses of the GFP-YJU2B sample? This would provide strong evidence that these interactions indeed occur in cells.

9. In Fig. 3g-h, RNAseq is used to assess the branching status of introns in DIS. It would be helpful to include information on the quality of the RNAseq data (e.g., total number of reads) and the number of introns used for these aggregated plots. This would strengthen confidence in the comparison and support the conclusion that the intron in DIS is debranched.

Referee #3

(Remarks to the Author)
I co-reviewed this manuscript with one of the reviewers who provided the listed reports.

Referee #4

(Remarks to the Author)
"Mechanism of Spliceosome Termination" by Boreikaite et al appears to be a rigorous and fairly complete study of

spliceosome termination. The work defines two late spliceosomal states (ILSAQR and DIS) and then uses cryo-EM and proteomic and genomic approaches study the sequence of intron-lariat extraction, debranching and intron ejection. The paper is well written and the introduction does a good job laying out the problem. Using pulldown/mass-spec on GFP tagged late-stage factors revealed distinct spliceosomes. The authors technique was very good - The subsequent cryo-EM resulted in the highest quality spliceosomal structures achieved to date.

Not a lot is known about the end of the spliceosomes life cycle and this manuscript covers a lot of ground discovering and characterizing the DIS and establishing at least some cases DBR1 of acting before the final release. My overall impression was this is a very significant work that will make a contribution to our understanding of this important step in gene expression.

CONCERNS
Does the existence of two late stage spliceosomes necessarily mean that one precedes the other? Is DIS the terminal complex for all introns? Could there not be more than one way to terminate? The clip data for AQR is quite good but it shows many introns don’t have significant clip enrichment. Is it possible that heterogeneity in late complexes reflect the existence of an AQR bound and unbound fraction? I think there is evidence that AQR does help debranching because Buerer et al (ref 14) shows lariats 1) go up when AQR is knocked down, 2)have a branchpoint signature consistent with DBR1 avoidance. However they find a strong cis-effect - introns enriched for AQR CLIP signal are more susceptible to this loss DBR1. To me, this begs the question: “do all DIS complexes start as ILSAQR?”. If the answer is no, how universal is this model (i.e. what fraction go through ILSAQR)?

I believe the sequencing was only used to verify introns and lariat/linear status. It might be worthwhile to re-analyze or repeat sequencing of the RNA in complexes to demonstrate the same introns pass through both stages. If the data is too sparse for comparison, introns could be cross-checked for AQR CLIP enrichment in the introns in ILSAQR and introns in the DIS. I believe this is a relatively straightforward way to explore the generality of the model and I don’t think it would lessen the finding if the authors found alternate pathways.

Finally I would be cautious with using strong, definitive language describing conclusions that lack direct evidence. A real strength was the use of structure, biochemistry and site directed mutagenesis to return mechanistic detail like their characterization of DBR1 docking to ILSAQR. Perhaps unavoidably, not all sections enjoyed this level of experimental support. Earlier on Pg8 contains a lot of speculation based on known roles of factors in other steps of splicing cycle. I acknowledge that the many difficulties in working with and even identifying labile RNA species such as lariats may place experimental follow up beyond the scope of this work. However the more these assumptions are combined into models of concerted actions (e.g. AQR and DHX15 pulling the RNA complexes apart) the more I worry about proteins acting outside their previously characterized functions. The author may want to devise experiments to confirm mechanism or consider language that leans more towards discussion rather than the presentation of a result.

Similar concerns for statements like “YJU2B guides ILSAQR remodeling” and the last few sections.

Minor points
Line 33. I am a little uncomfortable referring to the spliceosome as a “single turnover enzyme” because as you mentioned the components can be recycled and can we say for sure that the distinct complexes (i.e. A, B, C etc) described in the literature don't remain loosely attached throughout the whole process.

The paper localizes AQR to 40 nt upstream of 3’ss. AQR binds functionally both on the 5’ss and 3’ss of the branchpoint (CLIP studies from Yeo lab and Buerer et al)

Version 1:

Reviewer comments:

Referee #1

(Remarks to the Author)
The authors have made a substantial revision that addresses most of the major concerns raised in my original review. In particular, the new RNA-sequencing analysis and human remodelling experiments strengthen the proposed relationship between the ILSAQR and DIS states, corroborates the evidence that excised introns associated with DIS are mostly debranched, and supports the role of DHX35 in late spliceosome disassembly. The additional experiments addressing LENG1 also improve the connection between canonical termination and spliceosome quality control.

The authors have dealt satisfactorily with the issues surrounding the ILSAQR structure. They now place it more carefully in the context of previous ILS structures and prior suggestions that AQR/PPIE participate in disassembly. The presence of YJU2B, rearrangement of C19L1 and, most importantly, the improved RecA domain conformation analysis strongly supports engagement of intron RNA by AQR. Together they make a reasonable case that ILSAQR represents a distinct later state rather than simply a higher-resolution version of ILS2.

The revised active-site density representations similarly make the DIS interpretation clearer. More importantly, the new sequencing experiment is a substantial improvement. By restricting the branch-point analysis to reads whose 3′ end maps to the 3′ splice site, the authors specifically analyse excised introns, and the difference in branch-point truncation between ILSAQR and DIS provides convincing evidence that this population of DIS-associated introns is predominantly debranched. The extensive overlap between introns detected in ILSAQR and DIS also provides much better evidence than in the original submission that these complexes are related states in a common termination pathway.

I also commend the authors for the new human remodelling assay. ATP treatment causes loss of ILS-specific factors, while DHX35–GPATCH1 preferentially associates with the remodelled complex, providing direct biochemical evidence for progression from ILSAQR towards a DIS-like state. The authors now also acknowledge that additional short-lived intermediates may exist, which I think is both more realistic and better supported by their own data. The evidence for DHX35-mediated disassembly has likewise been strengthened by showing that U5 and U6 snRNAs, as well as spliceosomal proteins, are depleted following ATP treatment. Although the precise RNA substrate of DHX35 remains inferred rather than directly demonstrated, the manuscript now generally distinguishes this mechanistic model from direct observation more carefully.

The new LENG1 experiments also address an important concern from the original review. The LENG1 pulldown recovers PRP8, CWC22, DHX35, GPATCH1, WDR83 and YJU2B, providing biochemical evidence that a B*D-like complex can exist in human cells. The revised discussion also more clearly explains why DHX15 and DHX35 may occupy sequential states during canonical termination but coexist in defective spliceosomes.

One important issue from my original review, however, is not in my view completely resolved. I had questioned whether the DHX35-associated DIS sample necessarily represents a single canonical post-splicing state, or whether it could contain a mixture of late disassembly and rejected spliceosomal complexes. The revised sequencing actually makes it important in my view to explicitly consider this possibility.

The authors report that approximately 30% of reads in the DIS sample are exonic, compared with approximately 7% in ILSAQR, and identify a substantial population of reads extending between intron and downstream exon sequences. They analyse these reads and find no branch-point truncation signature, which they interpret as evidence that they derive from pre-mRNAs that have not undergone branching, thereby arguing against a significant contribution from lariat-intermediate-containing complexes.

I am not convinced that this conclusion follows uniquely from the analysis. A lariat-3′-exon intermediate that had undergone branching and was subsequently debranched by DBR1 would also lack a branch-point signature while retaining continuity between the intron and downstream exon. Thus, absence of a branch-point RT-stop distinguishes branched from unbranched RNA, but as far as I can see does not distinguish an unspliced pre-mRNA from a debranched lariat intermediate. The latter would correspond to a spliceosome that had undergone the first catalytic step of branching in the B*/C complex and was subsequently rejected before exon ligation.

This population is significant: the authors analyse 68,724 intron-exon reads from 23,919 intron-exon pairs in the DHX35 purification. Indeed, the revised manuscript itself now notes that DHX35-associated RNA includes intron-exon junction reads and interprets this as evidence that DHX35 can associate with potentially aberrant substrates as well as successfully excised introns. This seems entirely compatible with my original suggestion that DHX35 may participate in a more general terminal disassembly mechanism acting on both canonical and rejected spliceosomes.

I do not think this undermines the structural characterization of the predominant DIS state, nor the conclusion that the majority of excised DIS-associated introns are debranched. The cryo-EM structure itself lacks 5′-exon density and can reasonably represent a canonical post-splicing DIS. However, I think the biochemical DHX35 purification should be treated more explicitly as potentially heterogeneous, and the sequencing does not exclude the possibility that part of the population consists of debranched, post-branching rejected complexes.

I would therefore ask the authors to address this point explicitly in the text. I do not think additional experimentation is necessary, but they should avoid implying that absence of a branchpoint signature proves that the intron-exon reads derive exclusively from pre-mRNAs that had not undergone the first catalytic step. They should acknowledge the alternative possibility of debranched post-branching intermediates and, correspondingly, that DHX35-containing DIS-like states may be reached from more than one spliceosomal intermediate.

Overall, the revision has substantially strengthened the manuscript and most of my original concerns have been addressed. The remaining issue is primarily one of interpretation and appropriately defining the heterogeneity of the DHX35-associated population. Thus, subject to clarification of this point, I support publication in Nature.

Referee #2

(Remarks to the Author)
The authors have adequately addressed all of my concerns in the revised manuscript.

Referee #3

(Remarks to the Author)
I co-reviewed this manuscript with one of the reviewers who provided the listed reports.

Referee #4

(Remarks to the Author)
The authors made changes that improved the manuscript. I still have some concern about overinterpreting the data. For example, I feel the passage “sequential passage of introns through ILS^AQR and DIS states is a universal feature of splicing” could be softened to read something like “ …. is a predominant pathway for spliceosomal disassembly” . Describing the transition as a universal feature of splicing may cause confusion because of the name DIS (Debranched Intron Spliceosome) implies that all introns are debranched while they are still in the spliceosome. Figure 3g suggests ~ 20% introns in DIS are still branched. It probably makes sense to avoid such definitive language. The cis-effect of AQR I mentioned in my original review suggests the functional requirement for AQR in facilitating debranching is not uniform across introns. The possibility that there could be other ways to expose the branchpoint to DBR1 should be discussed as a potential caveat.

Open Access This Peer Review File is licensed under a Creative Commons Attribution 4.0 International License, which permits use, sharing, adaptation, distribution and reproduction in any medium or format, as long as you give appropriate credit to the original author(s) and the source, provide a link to the Creative Commons license, and indicate if changes were made.
In cases where reviewers are anonymous, credit should be given to 'Anonymous Referee' and the source.
The images or other third party material in this Peer Review File are included in the article’s Creative Commons license, unless indicated otherwise in a credit line to the material. If material is not included in the article’s Creative Commons license and your intended use is not permitted by statutory regulation or exceeds the permitted use, you will need to obtain permission directly from the copyright holder.
To view a copy of this license, visit https://creativecommons.org/licenses/by/4.0/

List of responses to reviewer comments

“Mechanism of spliceosome termination”

(Vytaute Boreikaite, Rupert Faraway, Matthias K. Vorländer, Alexander W. Phillips, Leonie Opitz, Moritz Wanke, George Yakoub, Gerald Raffl, Laura Fin, Román González-Prieto, Martijn S. Luijsterburg, Stefan L. Ameres, and Clemens Plaschka)

Nature Manuscript 2025-08-22717

Referee expertise:
Referee #1: cryo-EM, splicing
Referee #2: cryo-EM, splicing
Referee #3: co-review with referee #2
Referee #4: splicing, biochemistry

Referee comments are in blue
Responses are in *black*

Dear Reviewers,

We thank all four reviewers for their constructive and thorough evaluation of our manuscript, which has helped us to strengthen our work. We have carefully addressed each comment through new experiments, additional analyses, and text or figure revisions. Below, we summarize the main changes, before providing a detailed point-by-point response.

The major changes are:

(a) ***A new short-read RNA sequencing experiment*** *which demonstrates, at both individual intron and transcriptome-wide resolution, that DIS-associated introns are predominantly excised and debranched. These data also show that 92.5% of the most abundantly detected ILS^{AQR}-associated introns are shared with the DIS, supporting the generality of the ILS^{AQR}-to-DIS transition.*

(b) ***A new ATP-dependent remodeling assay in human nuclear extract****, showing that ILS-specific termination factors dissociate from the ILS^{AQR} upon ATP treatment, while recombinant DHX35–GPATCH1 binds specifically to the remodeled complex. This provides further biochemical evidence for the ILS^{AQR}-to-DIS transition.*

(c) ***A native LENG1 pulldown from human cells****, which co-purifies B^{*D}-associated factors and spliceosomal proteins (including DHX35, GPATCH1, CWC22, and PRP8), providing biochemical support for a B^{*D}-like spliceosome complex in human cells.*

(d) ***Additional biochemical data, structural analyses, and improved visualizations****, including improved cryo-EM density visualizations of the ILS^{AQR} and DIS active sites and the added comparison of the RecA domain conformations of AQR across spliceosome states.*

(e) ***Revised text throughout*** *to better compare with and acknowledge prior work, to remain conservative in structural data interpretation, and to provide various text clarifications.*

Our detailed point-by-point response to each reviewer follows below.

With best regards,
Vytaute and Clemens

Referees' comments:

Referee #1 (Remarks to the Author):

DIS Review 2025

Based on structures, computational predictions, and biochemistry, the authors have attempted to reveal how three RNA helicases AQR, DHX15, and DHX35, along with their cofactors, effect disassembly of the spliceosome. The overall model implicates AQR and DHX15 in first pulling on the branched intron to expose it to DBR1 activity. DHX35 is then suggested to translocate on the U6 snRNA to release the resulting debranched intron for disassembly. For normal spliceosomes, TFIP11 and YJU2B-C19L1/2 are proposed to coordinate initial recruitment of DHX15 and other disassembly factors. For aberrant spliceosomes, the authors propose that DHX15 and DHX35 could be recruited together and act in a coordinated manner due to different networks of interactions involving YJU2B-LENG1 and TFIP11.

Although many of these proteins had been previously implicated in disassembly both by the current group and by other groups, this study provides significant advances in understanding the mechanisms for disassembly of the human spliceosome. I commend the authors for providing a combination of integrated structural, biochemical, and functional data to support their argument. The structural data for the individual ILS and DIS complexes are compelling, and the re-interpretation of the B*D structure provides a strong link between normal and aberrant spliceosome disassembly. However, the proposal that DIS is a complex necessarily downstream of the ILSAQR and the implication that it necessitates DBR1 action for its formation, as well as the proposed model for the role of DHX35 in final disassembly require further evidence and discussion, as outlined in the numbered comments below.

We thank the reviewer for their insightful comments, which we have addressed in the point-by-point rebuttal below and which have helped us to improve the manuscript.

ISLAQR state

1. On p. 6 the authors suggest they are the first to observe Aquarius bound to intron RNA in the ILS complex; this was certainly not unexpected and indeed it was proposed previously that Aquarius and CypE bind the intron upstream of the branch

by Yigong Shi's group (Zhang et al. Cell Res, 2019), which led to the suggestion that AQR may be a chaperone for spliceosome disassembly (Fica 2020); while the AQR and RNA density is indeed more clear and convincing here, the authors should acknowledge that they are not the first to link AQR and PPIE to disassembly.

We thank the reviewer for this point. We did not intend to give the impression of being the first to link AQR and PPIE (CypE) to spliceosome disassembly. We have now rephrased the main text (Lines 153 and 168-169) to more explicitly acknowledge the prior work by the Shi group (Zhang et al., Cell Res., 2019) and the review (Fica, Curr. Opin. Struct. Biol., 2020). While PPIE and AQR have indeed been proposed to bind to the intron in the human ILS (Zhang et al., Cell Res., 2019) and AQR has been suggested to play a potential role in spliceosome disassembly (Fica, Curr. Opin. Struct. Biol., 2020), the previous ILS cryo-EM densities by Zhang et al. did not enable atomic modeling of AQR, or of the nearby intron-binding complex (IBC) subunits, or of intron RNA near the IBC owing to the low local density quality. We here report unambiguous and high-quality density for AQR, the IBC, and the intron RNA at 3.9 Å resolution, enabling new insights into the binding of intron RNA to the IBC.

2. The ILSAQR state is very reminiscent of the previously reported ILS2 state, which admittedly was very poorly defined, so the authors should clarify, based on observed composition e.g., whether these are actually different complexes, and in the process acknowledge whether they have indeed observed an entirely new state or, equally likely, actually solved the high-resolution structure of a previously observed intermediate and explained its functional role. To be clear, the mechanistic implications are a significant advance regardless, but the authors should be more careful about placing their new work in the context of previous structures and proposed models in the field, given that a role for AQR in disassembly has been at least suggested before, and their structure and biochemical work does not yet provide direct functional evidence for this role. The reviewer acknowledges that an AQR mutant could not be used to establish this role but presumably a similar assay to DIS disassembly incubating ILSAQR with ATP or ADP could provide some insights also into the ordering of the ILSAQR and DIS complexes (see also comments below).

Thank you for these comments. Based on the observations described below, we conclude that the ILS^{AQR} represents a distinct state of the spliceosome compared to the previously reported ILS2 state identified in Zhang et al., Cell Res., 2019.

The previously reported human ILS2 complex in Zhang et al., Cell Res., 2019 may correspond to a partial complex of the human and C. elegans ILS″ (Vorländer et al., Nature, 2024). Specifically, the ILS2 did not contain densities for the termination factors TFIP11, PAXBP1 and C19L1, which were however identified in the ILS″ complexes. These additional factors may nevertheless have been part of the ILS2 complex sample, because ILS2 contained (i) C19L2, which forms a heterodimer with C19L1; (ii) DHX15, which is tethered to the spliceosome by its association with the G-patch domain of TFIP11; (iii) and TFIP11 itself forms a heterodimer with PAXBP1 or its paralog GCFC2. The previously reported ILS2 might thus be a lower resolution version of the ILS″ complex.

In contrast to these structures, our human ILS^{AQR} structure represents a distinct state. In the text below, we compare the human ILS^{AQR} to the human ILS″, whose structural model is more complete than ILS2 and which likely precedes the ILS^{AQR}. There are three major differences. First, the ILS^{AQR} contains the novel termination factor YJU2B, which displaces a CDC5L a-helix (residues 357-382) from the combined PRP8-ESS2-SNW1 interface in the ILS″. Second, in the ILS^{AQR}, the C19L1 metallophosphatase (MPP) domain is repositioned owing to interactions between YJU2B with SYF1, which anchors C19L1. Third, the ILS^{AQR} contains unambiguous intron RNA density bound to AQR and PPIE, which is absent in the ILS″ as described in detail through structural comparisons to the Ce ILS″ in our response to reviewer's comment #4. These comparisons show that intron RNA binding to AQR appears to be unique to ILS^{AQR}, indicating that this state does not result from an improved resolution of an earlier ILS complex but represents a distinct state. We also observe changes to the existing protein interfaces, including the enrichment of GCFC2 over PAXBP1 in the ILS^{AQR} (see mass spectrometry data in Extended Data Fig. 1g), thus going beyond the isolated gain of a subunit. These observations support that the ILS^{AQR} represents a distinct ILS state. We agree with the reviewer on the importance of being clear on how we place this structure relative to the prior ILS structures. We have rephrased the main revised manuscript text (Lines 136-137) and now added Supplementary Text 1 to explain this in more detail.

We also agree with the reviewer that probing the role of AQR in the ILS^{AQR} is technically exceedingly challenging, owing to AQR's role in earlier splicing steps, including spliceosome activation, and because of the presence of the second RNA helicase DHX15 in ILS^{AQR}, which would be activated simultaneously with AQR upon addition of ATP in biochemical assays. However, to investigate the general

placement of the ILS^{AQR} structure relative to the DIS complex, we have now carried out additional biochemical and RNA sequencing experiments described in detail in our response to the reviewer's comment #6. These results are fully consistent with our proposed ordering of ILS^{AQR} and DIS complex states and the generality of the DIS in the splicing pathway.

DIS state

3. For the avoidance of doubt, a density figure of the active site for the ISLAQR and DIS complexes with the modelled RNA would be important, since in the maps there is some density continuity from the modelled U6 snRNA/5'SS helix into the Prp8 cavity that normally cradles the active site. Presumably the authors have tried extensively to classify this region, but potentially a refinement with an enforced resolution limit might illuminate how much of the intron RNA is still present in the DIS complex (see also sequencing comment below) and what has actually happened to the rest of U6 snRNA. In the absence of this, a density figure of the focussed and composite maps (at nominal and low pass filtered resolutions) would help clarify the interpretation.

Thank you for these suggestions. We have carried out additional rounds of 3D classification, 3D variability, and focused 3D refinements using cryoSPARC with various masks on the RNA active site of the DIS complex. Despite these efforts, we did not obtain improved putative RNA densities in the DIS active site, indicating that there is a high degree of flexibility in these regions. As suggested by the reviewer, we here provide a more detailed visualization of the cryo-EM densities of the DIS active site in both unfiltered and low-pass filtered composite and active site-focused (map #2) maps of the complex (Response Fig. 1.1). We do indeed observe noisy density extending from the partial U6 snRNA-intron duplex in the DIS. This suggests that both RNAs may continue beyond the modeled region. However, these extended RNA regions apparently cannot be resolved by cryo-EM owing to their mobility. This would be consistent with our model, in which the non-modelled, and thus active site-unbound, segments of U6 snRNA and of the linearized intron RNA are flexibly anchored to the partial U6 snRNA-intron duplex in the DIS. To further experimentally validate that the lack of extended U6 snRNA density does not stem from its degradation in the DIS sample, we now also supply a Northern blot analysis of U6 snRNA from purified DIS complexes (Response Fig. 1.3). The blot shows a U6 snRNA band at the expected position of intact U6 snRNA. These data are consistent with U6 snRNA being flexibly tethered in the DIS by the partial U6-intron duplex. To

remain conservative in the final coordinate model, we have not extended our RNA models into the lower quality densities.

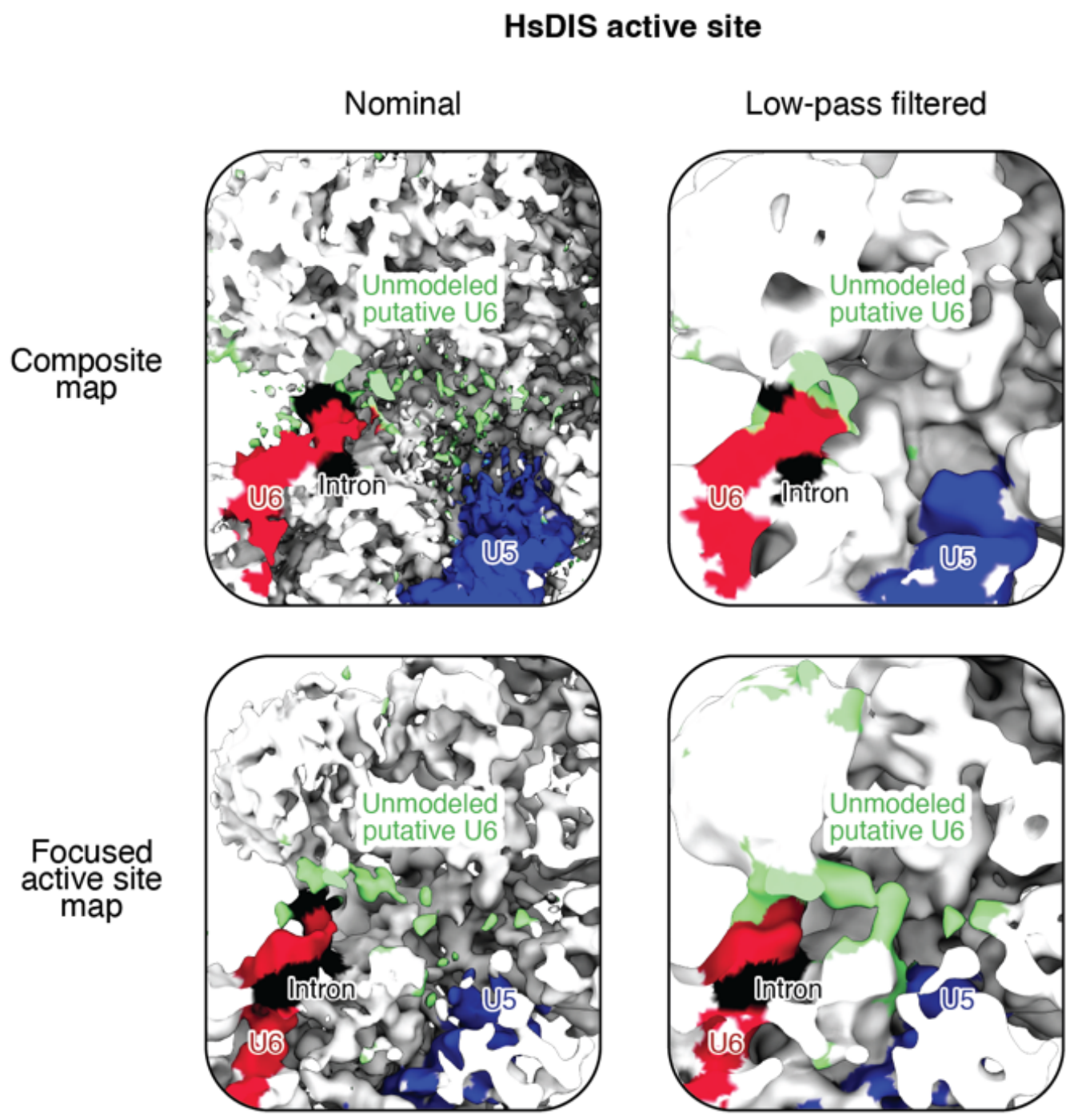

Response Fig. 1.1 | Cryo-EM densities of the DIS active site. *The low-pass filtered maps were generated in ChimeraX by applying a Gaussian filter with a standard deviation of 2 voxels. The density corresponding to the modeled protein components is colored in gray, while the density of the modeled RNA is color coded. Unmodeled putative RNA density is depicted in green, which we did not model into to remain conservative with cryo-EM density interpretation.*

In the submitted manuscript, we colored the low-pass filtered cryo-EM densities of both ILS^{AQR} and DIS by the modeled RNA elements to show that the active site cavity in the DIS complex is empty, especially when compared to the equivalent region in ILS^{AQR} (previous Extended Data Fig. 7a). To avoid ambiguity that the reviewer has pointed out and to more clearly visualize our best-quality cryo-EM densities of both the DIS and ILS^{AQR} active sites, we have now added further panels to the new Extended Data Fig. 7c, showing the respective modeled RNAs within composite cryo-EM densities of both DIS and ILS^{AQR} complexes alongside the previously provided side-by-side comparisons of low-pass filtered cryo-EM densities of the ILS^{AQR} and DIS active sites. They are colored by the modeled RNA elements, while the modeled proteins are depicted as cartoons. Together, these show that our current DIS and ILS^{AQR} RNA models show an excellent fit to the experimental cryo-

EM densities and that the active site cavity in the DIS complex is largely empty when compared to the equivalent region in ILS^{AQR} (new Extended Data Fig. 7c).

4. While the DIS and CeILS maps are consistent with the authors' interpretation that the intron is not bound in AQR in these two structures, this is not beyond doubt in my view, given the much lower resolution of the DIS AQR region and the fact that some density in the CeILS could still be consistent with individual nucleotides captured bound to the protein; do the authors have any other direct physical evidence in support of their interpretation, or does the relative distance of the modelled RecA domains in the different structures mimic more of an "open" state (e.g. compared to Upf1 structures, if available ?) – a figure for this would be useful and strengthen the interpretation in the absence of any CLIP type data from the purified complexes used for EM (note I'm not suggesting CLIP data is absolutely necessary) ; more importantly, how do they interpret the absence of RNA in the CeILS structure, would it not make more sense that RNA is still engaged in AQR in that state, but the density is simply weaker than in their Hs ILSAQR ?

We thank the reviewer for their observations. The previously reported cryo-EM map of the Ce ILS″ indeed contains noisy density in the active site of AQR. However, these blobs of noisy density do not match the position of the expected RNA path (nucleotide bases or associated phosphate backbone) within the active site of AQR, as seen in the cryo-EM density of the ILS^{AQR} and the crystal structure of the RNA-bound Upf1 (Chakrabarti et al., Mol. Cell, 2011), which is a closely related SF1 RNA helicase. Interestingly, these discontinuous densities are surrounded by basic residues in Ce ILS″ AQR (K673, R1240, K1264, K1258). One possible explanation for these density features is spontaneous crosslinking with glutaraldehyde during the gradient fixation protocol. Specifically, because these residues are available in the Ce ILS″ and not blocked by RNA, glutaraldehyde may crosslink the neighboring primary amines, as has been previously documented (Schiffner et al., PLoS Pathog., 2018; Zdanowicz et al., Sci. Adv., 2024). To investigate whether a subset of particles in the former Ce ILS″ AQR dataset nevertheless contained RNA, we have also carried out further 3D classifications around the AQR active site in the Ce ILS″ data, which, however, did not reveal any new densities. Taken together, we conclude that AQR in the Ce ILS″ is not bound to RNA, which is also supported by the "open" conformation of the AQR RecA lobes (see below and new Extended Data Fig. 4d).

We also thank the reviewer for prompting us to carefully assess the RNA-bound and RNA-unbound states of AQR in the experimental cryo-EM densities of the various

spliceosome complexes. We agree that a robust read-out, even at lower resolution, for RNA-binding in AQR is the relative orientations of the two AQR RecA domains across spliceosomes when compared to the closely related SF1-family RNA helicase Upf1. High-resolution crystal structures of human Upf1 in the presence and absence of RNA are available (Chakrabarti et al., Mol. Cell, 2011). In these crystal structures, the Upf1 RecA domains move closer when bound to RNA by a ~25° degree rotation and a center-to-center movement of the RecA2 domain of 8 Å (new Extended Data Fig. 4d, top two panels). In agreement with these Upf1 data, the RecA lobes of the highly similar RNA helicase AQR in our ILS^{AQR} structure, which is RNA-bound, are positioned in a highly similar manner (new Extended Data Fig. 4d, middle left panel). In contrast, we observe that the AQR RecA lobes in the Ce ILS″, in which AQR is not bound to RNA (see previous paragraph) are positioned further apart, similar to RNA-unbound Upf1 (new Extended Data Fig. 4d, middle right panel). In addition, the conformation of AQR in the DIS closely resembles that of AQR in the RNA-unbound Ce ILS″ (new Extended Data Fig. 4d, bottom panel) but not the RNA-bound conformation seen in the ILS^{AQR}. These findings support the notion that AQR in the DIS is not bound to RNA. This is consistent with a model in which AQR would "run off" the linear intron after debranching owing to the 5′-to-3′ polarity of the helicase. Specifically, AQR would be able to translocate on ~65 intron nucleotides (AQR binding 40 nucleotides upstream of the branch point plus ~25 nucleotides between the branch point and the 3′-splice site (Taggart et al., Nat. Struct. Mol. Biol., 2012) across the now-linear intron branch point, eventually falling off the intron at the 3′-splice site. The apparent flexibility of the intron-binding complex in the DIS would be further consistent with the lack of stable RNA engagement by AQR in contrast to the ILS^{AQR}.

To clarify this point in the manuscript, we have added the RecA lobe conformation comparison of RNA-bound and -free Upf1, the RNA-bound AQR in ILS^{AQR} and the RNA-free AQR in the Ce ILS″ and Hs DIS to Extended Data Fig. 4d, and updated the manuscript main text (Line 158-160) to the following: "In contrast to the RNA-free AQR in the preceding ILS complexes, in the ILS^{AQR} the AQR RecA2 domain shifts position to close over the bound intron-lariat RNA (Extended Data Fig. 4d)."

5. The authors' analysis of the so-called DIS state is in my view insufficient to support the claim that the complex is simply a state where the intron has been debranched. While the high mutational rate around the BP seen in the ILSAQR is dramatically reduced in the DIS complex, that data does not only support the authors' interpretation but can also be consistent with other scenarios. The RT step

can cause mutation but it can also cause stops at the BP, depending on the RT used and the library prep. The authors should at least present an analysis of the average read length from their sequencing for the two samples, and of the distribution of reads for individual introns in the ILS and DIS samples, to show convincingly that the drop in mutational rate is not due e.g. to an increase in reads that stop at or before the branch.

We thank the reviewer for this suggestion. The reviewer is correct that branched introns should also cause truncations at the branch point during reverse transcription with a certain frequency. We were, however, unable to analyze these data for truncated reads because of the RNA library preparation method in the submitted manuscript. Specifically, these short fragments were lost in the protocol because these branch point truncations did not result in non-templated nucleotide addition required for the template switching reaction (see Materials and Methods). In addition, we came to appreciate that this library preparation strategy is technically inefficient, especially because of the low amounts of input RNA we obtain from immune-precipitation and subsequent gradient-purification of native spliceosomes. As a result, the read coverage was insufficient to draw conclusions from the deletion rates of individual introns as suggested by the reviewer. While our previous library preparation strategy did not allow for this kind of analysis, we now provide a new RNA sequencing experiment for the revision, where we established an improved library preparation method, inspired by spliceosome iCLIP (Briese et al., Nat. Struct. Mol. Biol., 2019), and sequenced the resulting libraries at much greater depth.

To do this, we extracted RNA from gradient-purified ILS^{AQR} and DIS complexes starting from ~24 L of human cell culture each. We developed a library preparation method inspired by spliceosome iCLIP (Briese et al., NSMB, 2019; Materials and Methods in the revised manuscript), in which RNA was initially polyadenylated on the 3′-end and then chemically fragmented. While the sites of fragmentation are random, the reads coming from excised introns will be polyadenylated specifically at 3′-splice sites. We then performed reverse transcription from this poly-adenylated tail and ligated a DNA adapter to the resulting cDNA. This approach allowed us to capture all cDNAs, independent of whether the reaction truncated at a branch point. We then performed short-read RNA sequencing. For the subsequent data analysis, we selected the reads corresponding to excised introns which are the reads beginning at the 3′-splice site. We assessed the intron branching status of these reads from either ILS^{AQR} or DIS complexes by determining how frequently these reads terminate at the annotated branch point (as identified in Zeng et al., Mol. Cell, 2022).

The improved sequencing read coverage in the new experiment allowed us to assess the intron branching status based on the reads of individual introns, as suggested by the reviewer. To visualize the experiment quality with an example, we show the read coverage plot for three adjacent introns within the HNRNPA1 transcript (new Fig. 3f). Here, we observe the expected sharp drop in read coverage at the predominant branch point when these introns are associated with the purified ILS^{AQR}, but not when the same introns are associated with the purified DIS complex (new Fig. 3f). This shows that reverse transcription does indeed frequently terminate at branch points of ILS^{AQR}-associated introns, where we know that all introns should be branched. In contrast, the reads from the DIS span across the branch point, suggesting that its associated introns are almost entirely debranched (new Fig. 3f). To investigate whether th ese conclusions hold true transcriptome wide, we plotted the read coverage of all excised introns detected in both samples (89,807 introns; new Fig. 3g). The resulting metaplot reveals that the excised introns in the DIS show significantly more read-through past the branch point than the excised ILS^{AQR} introns, consistent with excised DIS introns being largely debranched. In this analysis we include only excised intron reads that start at the annotated 3′-splice site (new Fig. 3h).

While some DIS introns show a reduction in coverage at the branch point, this is to a significantly lower extent compared to the ILS^{AQR} (new Fig. 3g), consistent with the reduced deletion rate observed for DIS introns in our previous RNA sequencing experiment (new Extended Data Fig. 7e). Since ILS-specific proteins are absent from gradient-purified DIS (Extended Data Fig. 1g), this signal is unlikely to derive from co-purified ILS complexes. Rather, it may be that a small number of contaminating intron-lariats, which have not yet been debranched, comigrate with the DIS in sucrose gradients (Makarova & Makarov, PLoS ONE, 2015), or alternatively that these intron-lariats are debranched after release from the spliceosome. This would further suggest that intron-lariat debranching is not a strict biochemical requirement for the ILS^{AQR}-to-DIS transition, which we now also mention in the revised manuscript (Lines 328-331 and lines 867-868 in the legend of Fig. 5b). Taken together, our data demonstrate that the vast majority of DIS-associated introns are debranched.

We have moved the plots of the previous deletion rate-based experiment from Fig. 3 to Extended Data Figs. 7 panels d-f. The previous experiment complements the new and improved RNA sequencing assay, strengthening our conclusions regarding the

presence of excised introns and their branching status by using two orthogonal approaches. We have replaced these plots with the new experiment in the main text Fig. 3 panels f-i.

An more complete analysis of reads observed for both flanking exons should also be presented (see below pre-mRNA comment). More importantly, the detection in both cases of high levels of exonic reads and a 2-3 fold increase in such reads in the DIS sample (EDFig. 3g, 7b) opens the possibility this could be a convolution of states where the core has been disrupted at different stages of the cycle, including at a stage before exon ligation, where the intron is present as lariat-intermediate, or even in a state where the substrate is pre-mRNA. Indeed almost 30% of the reported reads come from exons in the DIS sample and the authors themselves acknowledge in EDFig. 7d that DHX35 recruitment could occur similarly to that observed in the Spombe B*D spliceosome, where splicing may not even have proceeded past the branching step. In my view a simple primer extension experiment looking at one or two of the most abundantly observed introns/ exons in the sample could also provide more definitive proof of the exact state of the intron, asses the presence of lariat-intermediates and pre-mRNA, and strengthen the arguments regarding the state of the DIS complex, although the more detailed bioinformatic analyses above may answer some of these concerns.

We thank the reviewer for raising this point. We wish to note that our analysis of the branching status considers only the intron reads coming from excised introns, meaning those reads that begin at the 3′-splice site. Thus, the presence of exonic reads in the two samples does not affect the conclusions of the branching analysis presented above. We agree with the reviewer that the exonic reads, ~7% of genic ILS^{AQR} reads and ~30% of genic DIS reads seen in both the new and the formerly provided RNA sequencing experiments (new Fig. 3h; new Extended Data Fig. 7. d,f) warrant further discussion, specifically regarding the exonic reads in the DIS.

In response to this comment, we investigated the exonic reads from the DIS directly. Our new library preparation method and RNA sequencing identify the 3′-ends of RNAs. In the case of an SQC B^{*D} spliceosome complex, in which branching has not occurred, the 3′-end of the associated pre-mRNA would be an endogenous poly(A) tail, if the pre-mRNA has been cleaved and polyadenylated. Indeed, the majority of the reads in protein coding genes within the subset of non-excised intron reads, map to gene 3′ ends or, alternatively, to A-rich sequences within pre-mRNAs or mRNAs, a well-known result of oligo-T mis-priming. While these reads are therefore

uninformative regarding the splicing status of specific introns, a subset of exon-containing reads that span from the exon into the intron can be used to directly assess branching status. To test whether pre-mRNA-associated complexes in the DIS sample contain branched introns, we generated a metaprofile of these exon-to-intron reads aligned relative to branch points. This analysis shows no evidence of truncation at the branch point (Response Fig. 1.2) and further indicates that the detected pre-mRNAs are associated with introns that have not yet undergone the branching reaction, ruling out a significant contribution of lariat-intermediate-containing complexes to the DIS sample. While we believe it unlikely, as discussed below, we cannot exclude that a small proportion of complexes with a similar protein composition to the DIS complex that formed from SQC before pre-mRNA branching (Step I of splicing) may be present in our sample, and we discuss this possibility in the Extended Data Fig. 7f figure legend in the revised manuscript (Lines 2003-2008).

Several lines of evidence argue against a major contribution of pre-catalytic or SQC-derived complexes to the DIS sample. Importantly, we observe no cryo-EM density for RNA in the 5′-exon channel of the DIS reconstruction (e.g. at U5 stem loop I), indicating that the structurally characterized complex is not bound to pre-mRNA or a 5′-exon-intron intermediate. This contrasts with reported pre-catalytic and SQC-derived spliceosome structures (Zhan et al., Nat. Commun., 2024; Li et al., Cell Res., 2025; Soni et al., Nat. Struct. Mol. Biol., 2025). Furthermore, sucrose gradient purification prior to cryo-EM analysis likely depletes large pre-mRNA-bound assemblies from our sample. In addition, while remodeling of a B^{*D} spliceosome by DHX15 and DHX35 may yield a complex of similar protein composition to the DIS (but bound to pre-mRNA), such complexes may be unstable in vitreous ice and/or be very lowly abundant in our sample. Consistently, we have not observed evidence of a B^{*D} spliceosome in our cryo-EM DIS data, despite our large dataset and extensive cryo-EM image analysis. A detailed investigation and structural characterization of such potential SQC intermediates represents an interesting question for future investigation beyond the scope of the current manuscript.

We believe that the new and improved RNA sequencing experiment, which directly assesses branching status at individual introns transcriptome wide, provides more comprehensive and definitive evidence of RNA branching status than we previously provided and in comparison to an intron-specific primer extension assay. Taken together, our RNA sequencing and cryo-EM data show that the DIS complex is primarily associated with excised and debranched introns.

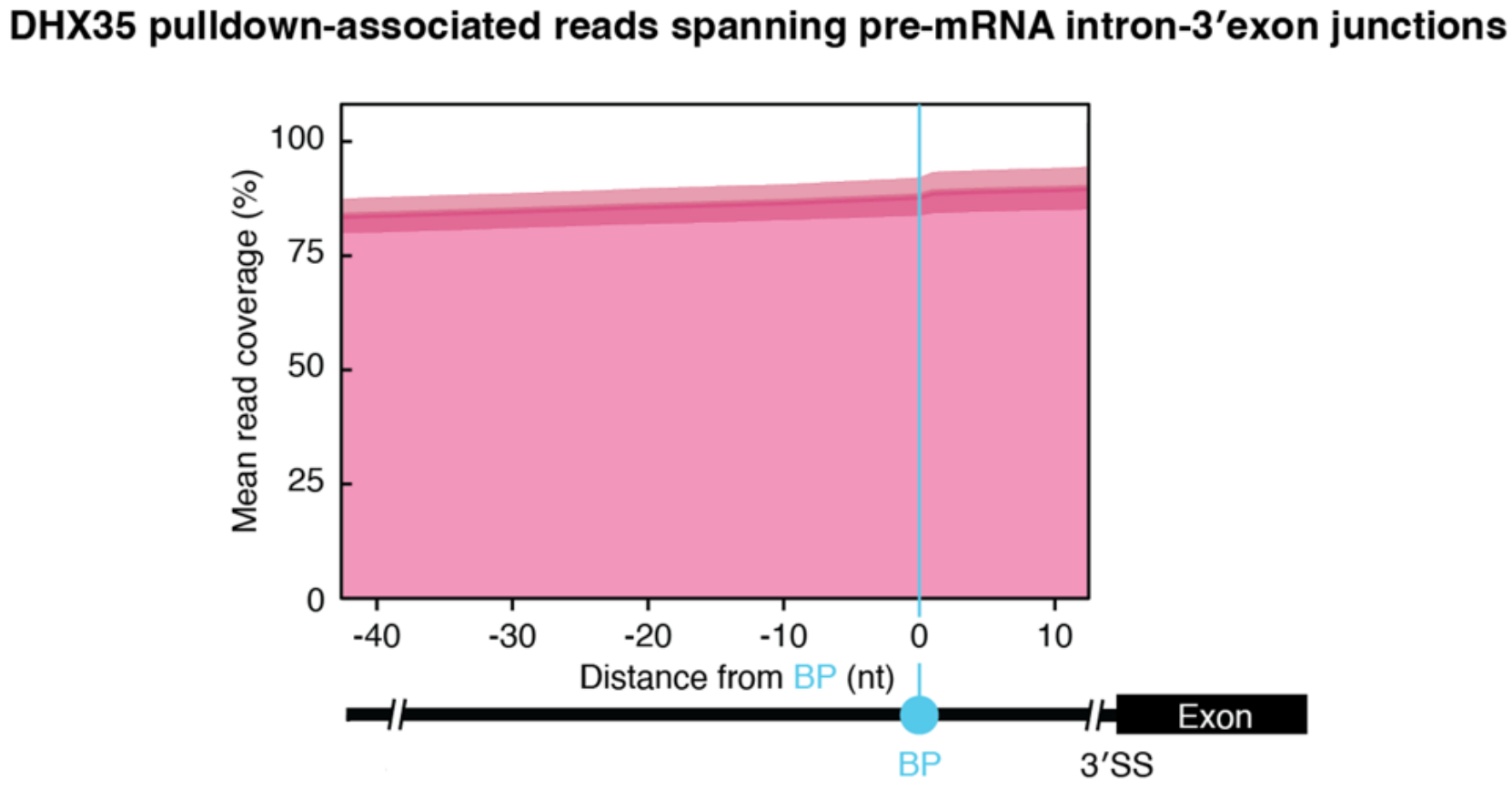

Response Fig. 1.2 l A subset of DHX35-IP associated reads contains unbranched pre-mRNA introns. *Metaplot of mean read coverage of a subset of pre-mRNA intron reads that extend into the downstream exon by at least 5 nucleotides in the DHX35-IP (DIS complex sample) centered around the major annotated branch point. 23,919 intron-exon pairs were included, representing 68,724 total reads (compared with 89,807 excised introns and 479,338 excised intron reads in the new Fig. 3g for the excised intron analysis of the DIS). Shaded regions indicate 95% confidence intervals.*

6. The DHX35 disassembly assay is incomplete. The authors do not show what happens to the RNA components, which would be key to conclude disassembly state – e.g. is U6 snRNA released, as implied by the authors' argument on p. 15-16 ? what happens to the associated introns in these assays ?

To address the reviewer's comment, we probed the snRNA components in the DIS release assay, which we originally used to measure the ATP-dependent release of the spliceosome components from bead-immobilized DHX35. In the new experiment, we treated bead-immobilized DIS complexes with ATP or ADP, removed the released material, performed extensive bead washes and analyzed the remaining bead-bound complexes (Response Fig. 1.3a). As expected, spliceosome proteins, but not DHX35 or WDR83, were depleted from beads after ATP treatment (Response Fig. 1.3b). Northern blotting revealed that U5 and U6 snRNAs were also depleted from ATP-treated beads compared to ADP controls (Response Fig. 1.3b). The low abundance of any single intron species (maximally representing ~0.5% of total intronic reads), and the limited yield from native DIS purifications, prevents us from quantifying the fate of individual intron RNAs. Taken together with our previous mass spectrometry analysis of the released fractions (currently Extended Data Fig.

8d-f), the data strongly suggest spliceosome release from DHX35, and therefore disassembly, owing to its helicase activity.

Importantly, our two RNA sequencing experiments (new Fig. 3f-i; new Extended Data Fig. 7d-f) together with the structural data demonstrate that the introns associated with the DIS are excised and predominantly debranched, defining the state in which these introns must be released from this terminal spliceosome complex. We provide further evidence for this in the next paragraphs below.

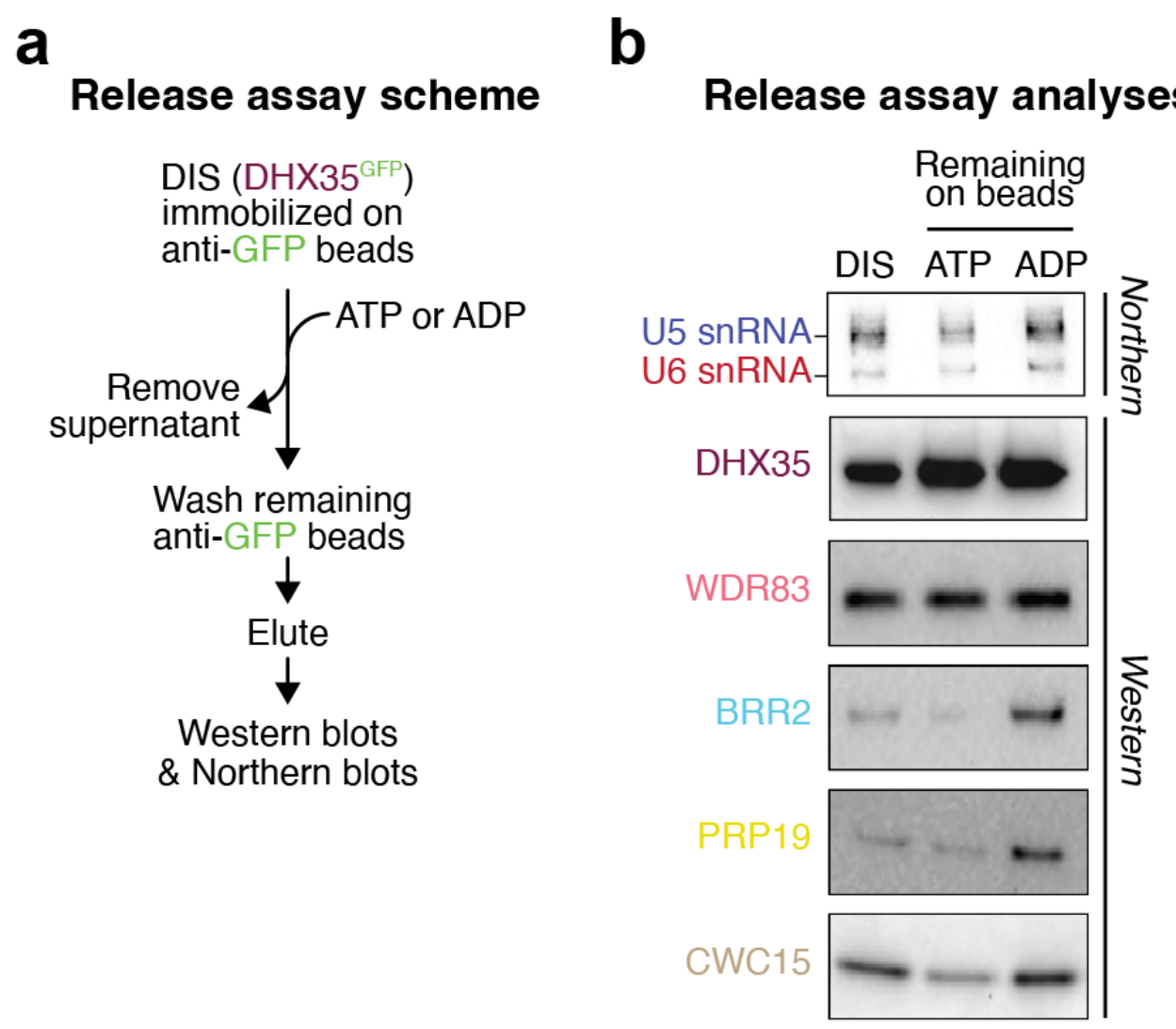

Response Fig. 1.3 | U5 and U6 snRNAs are released from DHX35 in a DIS release assay. a. *Experimental scheme of the updated DIS release assay.* ***b.*** *Northern and Western blots of the material remaining bound to bead-immobilized DHX35 in the DIS release assay. Both snRNAs and spliceosome proteins decrease upon ATP treatment compared to ADP treatment. WDR83 remains anchored to bead-immobilized DHX35 through the shared interactor GPATCH1.*

If the authors want to properly support their sequential model, they should actually carry out a similar assay for the ILSAQR state and compare the state of the introns (branched, debranched) in the resulting released fractions. Related to this, would DBR1 dissociate from ILSAQR in this assay ?

Thank you for the suggestion. We wish to clarify that DBR1 is not observed in the ILS^{AQR} spliceosome cryo-EM structure. We have now modified Fig. 2 to label the DBR1-C19L1 AlphaFold prediction more clearly, in addition to the previously provided descriptions in the main text (Lines 205-207) and Fig. 2b,e figure legends. The association of DBR1 with the spliceosome was established in our manuscript

through in vitro biochemical data, native mass spectrometry, and a genetic depletion-rescue assay (Fig. 2, Extended Data Fig. 5). These results show that DBR1 binds to C19L1 in vitro (Extended Data Fig. 5g) and in vivo together with the rest of the ILS (Fig. 2f, Extended Data Fig. 5a,b), and that the C19L1-DBR1 interaction is essential for cell viability (Extended Data Fig. 5h). Because DBR1 is not stably associated with purified ILS^{AQR} complexes, we cannot perform the experiment suggested by the reviewer. However, multiple independent lines of evidence support a sequential ILS^{AQR}-to-DIS transition:

1. *In the submitted manuscript, we provided an ILS remodeling assay from nematode C. elegans extract, which supports the sequential ILS-to-DIS transition (Extended Data Fig. 1a). Specifically, total C. elegans extract was incubated with or without ATP, and spliceosomes were subsequently purified by a pulldown using 3xFlag-tagged PRP19 as a bait. The protein composition of ATP-treated and untreated samples was compared by quantitative mass spectrometry. In this experiment, we observed the loss of ILS-specific termination factors upon ATP treatment, showing that ILS was being remodeled. Importantly, we also observed increased binding of the DIS-specific factors DHX35 and GPATCH1 to these remodeled spliceosomes. This strongly suggests that the DIS is a product of ATP-dependent ILS remodeling. This experiment was uniquely possible in C. elegans, because purified C. elegans spliceosomes at steady-state are apparently entirely found in the ILS state (Vorländer et al, Nature, 2024), in contrast to human spliceosomes (see below).*

2. *We conducted a new experiment to confirm the ILS-to-DIS transition in the human system. Because ILS complexes do not dominate the spliceosome population in K562 cells, in contrast to C. elegans extract (see above), a PRP19-based pulldown would capture a mixture of spliceosome states undergoing simultaneous remodeling (e.g. the simultaneous remodeling of P complex to ILS, of ILS to DIS, and further DIS remodeling) and thus complicate interpretation. We therefore designed an alternative approach using the purified ILS^{AQR}, which we had purified via its association with YJU2B.*

   We first incubated the nuclear extract of GFP-YJU2B expressing K562 cells with or without ATP and then performed an anti-GFP pulldown and analyzed the bound spliceosome proteins by Western blotting (new Extended Data Fig.

7a). In this reaction, spliceosomes remained bead-immobilized with YJU2B, as shown by the comparable amounts of U5 snRNP subunits PRP8 and BRR2 and the NTR subunit AQR in both ATP-treated and -untreated conditions (new Extended Data Fig. 7b). In contrast, the ILS-specific termination factors, such as TFIP11, C19L1 and C19L2 deplete from spliceosomes after ATP treatment, demonstrating that ILS^{AQR} is being remodeled, similar to the outcome in the C. elegans extract experiment (new Extended Data Fig. 7b). We also note that the spliceosome complexes remaining associated with YJU2B in this remodeling assay may indicate a possible intermediate between ILS^{AQR} and DIS that still contains YJU2B, supporting the idea raised by the reviewer below that intermediate states between ILS and DIS may exist. While this extends our model with potential intermediates, it does not influence our conclusions (see response to comment #7).

We then investigated whether the remodeled ILS^{AQR} can transition to a DIS-like state. The dissociation of C19L2, which blocks the GPATCH1-DHX35 binding site from the ILS^{AQR} during remodeling, would be expected to facilitate the association of GPATCH1 and DHX35 with this complex. We could not detect endogenous DHX35 or GPATCH1 in either ATP-treated or untreated samples using their various antibodies, which may owe to the low abundance of these proteins in the nuclear extract and low antibody sensitivity. Therefore, we instead co-expressed recombinant human GPATCH1–DHX35 in insect cells, and purified this heterodimer, carrying a 3xFlag-tag and an active site mutation (K83A) in DHX35 and a TwinStrep-tag on a truncated GPATCH1 construct (residues 1-440). The truncated GPATCH1 showed a much better biochemical behavior than the full-length protein, and contained the GPATCH1 regions required for the binding of DHX35 and PRP8 within the DIS. The two affinity tags allowed us to detect DHX35 and GPATCH1 with antibodies against their respective tags. We then added this recombinant heterodimer GPATCH1–DHX35 to the remodeling assay and bead-immobilized ILS^{AQR} (via YJU2B-GFP) from ATP-treated or untreated conditions. Western blot analyses showed increased binding of the recombinant GPATCH1–DHX35 heterodimer at both tested concentrations, specifically when the ILS^{AQR} complex had been ATP-treated and had undergone remodeling (new Extended Data Fig. 7b). These observations are consistent with ILS^{AQR} being remodeled to a DIS-like complex in the human

nuclear extract, similarly to what we report in C. elegans extract.

3. *Our two RNA sequencing experiments show that ILS^{AQR} is associated with branched intron-lariats, while DIS is bound to excised debranched introns. Importantly, using the new RNA sequencing experiment, which had a much greater sequencing depth than the former experiment, we can show that the great majority of introns found in ILS^{AQR} are also present in the DIS complex (new Fig. 3i). This is despite the ILS^{AQR} sample having 20-fold more RNA sequencing reads than the DIS sample, reflecting different pulldown yields. Specifically, the new pie chart in main text Fig. 3i, shows that among the top 50% most abundant introns in ILS^{AQR}, 92.5% are also detected in the DIS. The converse is also true with virtually all DIS introns being found associated with the ILS^{AQR} (Response Fig. 1.4). The abundance of shared introns strongly suggests that the two complexes are part of the same pathway. This is also consistent with our proposed model of intron-lariat debranching on the spliceosome, in which the debranching enzyme DBR1 binds to the ILS owing to its interaction with the ILS-specific termination factors C19L1 (Fig. 2e,f; Extended Data Fig. 5) and debranches the majority of associated intron-lariats during the remodeling to DIS.*

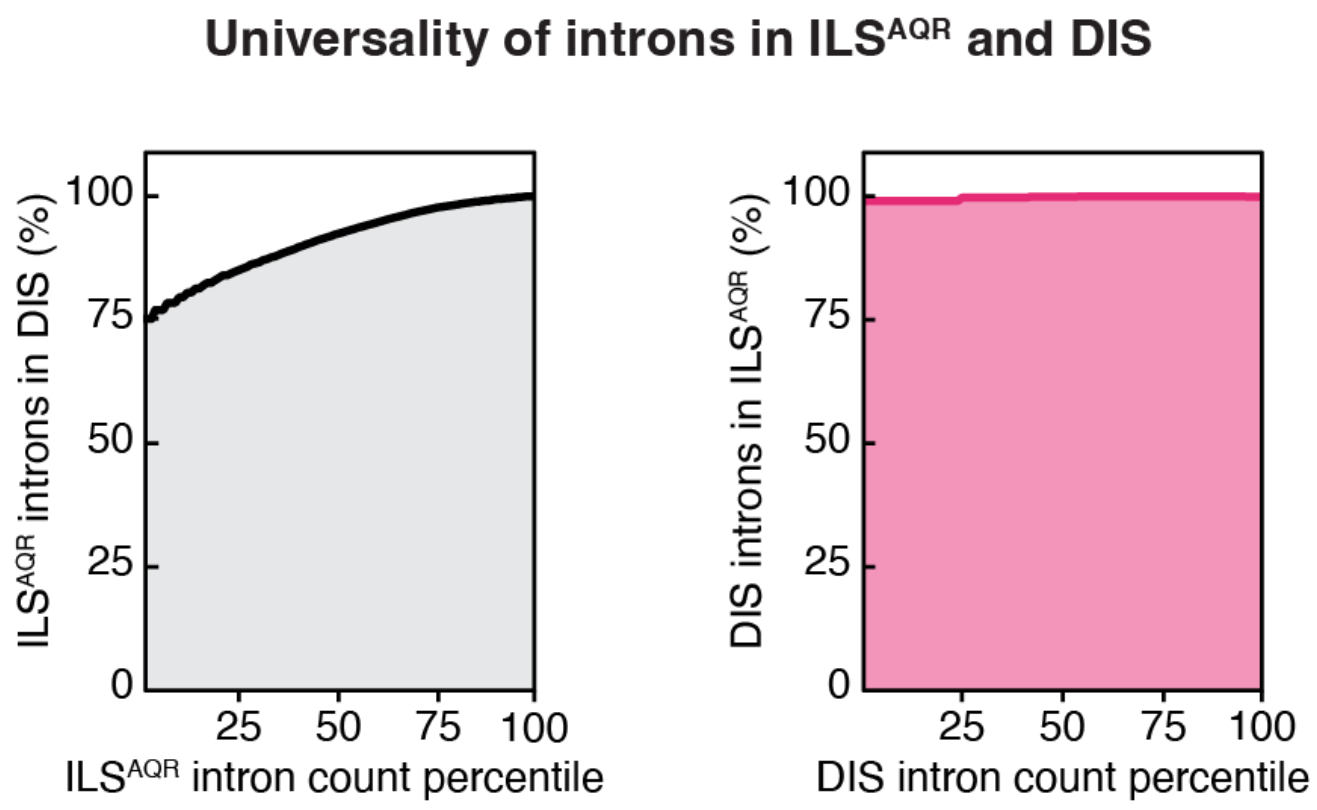

Response Fig. 1.4 | Evidence for a general sequential transition between ILS^{AQR} and DIS. *Plots showing the percentage of ILS^{AQR} introns of various abundance levels present in DIS (left) and the percentage of DIS introns of various abundance levels present in ILS^{AQR}. A count percentile of 0 corresponds to the inclusion of every intron in the dataset, while a percentile of 99 corresponds to only the 1% most covered introns in the dataset.*

4. *The Lührmann and Makarov labs have previously studied the 35S snRNP complex, the final spliceosome intermediate that accumulates in an in vitro*

splicing reaction only at the very end of splicing (Makarov et al., 2002, Science; Makarova & Makarov, 2015, PLOS One,). Biochemical characterization of the formerly purified 35S complex by the Lührmann lab shows that the 35S snRNP comprises the U5 snRNP, NTC, NTR, DHX35, GPATCH1, WDR83, with some also detecting the presence of U6 snRNA (Makarov et al., 2002, Science,; Wahl et al, Cell, 2009; Golas et al., Mol. Cell, 2010; Makarova & Makarov, PLOS One, 2015). This composition closely resembles the DIS complex described in our current manuscript, suggesting that DIS and 35S likely represent the terminal state of the spliceosome forming after the ILS and just before disassembly, as was indeed proposed previously (Makarov et al., Science, 2002; Makarova & Makarov, PLOS One, 2015; Yoshimoto et al., Nucleic Acids Res., 2009; Wahl et al, Cell, 2009).

Taken together, the cryo-EM, biochemical, and RNA sequencing data support that the DIS complex forms after remodeling of the ILS.

Sequential intron debranching and extraction model and aberrant s'some disassembly

7. Can the authors exclude the possibility that their DIS state is actually a combination of multiple complexes in the final stages of disassembly ? The occlusion of the DHX35 binding interface in earlier complexes presented in EDFig. 7e may be disrupted by the action of the specific DEAH-box proteins bound to those complexes during putative proofreading steps, making the interface accessible to engagement by DHX35. Indeed, the previous S.pombe DHX35 study proposed exactly such a mechanism at the B* stage. In my view such a possibility would actually strengthen the authors' argument for a final disassembly mechanism common throughout the splicing pathway and for both canonical and aberrant splicing. Such a possibility is also not inconsistent with a predominantly debranched state of the substrate, especially if discard would occur after chemistry at earlier stages such as C.

We thank the reviewer for these comments. We believe that our conclusion that the DIS complex we report originates from canonical spliceosome termination is well-supported, based on three lines of evidence: (1) we observed no density for RNA in the 5′-exon channel of DIS cryo-EM reconstructions, (2) RNA sequencing shows that the DIS is predominantly associated with excised and debranched intron, consistent with the structurally characterized complex being not bound to pre-mRNA; and (3) the introns associated with the DIS are shared with the ILS^{AQR} which together with our supporting biochemical results from human and C. elegans extracts support the

DIS being part of the canonical spliceosome termination pathway. Additional details are discussed in our responses to the above comments #5 and #6.

Remodeling of a B^{*D} complex could in principle yield a complex of similar protein composition to the DIS, as proposed in S. pombe (Soni et al., Nat. Struct. Mol. Biol., 2025) and C. thermophilum (Li et al., Cell Res., 2025). However, as noted above, the DIS cryo-EM reconstructions show no density for RNA in the 5′-exon channel or the proteins LENG1 and CWC22, which would be expected in a B^{*D} SQC complex (see our response to comment #8). In addition, the few pre-mRNA-associated introns detected in the DIS sample show no evidence of branching (Response Fig. 1.2), indicating that these pre-mRNAs have not undergone the first catalytic step. Thus, while we cannot exclude a potential SQC origin of a small fraction DIS-like complexes in the sample, which we acknowledge in the revised manuscript (Lines 2003-2008; Extended Data Fig. 7f), we believe that the DIS cryo-EM structural model is unlikely to derive from SQC.

We agree with the reviewer that a conceptionally shared disassembly mechanism for both canonical and aberrant splicing pathways would be consistent with our biochemical and structural data and would further support the generality of DHX35-dependent termination. We discuss this on pages 22-23 of the revised manuscript. This possibility represents an interesting direction for future work.

Related to this, the MS data suggest DBR1 associates with the proposed PAXBP1, which is the GCFC2 paralog and proposed to be involved in complexes containing both DXH35 and DHX15, at least based on the C. termophilum structures (and the authors' own suggestion if Figure 5c). These observations again point to the possibility that the pathway ordering may not be as direct as the authors imply in the model Figure 5b and that DHX35 and DHX15 might co-exist in complexes containing DBR1 (the authors don't mention if any DHX35 is detected in MS of DBR1 IP).

Thank you for these comments. We would like to point out that, in contrast to the human ILS^{AQR}, PAXBP1 (not GCFC2) is the relatively more abundant paralog in C. elegans ILS complexes and human ILS″ complexes (Vorländer et al., Nature, 2024). Notably, both PAXBP1 and GCFC2 are detected by mass spectrometry in all the ILS complexes purified by our group (Vorländer et al., Nature, 2024; and current study). The cryo-EM density resolution in these regions is not sufficient to discriminate between the two paralogs, which is why in the final coordinate models we include the more abundant paralog as determined by quantitative mass spectrometry of the

respective samples. For instance, GCFC2 is ~5-fold more abundant than PAXBP1 in the YJU2B pulldown (new Extended Data Fig. 1g), while PAXBP1 is ~8-fold more abundant in the TFIP11 pulldown previously used to obtain the human ILS″ sample (Vorländer et al., Nature, 2024). From the available data it is, therefore, not clear, if PAXBP1 or GCFC2 discriminate between specific spliceosomal states (for instance, an ILS from an SQC complex), or whether they bind either state indiscriminately. We now more explicitly acknowledge this in the updated Supplementary Text 2.

DHX35 and GPATCH1 are indeed enriched in the DBR1 pulldown, albeit to a lesser extent than the ILS-specific termination factors (updated Extended Data Fig. 5b). We agree with the reviewer that intermediate states between ILS^{AQR} and DIS may exist, for example, in which GPATCH1–DHX35 associates with the spliceosome before C19L2–C19L1 and DBR1 are fully released. Our biochemical data support this possibility, as YJU2B and DHX35–GPATCH1 can co-exist in the same complex in vitro, as seen in the newly added ATP-driven remodeling assay of the ILS^{AQR} (see response to comment #6). However, such intermediates are likely short-lived and may not be sufficiently stable for purification from nuclear extracts, as our native steady-state pulldowns capture biochemically stable and long-lived states, which is the case for the ILS^{AQR} and DIS complexes. We now acknowledge in the revised manuscript (Lines 289-290, lines 860-862 in figure legend of Fig. 5a) that uncharacterized intermediates between the ILS^{AQR} and DIS may exist. We note that the key features of the pathway, such as the loss of ILS-specific factors, intron debranching, and WDR83–GPATCH1–DHX35 recruitment, are well-supported by our structural, biochemical, and RNA sequencing data.

I think the authors need to show the relative abundance of all relevant disassembly factors in all of their MS data, for transparency, and discuss more explicitly what would prevent GPATCH1/DXH35 recruitment to a defective (or for that matter normal) spliceosome containing a branched intron during the catalytic stage. Is the implication that GPATCH1 would only recognize linear introns? If so, I think it is important to make this point explicitly (and provide more evidence that DHX35 does not associate with complexes containing branched RNAs).

We thank the reviewer for noting the MS data. We have now updated the heat maps of the DHX35 and YJU2B IP-mass spectrometry experiments (Extended Data Fig. 1g) to include PAXBP1, and the heat map of the DBR1 pulldown (Extended Data Fig. 5b) to include GCFC2, DHX35, GPATCH1 and WDR83. These heatmaps show that PAXBP1 and GCFC2 are the predominant orthologs in the DBR1-GFP and

YJU2B-GFP pulldown samples, respectively. Similarly, the newly included data reveal the presence of DHX35 and GPATCH1 in the DBR1 pulldown, which may indicate the presence of unidentified intermediates, as discussed above. We agree that including these data allows for greater comparison and transparency.

Next, we would like to address the reviewer's question about GPATCH1–DHX35 binding to catalytic-stage spliceosomes containing branched introns. Based on the structural overlays, the binding sites for GPATCH1–DHX35 observed in the DIS (and the S. pombe B^{*D} complex) are not accessible in the structures of on-pathway C and C/P state spliceosomes, which contain branched introns, because they are instead occupied by step-specific splicing factors DHX38 and CACTIN, respectively (see current Extended Data Fig. 8c). However, we agree that the destabilization of an aberrant spliceosome and resulting dissociation of step-specific factors might expose the PRP8 surfaces needed for GPATCH1 binding. Consistent with this possibility, GPATCH1–DHX35 have been detected by mass spectrometry in preparations of in vitro assembled C complex spliceosomes (Agafonov et al., Mol. Cell. Biol., 2011), suggesting that DHX35 is not excluded from complexes containing branched intron RNAs. We thus hypothesize that the availability of this PRP8 surface may serve as the key feature for GPATCH1–DHX35 recruitment, for instance, preventing their stable engagement to the C complex during successful splicing.*

In the B^{*D} complex, GPATCH1 contacts a single "bulge" nucleotide of the pre-mRNA intron (Soni et al., Nat. Struct. Mol. Biol., 2025), and it is currently unclear whether this would provide sufficient specificity and affinity to discriminate between canonical and aberrant spliceosomes. While we do not exclude a possible role for GPATCH1–DHX35 after intron branching in canonical or aberrant spliceosomes, we do not propose that GPATCH1 specifically recognizes linear introns. Instead, we suggest that GPATCH1–DHX35 recruitment is controlled primarily through the accessibility of the PRP8 binding surface (see paragraph above). Importantly, any possible role of GPATCH1 after branching would not affect our conclusions regarding GPATCH1–DHX35 in canonical spliceosome termination, since the DIS reported here is predominantly associated with excised and debranched introns and is part of the regular spliceosome termination pathway.

8. The revised modelling of LENG1 in the S.pombe B*D structure is compelling, and the additional predictions and proximity labelling in the human system provide some support for LENG1 interacting with disassembly factors. However, in my view it remains unclear whether a similar B*D complex actually exists in humans. This is

important to determine because in B*D both so-called ILS factors (DHX15, TFIP11) and DIS factors (DHX35,GPATCH1) are all present at the same time as the newly identified LENG1. However, in the current human study, the authors suggest that ILS factors dissociate during DHX15/AQR-mediated remodelling to allow DHX35 engagement. The existence of a similar B*D complex in humans would imply that either the pathway for disassembly of defective complexes is somewhat different, as the authors do allude to in their final discussion on p. 20-21, or that the authors' sequential model needs to be revised and one needs to potentially rethink to what extent DBR1 may be acting during the transition, on the spliceosome, or potentially after release of the intron in the case of aberrant spliceosomes that arrest following branching e.g.. Are the authors implying that defective spliceosomes that have undergone branching but cannot complete exon ligation would not be targeted by the mechanisms they describe ?

We thank the reviewer for these comments. We agree that the mechanism of termination of faulty spliceosomes will carry differences compared to canonical termination, and we have revised the discussion on pages 22-23 to describe these differences in more detail. We address the role of LENG1 and its presence in human SQC complexes further below.

The key distinction between the two complexes is in the arrangement of the RNA network and the protein factors present in each state. For canonical termination (ILS) the branch helix remains intact and is stabilized by the ILS-specific factor C19L2, which occludes the PRP8 surface required for GPATCH1–DHX35 binding (Extended Data Fig. 6b). Canonical termination therefore first requires a remodeling step, likely mediated by DHX15 and AQR, to extract the branch helix from the active site and displace C19L2, which allows GPATCH1–DHX35 to bind and form the DIS. DHX35 would then complete termination by pulling on U6 snRNA and ejecting the intron. In the B^{*D} complex, by contrast, the branch helix has already been disrupted, possibly because the bulged-out intron nucleotide prevents branch helix docking in the first place (Soni et al., Nat. Struct. Mol. Biol., 2025). Importantly, in B^{*D}, the ILS-specific protein C19L2 is absent, as it cannot associate owing to the presence of the pre-mRNA 5′-exon in the spliceosome's 5′-exon channel. This means that the PRP8 surface in the B^{*D} is directly accessible to GPATCH1–DHX35 without a prior remodeling step. This explains why ILS-specific factors (TFIP11, DHX15) and DIS-specific factors (DHX35, GPATCH1) can coexist in B^{*D} but occupy sequential intermediates during canonical termination in ILS and DIS complexes. We note that

for the revision we now also provide biochemical evidence for a human SQC complex, which we describe below on page 27 of this response.

The two-step model during canonical termination also enables debranching of the majority of intron-lariats on the spliceosome by DBR1 (see also response to comment #5). DBR1 would associate with the ILS through its direct physical interaction with C19L1 (Fig. 2e,f; Extended Data Fig. 5), consistent with our depletion-rescue experiment showing that this interface is essential for human cell viability (Extended Data Fig. 5h). We note that C19L1–C19L2 are not part of the B^{*D} complex, consistent with DBR1 recruitment being specific to the canonical pathway and the interaction with C19L1.

Regarding the reviewer's question about aberrant spliceosomes that have undergone branching but cannot complete exon ligation: as discussed in our response to comment #7, we cannot exclude that destabilization of such spliceosomes could expose the PRP8 surface required for subsequent GPATCH1–DHX35 binding, and that DHX35 could in principle act on such branched intron complexes (consistent with its detection in C complex preparations; Agafonov et al., Mol. Cell. Biol., 2011). Resolving whether DHX35 can act on branched pre-mRNA introns during SQC will require the isolation of such intermediates, which is beyond the scope of the present study. Our data specifically address canonical termination of spliceosomes that have completed both catalytic steps, and we find no direct evidence in our data for DHX35 acting on branched pre-mRNA introns during SQC.

The authors should at least clarify whether in their MS experiments they have observed any evidence of LENG1 associating with either ILSAQR or DIS. Related to this it isn't immediately clear why LENG1 could not associate with YJU2B ZnF in the ILS state; is it really impossible that LENG1 may be loosely associated but only engage the core if a particular intron configuration is present in the core and if CWC22 is still engaged with the spliceosome ? A figure to clarify this would be important.

We did not detect LENG1 in the DHX35 pulldown sample (DIS) by mass spectrometry, which may reflect the transient association of LENG1 with DHX35-containing complexes during aberrant termination and the low abundance of SQC complexes relative to DIS in this sample. We did detect LENG1 together with DHX35, GPATCH1 and WDR83 in the YJU2B pulldown, albeit with LENG1, DHX35, GPATCH1, and WDR83 being detected at much lower levels compared to the

components of the ILS^{AQR}, the canonical terminating spliceosome (Response Fig.1.5a). This is consistent with LENG1 associating with YJU2B in cells in our LENG1 proximity labeling experiment (currently Extended Data Fig. 8e) and our new native LENG1 pulldown (see below). However, none of these factors (LENG1, DHX35, GPATCH1, WDR83) are present in the gradient-purified ILS^{AQR} complex that we used for cryo-EM analysis and RNA sequencing (Response Fig. 1.5a). We, therefore, conclude that LENG1 can associate with complexes containing YJU2B and DIS factors, but that it is not stably incorporated into the ILS^{AQR}.

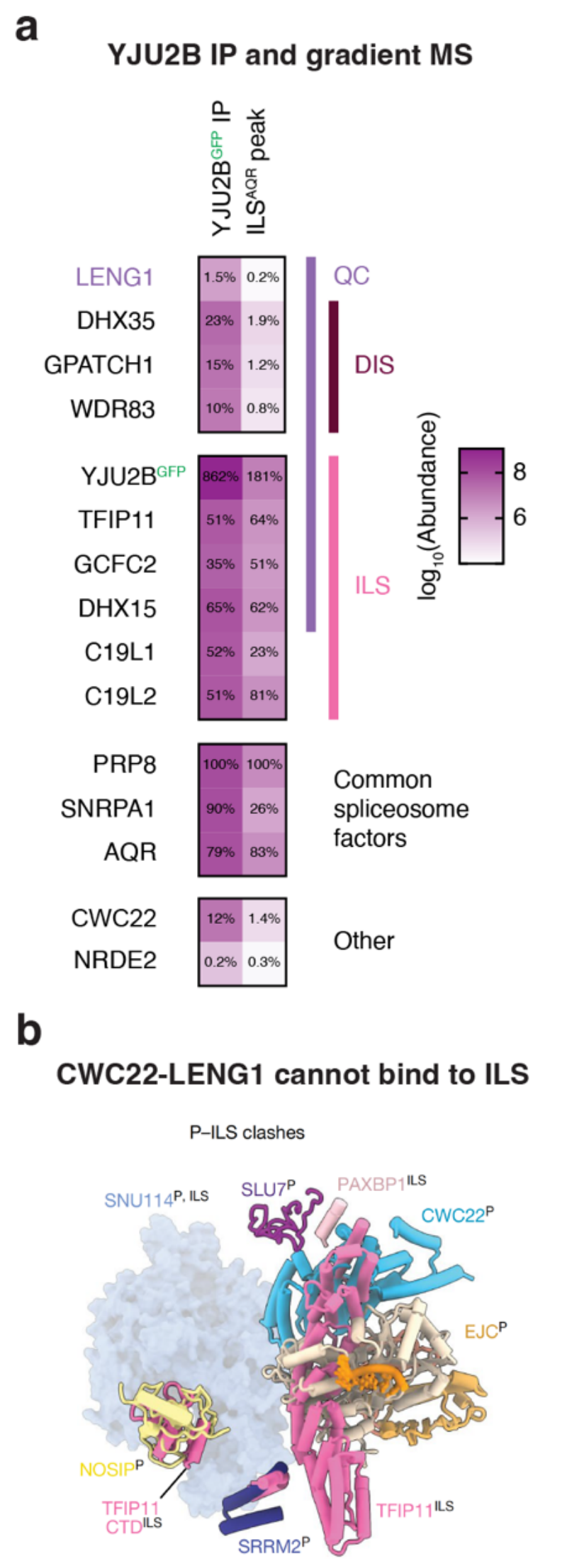

Reproduced from Fig. 3c in Vorländer et al., 2024

Response Fig. 1.5 | DIS and SQC factors associate with YJU2B but not with ILS^{AQR} in human cells. a. *Heatmap representing non-normalized abundances of relevant spliceosome factors in the native YJU2B-GFP pulldown before (YJU2B^{GFP} IP) and after sucrose gradient purification (ILS^{AQR} peak). The stoichiometry of each protein in both samples relative to the core spliceosome component PRP8 is*

indicated in each box. The exact same panel is shown in Response Fig. 2.2. ***b.*** *Structural comparison between P and ILS state spliceosomes, showing that CWC22, and by extension its interactor LENG1, cannot bind to ILS complexes. Panel reproduced directly from Fig. 3c in Vorländer et al., Nature, 2024.*

There are two structural reasons why LENG1 cannot bind YJU2B in ILS^{AQR}. First, the N-terminus of LENG1, which would bind the YJU2B ZnF, cannot engage YJU2B ZnF because this domain binds PRP8 in the ILS^{AQR} (new Extended Data Fig. 9f). Second, CWC22, the other LENG1-interactor, is released together with the spliced mRNP during the P-to-ILS complex transition (Chung et al., Nucleic Acids Res., 2025) and is therefore absent from the ILS^{AQR} (also see mass spectrometry data in Response Fig. 1.5a). Structurally, this is because CWC22 binds in a mutually exclusive manner with the termination-specific factor TFIP11 to the spliceosome, and TFIP11 occupies this site in the ILS^{AQR}(Response Fig. 1.5b, reproduced from Vorländer et al., Nature, 2024). Taken together, the data do not support a model in which LENG1 is loosely associated with the ILS. The data instead suggest that LENG1 can only engage the spliceosome when its binding interfaces on YJU2B and CWC22 become accessible, which does not occur in the ILS state. We now provide Extended Data Fig. 9f to visualize the structural incompatibility of LENG1 binding to YJU2B in the ILS^{AQR}. We further clarify that the LENG1 binding partner CWC22 is not available during regular termination in the main text (Lines 450-452).

It is possible that in the S. pombe system the observed B*D complex can only be captured associated with the 3'-5' degradation machinery, and this might explain why LENG1 would not associate with YJU2B or DHX35 complexes at steady state. The authors should try to purify, for MS purposes, LENG1-associated complexes, to help address the points raised above about ordering in the pathway for normal and aberrant splicing.

We thank the reviewer for the comments. We indeed detect only very low levels of the nuclear exosome regulator NRDE-2 (S. pombe Nrl1) in our human YJU2B pulldown, whereas NRDE-2/Nrl1 was used in the S. pombe study as a bait for the B^{*D} complex purification (Response Fig. 1.5a). This indicates that the NRDE-2, and potentially the RNA exosome, interaction with the human spliceosome could be transient. Consistent with this, Flemr et al, RNA, 2023, reported the association of NRDE-2 with spliceosomes (including B^{*D} components: TFIP11, GPATCH1, DHX15, WDR83) and the exosome-linked RNA helicase MTR4 only after chemical cross-linking of mouse nuclear extract, thereby stabilizing transient and low-affinity

interactions. It is therefore possible that mammalian SQC complexes are more efficient at disassembly and/or that fewer introns are targeted by this SQC pathway at steady state in comparison to S. pombe. These observations could explain why our DHX35 and YJU2B pulldowns from human cells yielded regular terminating spliceosomes rather than SQC intermediates.

To directly probe whether a B^{*D}-like complex may exist in human cells, we performed a LENG1 pulldown experiment. For this experiment we turned to human RPE-1 cells because the retinal epithelium, from which these cells are derived, is a tissue well-known to be particularly sensitive to defects in pre-mRNA splicing (Yang et al., Front. Cell Dev. Biol., 2021). We therefore hypothesized that RPE-1 cells may be particularly prone to accumulating SQC events at steady state in comparison to other cell lines. We generated a stable cell line ectopically expressing GFP-tagged LENG1. A pulldown using anti-GFP beads followed by mass spectrometry revealed a significant enrichment of spliceosomes, including the factors that bind jointly with LENG1 in the S. pombe B^{*D} spliceosome (DHX35, GPATCH1, WDR83, TFIP11, PAXBP1, CWC22) (new Extended Data Fig. 9i). These results support the existence of the B^{*D} defective spliceosome in human cells and the binding of the newly identified protein LENG1 in such complexes.

Additionally, a more detailed analysis of the introns observed in the ILSAQR and DIS complexes might shed light on whether potentially aberrantly spliced introns elicit formation of these complexes.

Based on our two RNA sequencing assays, the predominant intron RNA species associated with both ILS^{AQR} and DIS complexes are excised introns, which can only arise from successful two-step splicing. The truncation signatures and the read start sites of excised introns in the newly provided RNA sequencing experiment (described in detail in our response to comment #5) also indicate that these introns use canonical branchpoints and canonical 3′-splice sites. Furthermore, our analysis shows that the great majority of introns that can be observed in the ILS^{AQR} can also be observed in the DIS, consistent with the idea that the DIS complex is an on-pathway state of the canonical splicing reaction, rather than an off-pathway state observed for a subset of poorly or improperly spliced introns. As discussed above, DHX35 could also be associated with aberrant spliceosomes as part of an SQC complex, but this is not the dominant state observed in our dataset. Overall, we do not observe any evidence for the association of ILS^{AQR} and DIS complexes with aberrantly spliced introns.

Referee #2 (Remarks to the Author):

In this manuscript, Boreikaite and colleagues investigated the molecular mechanisms underlying spliceosome termination in humans, focusing on the sequential disassembly of spliceosomal complexes, intron-lariat debranching, and intron ejection. Using an integrated approach combining cryo-EM, biochemistry, RNA sequencing, and genetics, the authors identify two new termination related spliceosome states and multiple protein factors that have not been previously linked to spliceosomal termination. The first state is ILS-Aquarius (ILSAQR) which contains excised intron-lariat, termination factors, and RNA helicases DHX15 and AQR, representing an intermediate committed to disassembly. The second state is Debranched Intron Spliceosome (DIS) which contains a debranched intron tethered by partial RNA contacts and the RNA helicase DHX35 with cofactors GPATCH1 and WDR83, representing a later stage poised for intron ejection and recycling of the spliceosome. Additionally, the paper explores defective spliceosome termination, highlighting the role of YJU2B–LENG1 in recognizing aberrant pre-mRNA and guiding DHX35–GPATCH1–WDR83 to defective complexes. Overall the experiments are well designed, clearly presented, and the conclusions justified. The manuscript provides many novel insights into spliceosome termination, an understudied process of the splicing cycle, representing a significant contribution to our understanding of the molecular mechanism of pre-mRNA splicing. Below are a number of suggestions that can further strengthen the manuscript.

We thank the referee for the helpful comments and the positive evaluation of our work.

1. The authors identified the human termination complexes by examining the effects of incubating C. elegans ILS complexes were incubated with ATP which led to the enrichment of YJU2B and DHX35 among other proteins. They then isolated YJU2B and DHX35 containing complex from human cells. This raises the question of what would happen if human ILS complexes were incubated with ATP. Would it also lead to YJU2B and DHX35 containing complexes? This would provide strong evidence that these represent downstream complexes in the spliceosome disassembly pathway in humans.

We thank the reviewer for this suggestion. We wish to note that chasing ILS complexes that lack YJU2B to the YJU2B-containing ILS^{AQR} is not straightforward, because the ILS″-to-ILS^{AQR} transition would involve the ATP-independent binding of

YJU2B, which displaces CDC5L helix 357-382 from the combined PRP8-ESS2-SNW1 interface and repositions the metallophosphatase domain of C19L1. Further, the purification of ILS^{AQR} required the immunoprecipitation of GFP-tagged YJU2B, indicating that this complex is a relatively short-lived intermediate at steady state in human cells. Because YJU2B can also interact with the DIS-specific protein WDR83 (Fig. 1b, Extended Data Fig. 1b,c, 6e), we believe that ILS^{AQR} most likely arises from the ILS″ in an ATP-independent manner. We therefore focused our experiments on investigating whether the ATP-dependent remodeling of the ILS^{AQR} leads to the formation of the DIS complex. We investigated this by probing the binding of the DIS-specific protein GPATCH1–DHX35 to the remodeled ILS^{AQR} in the human system, as detailed below.

In contrast to the experiment in C. elegans extract (Extended Data Fig. 1a), where we only detected ILS complexes in the PRP19 pulldown at steady state, in human nuclear cell extract we observed a mixture of spliceosomal states, including C/P, ILS, and DIS complexes. Therefore, an equivalent PRP19-based pulldown to C. elegans would in human nuclear extract show a mixture of spliceosome states, which upon ATP-treatment would undergo remodeling simultaneously (e.g. P to ILS, ILS to DIS, and DIS to disassembly) and thereby complicate interpretation. We therefore took an alternative approach based on the purified ILS^{AQR}, which associates with YJU2B.*

We first incubated the human nuclear extract from cells expressing GFP-tagged YJU2B (used for the ILS^{AQR} purification) in the presence or absence of ATP (new Extended Data Fig. 7a). We then carried out an anti-GFP pulldown and analyzed the bead-immobilized spliceosome proteins by western blotting (new Extended Data Fig. 7b). In this remodeling assay, we observed that similar amounts of the U5 snRNP subunits PRP8 and BRR2 and the NTR subunit AQR were retained in both ATP-treated and -untreated conditions. In contrast, ILS-specific termination factors (TFIP11, C19L1, and C19L2) were depleted from bead-immobilized spliceosomes after ATP treatment, demonstrating that ILS^{AQR} was being remodeled, similarly to the experiment in C. elegans extract (Extended Data Fig. 1a and new Extended Data Fig. 7a,b). The depletion of C19L2, which occupies the GPATCH1–DHX35 binding site in the ILS^{AQR}, should render this site available for DIS factor association in the remodeled complex. We did not detect endogenous DHX35 or GPATCH1 in either ATP-treated or untreated samples using various available antibodies, which may be due to the low abundance of DIS factors in the nuclear extract and low antibody sensitivity. We therefore turned to an approach using recombinant GPATCH1-

DHX35. Using insect cells, we co-purified the biochemically stable heterodimer of 3xFlag-tagged catalytic mutant (K83A) DHX35 and TwinStrep-tagged GPATCH1 (residues 1–440). This truncated GPATCH1 construct contained the protein regions required for binding to DHX35 and the spliceosome in the DIS (largely to PRP8), and improved biochemical behavior. The two orthogonal affinity tags allowed us to probe for DHX35 and GPATCH1 with antibodies against their respective tags. We added this recombinant heterodimer to either ATP-treated or -untreated nuclear extract, and subsequently purified remodeled ILS^{AQR} intermediates using an anti-GFP pulldown on YJU2B. Western blot analyses showed an increased binding of the recombinant GPATCH1–DHX35 heterodimer at both tested concentrations, specifically when ILS^{AQR} had been treated with ATP (new Extended Data Fig. 7b). These observations are fully consistent with ILS^{AQR} being remodeled to a DIS-like complex in human nuclear extract, similarly to what we previously showed in C. elegans extract.

Previous work by the Lührmann and Makarov groups also supports the DIS being a terminal state of the spliceosome in pre-mRNA splicing reactions in vitro (Makarov et al., 2002, Science; Wahl et al, Cell, 2009; Golas et al., Molecular Cell, 2010; Makarova & Makarov, PLOS One, 2015,). Specifically, these works showed that a 35S snRNP complex accumulates at the very end of an in vitro splicing reaction. This 35S snRNP complex closely resembles the DIS, containing U5 snRNP proteins, NTC, NTR, DHX35, GPATCH1, WDR83, the U5 snRNA, with some also detecting the presence of U6 snRNA (Wahl et al, Cell, 2009, Golas et al, Mol. Cell, 2010). Further, like the DIS we report here, this complex also lacked U2 snRNA and associated proteins. These observations suggest that the 35S complex is likely to correspond to the DIS we report here, and that it is a terminal spliceosome state forming after the ILS and just before disassembly, as was proposed previously (Makarov et al, 2002, Science; Yoshimoto et al, Nucleic Acids Res., 2009; Wahl et al, Cell, 2009; Makarova & Makarov, PLOS One, 2015).

Furthermore, we have added a new RNA sequencing experiment in the revision, which also supports our proposed order of ILS^{AQR} and DIS states. In this new sequencing assay (shown in Fig. 3f-i) we identified introns associated with purified ILS^{AQR} and DIS complexes at much greater sequencing depth than in the formerly provided RNA sequencing experiment (now shown in Extended Data Fig. 7d-f). Using the new and improved RNA sequencing data we found that ILS and DIS complexes are bound to the same intron substrates, providing independent evidence that these complexes are part of the same termination pathway (please also see our response to Reviewer 1 comment #6 in sub-point 3; Fig. 3i; Response Fig. 1.4).

Taken together, these observations support the model in which the DIS forms after the ILS^{AQR}.

2. Line 100. Extended Data Fig. 1e, f does not seem to be the correct figures. Please check.

The reference to Extended Data Figs. 1e,f is correct, but we appreciate that its placement may have been confusing. In this sentence we highlight that "we purified endogenous spliceosomes from the nuclear extract of human K562 cells carrying a GFP-tag on either DHX35 or YJU2B". In Extended Data Fig. 1e, we show an experimental scheme of how we purified these complexes. In Extended Data Fig. 1f, we show SDS-PAGE lanes of both complexes purified according to the scheme in panel e. To avoid confusion, we have moved the reference to these Extended Data Figs. 1e,f earlier in the sentence in the revised manuscript (Line 103):

"To gain insight into the mechanism of spliceosome termination, we purified endogenous spliceosomes from the nuclear extract of human K562 cells carrying a GFP-tag on either DHX35 or YJU2B (Extended Data Figs. 1e,f), which placed at either end of the chain of identified interactions."

3. Line 152-153. It is difficult to see how the AQR RecA2 domain shifts position before and after RNA binding. It would be helpful to include a superimposition of the two structures.

We thank the reviewer for the suggestion. We have now included a new Extended Data Fig. 4d comparing the conformations of the RecA lobes in RNA-free and RNA-bound AQR in Ce ILS'' and Hs ILS^{AQR}, respectively. Briefly, based on the crystal structures of the structurally closely related SF1 family RNA helicase Upf1 solved in presence or absence of bound RNA (Chakrabarti et al., Mol. Cell, 2011), we expect that the two RecA lobes of AQR should position closer together upon RNA binding to adopt a 'closed conformation' in contrast to an RNA-free conformation of AQR, where the RecA lobes should be placed further apart (Extended Data Fig. 4d; top panels). Indeed, in the cryo-EM density of AQR within the ILS^{AQR} complex, we find that the RecA lobes close over the bound RNA (now in Extended Data Figs 4d; middle left panel). In contrast, we observe a more open positioning of the AQR RecA lobes in AQR in the human DIS and C. elegans ILS'' spliceosomes, in which AQR is not bound to RNA (Extended Data Fig. 4d; middle right panel and bottom panel).

4. Line 184, "… containing a debranched intron (see below)". Please indicate the specific section below where this is discussed.

We have adjusted the main text to refer the reader specifically to the relevant main text section "ILS^{AQR} is remodeled into the debranched intron spliceosome", where we discuss the DIS complex and the branching status of its associated introns (Line 194):

"The composition of the DIS complex that we isolated, representing a later stage of termination than the ILS^{AQR} and containing a debranched intron (see section ILS^{AQR} is remodeled into the debranched intron spliceosome), suggests that debranching occurs prior to intron-lariat ejection from the spliceosome."

5. Line 206-211, regarding the alphafold2 multimer prediction, MPLKIP shows a higher iPTM score than SYF1 and C19L1. Doesn't this indicate that MPLKIP is a more likely interaction partner than SYF1 and C19L1? How did the authors arrive at the opposite conclusion?

We thank the reviewer for this observation. We wish to clarify that the higher iPTM score for MPLKIP with DBR1 does not contradict the C19L1 and SYF1 binding to DBR1. Each of these three DBR1 interfaces occurs in different parts of the DBR1 protein, and thus all three predicted interactors could bind together at the same time, which we now clarify in the revised manuscript (Line 227, also updated Extended Data Fig. 5c in Lines 1870-1871). Specifically, MPLKIP is predicted to bind the catalytic metallophosphatase (MPP) domain of DBR1, while C19L1 and SYF1 interact with peptides I and II, respectively, in the C-terminal region of DBR1 (Fig. 2e). MPLKIP is a known DBR1 interactor, forming a constitutive heterodimer in human cells (Townley et al., Mol. Cell, 2023). Destabilizing mutations in MPLKIP reduce DBR1 levels in cells and have been implicated in human disease (Theil et al. EMBO Mol. Med., 2023). We also observe evidence for a stable interaction between DBR1 and MPLKIP in our DBR1 proximity labeling and pulldown data from cells using mass spectrometry (Fig. 2f; Extended Data Fig. 5b). In both experiments, MPLKIP is among the top enriched proteins, indicating a high-affinity interaction. These results suggest that spliceosome-associated DBR1 is most likely bound to MPLKIP, which we visualize in Fig. 2e, and that all three DBR1 interactions are likely to occur on the spliceosome.

6. Line 220-222: "We note that the NTR subunit SYF1 carries a highly similar motif to DBR1 peptide I (SYF1 residues 812–824), which may indicate an exchange among the two peptides at C19L1 during termination." Do the authors mean that DBR1 replaces SYF1 in binding to C19L1? It would be helpful if the authors could clarify this point and elaborate on the functional implications of this exchange.

Thank you for this question. It is conceivable that DBR1 peptide I could compete with a peptide of similar sequence in SYF1 for the binding to C19L1 and, therefore, the spliceosome. The SYF1 peptide would be predicted to bind C19L1 in cis, when both are present in the ILS. In this scenario, DBR1 peptide I would have to outcompete the SYF1 peptide in the ILS, potentially explaining why DBR1 associates transiently with ILS complexes at steady state.

Interestingly, the C19L1-binding peptide of SYF1 is immediately adjacent to an 'I-patch motif', which could bind to and inhibit the nearby DHX15 (Portugal-Calisto et al., Nat. Commun., 2024) (Fig. 2b). Displacement of SYF1 by DBR1 peptide I from C19L1 might help to release the inhibitory I-patch from DHX15, owing to reduced avidity, promoting DHX15 activation. In this way, ILS remodeling could be physically linked to the binding of DBR1. It is also conceivable that the YJU2B interaction with SYF1, as seen in the ILS^{AQR} structure, could influence the accessibility of the C19L1 MPP domain interface for DBR1 binding (Fig. 2e), providing an additional means for promoting DHX15 activation and ILS^{AQR} remodeling.

Further experiments will be needed to test these models. To remain conservative, we have added a clarifying statement in the legend of the updated Extended Data Fig. 5f (Lines 1888-1892) regarding the possible implications of the two similar DBR1 and SYF1 peptides.

7. Line 252: "Although we did not observe density for the late termination factors WDR83, GPATCH1 and DHX35 in the ILSAQR…" This statement implies that these factors are present in the ILSAQR complex but cannot be observed due to limitation in EM density. However, earlier biochemical data seem to indicate that these factors are not part of the complex. If this is correct, please rephrase the statement to be more accurate.

The reviewer is correct that the factors WDR83, GPATCH1 and DHX35 are not detected by mass spectrometry in the gradient-purified ILS^{AQR} sample used for cryo-EM. We did however detect these proteins in the YJU2B-GFP pulldown by mass spectrometry at approximately 3-fold lower levels than ILS-specific factors, prior to the gradient purification step (Extended Data Fig. 1g and response to reviewer 1 question #8, Response Fig. 2.1). This suggests that these factors may weakly associate with the ILS^{AQR}. We have rephrased this sentence to clarify that WDR83, GPATCH1 and DHX35 are absent from the imaged cryo-EM sample of the ILS^{AQR} (Lines 263-266):

"Although the late termination factors WDR83, GPATCH1, and DHX35 were not detected in our final purified ILS^{AQR} sample, low levels of these proteins were present prior to gradient purification, indicating a transient association (Extended Data Fig. 1g)

8. The authors propose that YJU2B guides ILSAQR remodeling by recruiting DHX35, GPATCH1, and WDR83. The in vitro pulldown data using purified proteins support this hypothesis. Since the authors have GFP tagged YJU2B, do these proteins associate with YJU2B by mass spectrometry or Western blot analyses of the GFP-YJU2B sample? This would provide strong evidence that these interactions indeed occur in cells.

As briefly described in our response to comment #7, we did detect DHX35, GPATCH1, and WDR83 in the YJU2B-GFP pulldown by mass spectrometry, shown in the heatmap in Extended Data Fig. 1g and now also in Response Fig. 2.1 (see below). These DIS-specific factors were approximately 3-fold less abundant than the ILS-specific termination factors (excluding the bait protein YJU2B, which was super-stoichiometric), suggesting that they associate weakly with the ILS^{AQR}, possibly via the YJU2B–WDR83 interaction we report in vitro (Extended Data Fig. 6e). In Extended Data Fig. 1g, we had normalized the abundance of each protein in the YJU2B pulldown to the abundance of the core spliceosome factor PRP8 to report the stoichiometry of various factors on the spliceosome. When we re-plotted the same data using the non-normalized abundance values of the various proteins on a log-scale (Response Fig. 2.1), the association of DHX35, GPATCH1, and WDR83 with YJU2B-GFP is more apparent. As pointed out in response to comment #7, these DIS-specific proteins are not present in the peak sucrose gradient fractions that were used to prepare the cryo-EM sample of the ILS^{AQR}. These data indicate that YJU2B can associate with DHX35–GPATCH1–WDR83 in cells.

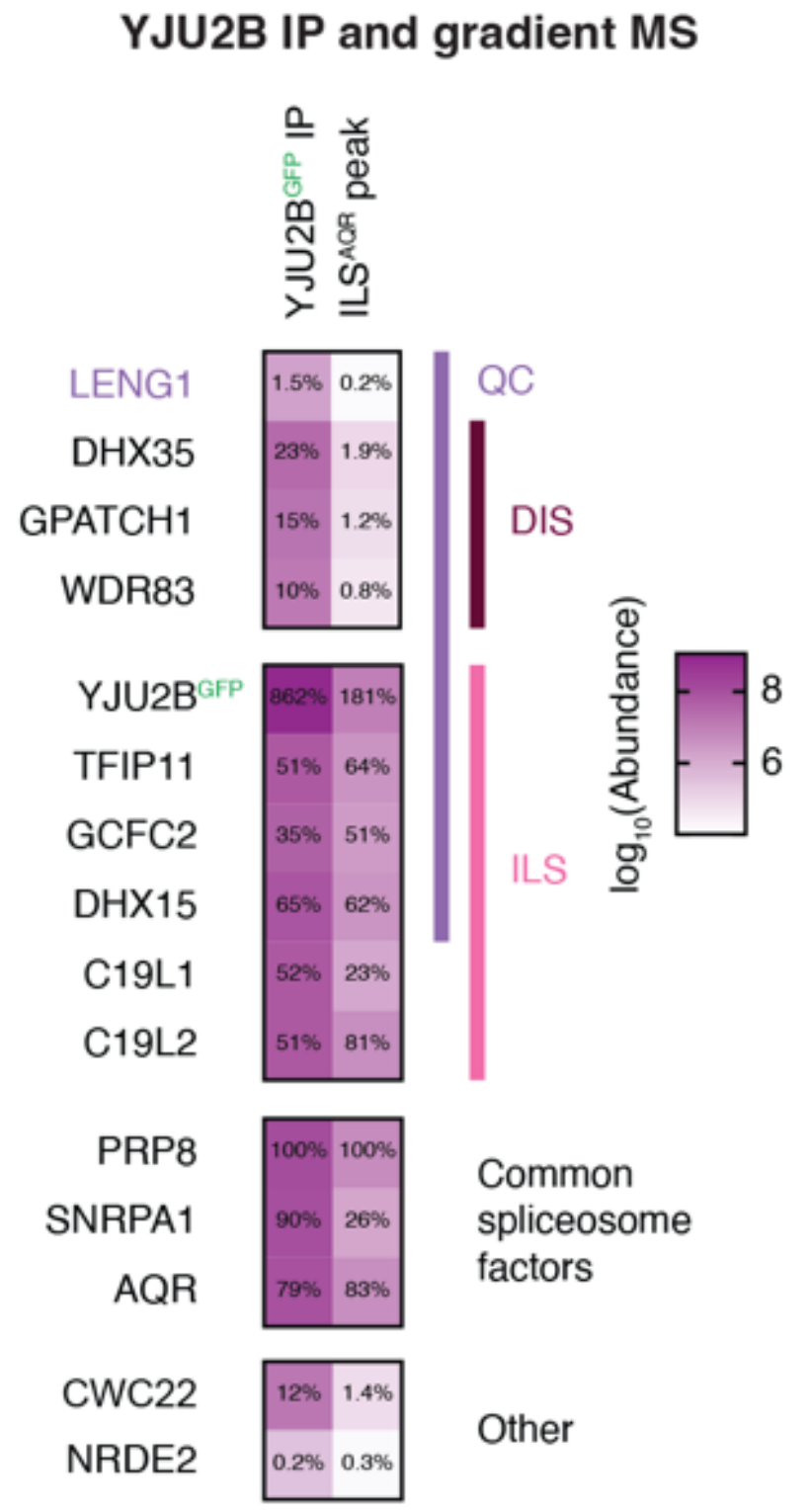

Response Fig. 2.1 | DHX35-GPATCH1-WDR83 bind YJU2B in cells. *Heatmap representing non-normalized abundances of relevant spliceosome factors in the native YJU2B-GFP pulldown before (YJU2B^{GFP} IP) and after sucrose gradient purification (ILS^{AQR} peak). The stoichiometry of each protein in both samples relative to the core spliceosome component PRP8 is indicated in each box. The exact same panel is also shown in Response Fig. 1.5a.*

9. In Fig. 3g-h, RNAseq is used to assess the branching status of introns in DIS. It would be helpful to include information on the quality of the RNAseq data (e.g., total number of reads) and the number of introns used for these aggregated plots. This would strengthen confidence in the comparison and support the conclusion that the intron in DIS is debranched.

Thank you for this suggestion. For the revision, we have established a new RNA sequencing assay, which provided a highly improved sequencing depth of both ILS^{AQR} and DIS associated RNAs and for the analysis of reads truncating at the intron branch point (new Fig. 3f-i). The results from this new assay are fully consistent with the earlier sequencing assay (now in Extended Data Fig. 7d-f), showing that introns associated with the DIS derive predominantly from debranched and excised introns. For both assays, we now include the number of total reads and number of individual introns in the figure legend of each plot (figure legends of Fig. 3 and Extended Data Fig. 7).

Referee #3 (Remarks to the Author):

I co-reviewed this manuscript with one of the reviewers who provided the listed reports.

We thank the reviewer for their helpful comments, which helped us to improve our manuscript.

Referee #4 (Remarks to the Author):

“Mechanism of Spliceosome Termination” by Boreikaite et al appears to be a rigorous and fairly complete study of spliceosome termination. The work defines two late spliceosomal states (ILSAQR and DIS) and then uses cryo-EM and proteomic and genomic approaches study the sequence of intron-lariat extraction, debranching and intron ejection. The paper is well written and the introduction does a good job laying out the problem. Using pulldown/mass-spec on GFP tagged late-stage factors revealed distinct spliceosomes. The authors technique was very good - The subsequent cryo-EM resulted in the highest quality spliceosomal structures achieved to date.

Not a lot is known about the end of the spliceosomes life cycle and this manuscript covers a lot of ground discovering and characterizing the DIS and establishing at least some cases DBR1 of acting before the final release. My overall impression was this is a very significant work that will make a contribution to our understanding of this important step in gene expression.

We thank the referee for the helpful comments and the support of our manuscript.

CONCERNS
Does the existence of two late stage spliceosomes necessarily mean that one precedes the other? Is DIS the terminal complex for all introns? Could there not be more than one way to terminate? The clip data for AQR is quite good but it shows many introns don’t have significant clip enrichment. Is it possible that heterogeneity in late complexes reflect the existence of an AQR bound and unbound fraction? I think there is evidence that AQR does help debranching because Buerer et al (ref 14) shows lariats 1) go up when AQR is knocked down, 2)have a branchpoint signature consistent with DBR1 avoidance. However they find a strong cis-effect - introns enriched for AQR CLIP signal are more susceptible to this loss DBR1. To me, this begs the question: “do all DIS complexes start as ILSAQR?”. If the answer is no, how universal is this model (i.e. what fraction go through ILSAQR)?

I believe the sequencing was only used to verify introns and lariat/linear status. It might be worthwhile to re-analyze or repeat sequencing of the RNA in complexes to demonstrate the same introns pass through both stages. If the data is too sparse for comparison, introns could be cross-checked for AQR CLIP enrichment in the introns in ILSAQR and introns in the DIS. I believe this is a relatively straightforward way to

explore the generality of the model and I don't think it would lessen the finding if the authors found alternate pathways.

We thank the reviewer for this insightful comment. To address whether the ILS-to-DIS progression is a general feature of the splicing pathway, we established a new RNA sequencing experiment for this revision. In this experiment, we could sequence the ILS- and DIS-associated introns with much greater depth, enabling a detailed comparison of the introns bound in both complexes.

This assay focused on the analysis of the reads truncating at the intron branch point (new Fig. 3f-i), which is the case for introns containing a branched intron-lariat (in the ILS^{AQR}) but not for introns that have been debranched (observed in the DIS). We specifically focused on the intron reads mapping to the 3'-splice site and representing excised introns. Due to the increased depth of the new sequencing data, we were able to calculate the fraction of detectable and excised introns (making up 89,807 unique introns, representing ~40% of all introns in human genome) among ILS-associated introns, which also have reads mapping to the 3'-SS in the DIS sample. To account for the ILS data being significantly deeper than the DIS data (around 20-fold more intron-mapping reads), owing to the different spliceosome purification yields, we performed this calculation after filtering the ILS intron set based on their read count percentile as a proxy for intron abundance and its detection sensitivity in the samples. Even without filtering, 75% of introns associated with the ILS^{AQR} are also associated with the DIS sample (Response Fig. 4.1). When we filter for the top half of introns detected in the ILS^{AQR} intron set by read abundance, 92.4% of those introns are observed in the DIS (new Fig. 3i), and for the most abundantly detected ILS introns this approaches 100% (Response Fig. 4.1). Conversely, more than 99% of introns associated with the DIS are also observed in the ILS^{AQR} (Response Fig. 4.1). These results strongly suggest that DIS complexes do not form on specific subsets of introns independent of the ILS pathway, but rather that the ILS-to-DIS progression is a general feature of the pre-mRNA splicing pathway. We thus find no evidence for alternative disassembly pathways for the 89,807 detected unique introns, although we cannot exclude them for lowly expressed introns below our RNA sequencing detection threshold.

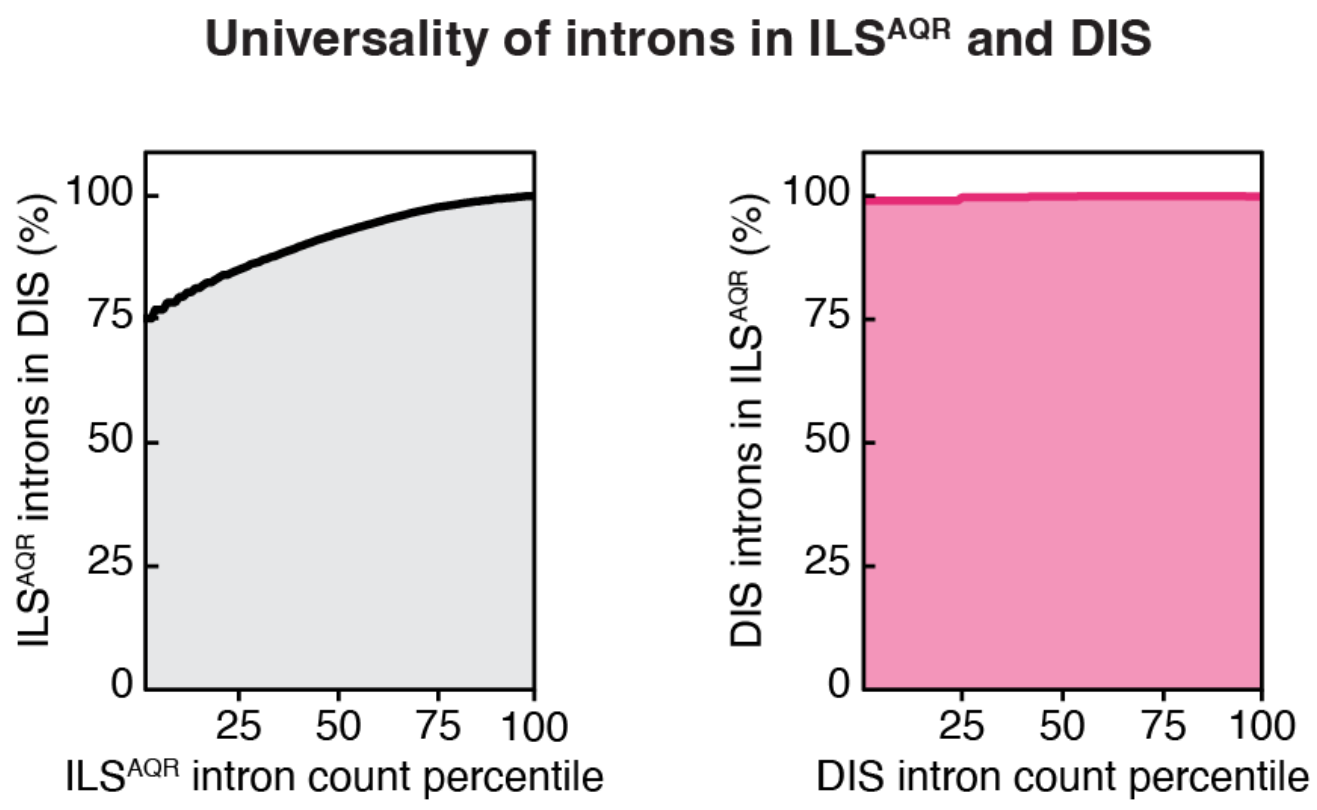

Response Fig. 4.1 | Evidence for a sequential and universal transition between ILS^{AQR} and DIS. *Plots showing the percentage of ILS^{AQR} introns of various abundance levels present in DIS (left) and the percentage of DIS introns of various abundance levels present in ILS^{AQR}. A count percentile of 0 corresponds to the inclusion of every intron in the dataset, while a percentile of 99 corresponds to only the 1% most covered introns in the dataset. The same data are presented in Response Fig. 1.4.*

These RNA sequencing data are further supported by biochemical evidence. First, in the submitted manuscript we showed that ATP treatment of C. elegans ILS spliceosomes leads to the depletion of ILS-specific factors and accumulation of DIS-specific factors with PRP19-containing complexes (Extended Data Fig. 1a). Second, we now show for the revision that the ATP-dependent remodeling of the human ILS^{AQR} leads to the specific binding of human GPATCH1–DHX35 to ATP-treated spliceosomes in human nuclear extract (new Extended Data Fig. 7a,b), consistent with the ILS^{AQR}-to-DIS transition. Third, these observations also agree with previous work showing that a 35S snRNP complex, which closely resembles the DIS in protein and snRNA composition, including the absence of U2 snRNP, and which accumulates at the end of in vitro splicing reactions (Makarov et al., 2002, Wahl et al, Cell, 2009; Science; Makarova & Makarov, PLOS One, 2015). These three lines of evidence support a sequential transition of ILS-to-DIS.

Regarding an analysis of AQR CLIP data from Beurer et al. 2024, we believe that the direct comparison of intron overlap provided from the new RNA sequencing assay and resulting analysis (Fig. 3i) provides a more comprehensive test of generality of the ILS^{AQR} and DIS complexes in spliceosome termination. Further analysis of the AQR CLIP data would be complicated by several factors, including (i) intron abundance/expression-level biases in CLIP signal detection, (ii) the presence of AQR on the spliceosome from the pre-B^{act} complex onwards, resulting in the

convolution of multiple spliceosome states in the AQR CLIP data, and (iii) the use of different cell lines. We wish to also note that based on our analysis of the ILS and DIS cryo-EM data, AQR appears to be bound to the spliceosome in all particles, and bound to RNA in all ILS^{AQR} particles.

Together, these combined data support the generality of the ILS^{AQR}-to-DIS transition.

Finally I would be cautious with using strong, definitive language describing conclusions that lack direct evidence. A real strength was the use of structure, biochemistry and site directed mutagenesis to return mechanistic detail like their characterization of DBR1 docking to ILSAQR. Perhaps unavoidably, not all sections enjoyed this level of experimental support. Earlier on Pg8 contains a lot of speculation based on known roles of factors in other steps of splicing cycle. I acknowledge that the many difficulties in working with and even identifying labile RNA species such as lariats may place experimental follow up beyond the scope of this work. However the more these assumptions are combined into models of concerted actions (e.g. AQR and DHX15 pulling the RNA complexes apart) the more I worry about proteins acting outside their previously characterized functions. The author may want to devise experiments to confirm mechanism or consider language that leans more towards discussion rather than the presentation of a result.

Similar concerns for statements like “YJU2B guides ILSAQR remodeling” and the last few sections.

We thank the reviewer for this important comment. We agree with the reviewer that aspects of the mechanistic models on page 8 are inferred from our structural data and remain challenging to address experimentally. For the revision we have therefore revised the language of this and other sections pointed out by the reviewer in order to remain conservative and to make this distinction more explicit.

The structural models and known biochemical activities from other contexts allow us to propose how AQR and DHX15 may contribute to ILS^{AQR} remodeling, but we now clearly discuss that these roles are an interpretation of our structural data and are inferred from the positioning of these proteins within the complex. For example, we have changed “Translocation of AQR along the intron-lariat in the ILS^{AQR} is thus poised to unwind the U2-intron branch helix and pull on the intron-lariat BP adenosine” to “Our structure suggests that translocation of AQR along the intron-lariat in the ILS^{AQR} would be thus poised to unwind the U2-intron branch helix and

pull on the intron-lariat BP adenosine" to keep the phrasing conservative. We have also changed the title of the section "YJU2B guides ILS^{AQR} remodeling" to "YJU2B may guide ILS^{AQR} remodeling" (Line 248). We have adjusted the language in the sections "Recognition of defective spliceosomes for termination" and "Mechanisms of regular and defective spliceosome termination" to more clearly distinguish observations from speculation. We believe that these models derived primarily from the structural data remain valuable as a framework for future experiments.

Minor points

Line 33. I am a little uncomfortable referring to the spliceosome as a "single turnover enzyme" because as you mentioned the components can be recycled and can we say for sure that the distinct complexes (i.e. A, B, C etc) described in the literature don't remain loosely attached throughout the whole process.

We agree with the reviewer and have removed this description from line 37 in the revised manuscript.

The paper localizes AQR to 40 nt upstream of 3'ss. AQR binds functionally both on the 5'ss and 3'ss of the branchpoint (CLIP studies from Yeo lab and Buerer et al)

Thank you for pointing this out. As indicated by the studies cited by the reviewer, AQR could bind on either side of the intron branch point. However, within the ILS^{AQR} complex, the arrangement of the RNA network and the relative position of AQR constrains AQR to bind only upstream of the branch point. This is because of the orientation of the intron-lariat relative to AQR and because the intron's 5′-splice site region is base paired with an extended region of U6 snRNA. To reflect the prior literature more precisely, we have revised the sentence to (Line 151-153):

"Based on prior reports of AQR binding to an intron region ~40 nucleotides on either side of the annotated BP[14,27,28] and the relative positions of the RNA elements within the ILS^{AQR} structure, we assigned the AQR-bound RNA as the circular portion of the intron-lariat."

List of responses to reviewer comments

"Mechanism of spliceosome termination"

(Vytaute Boreikaite, Rupert Faraway, Matthias K. Vorländer, Alexander W. Phillips, Leonie Opitz, Moritz Wanke, George Yakoub, Gerald Raffl, Laura Fin, Román González-Prieto, Martijn S. Luijsterburg, Stefan L. Ameres, and Clemens Plaschka)

Nature Manuscript 2025-08-22717

Referee expertise:
Referee #1: cryo-EM, splicing
Referee #2: cryo-EM, splicing
Referee #3: co-review with referee #2
Referee #4: splicing, biochemistry

Referee comments are in blue
Responses are in *black*

Referees' comments:

Referee #1 (Remarks to the Author):

The authors have made a substantial revision that addresses most of the major concerns raised in my original review. In particular, the new RNA-sequencing analysis and human remodelling experiments strengthen the proposed relationship between the ILSAQR and DIS states, corroborates the evidence that excised introns associated with DIS are mostly debranched, and supports the role of DHX35 in late spliceosome disassembly. The additional experiments addressing LENG1 also improve the connection between canonical termination and spliceosome quality control.

The authors have dealt satisfactorily with the issues surrounding the ILSAQR structure. They now place it more carefully in the context of previous ILS structures and prior suggestions that AQR/PPIE participate in disassembly. The presence of YJU2B, rearrangement of C19L1 and, most importantly, the improved RecA domain conformation analysis strongly supports engagement of intron RNA by AQR. Together they make a reasonable case that ILSAQR represents a distinct later state rather than simply a higher-resolution version of ILS2.

The revised active-site density representations similarly make the DIS interpretation clearer. More importantly, the new sequencing experiment is a substantial improvement. By restricting the branch-point analysis to reads whose 3′ end maps to the 3′ splice site, the authors specifically analyse excised introns, and the difference in branch-point truncation between ILSAQR and DIS provides convincing evidence that this population of DIS-associated introns is predominantly debranched. The extensive overlap between introns detected in ILSAQR and DIS also provides much better evidence than in the original submission that these complexes are related states in a common termination pathway.

I also commend the authors for the new human remodelling assay. ATP treatment causes loss of ILS-specific factors, while DHX35–GPATCH1 preferentially associates with the remodelled complex, providing direct biochemical evidence for progression from ILSAQR towards a DIS-like state. The authors now also acknowledge that additional short-lived intermediates may exist, which I think is both more realistic and better supported by their own data. The evidence for DHX35-mediated disassembly has likewise been strengthened by showing that U5 and U6 snRNAs, as well as spliceosomal proteins, are depleted following ATP treatment. Although the precise RNA substrate of DHX35 remains inferred rather than directly demonstrated, the manuscript now generally distinguishes this mechanistic model from direct observation more carefully.

The new LENG1 experiments also address an important concern from the original review. The LENG1 pulldown recovers PRP8, CWC22, DHX35, GPATCH1, WDR83 and YJU2B, providing biochemical evidence that a B*D-like complex can exist in human cells. The revised discussion also more clearly explains why DHX15 and DHX35 may occupy sequential states during canonical termination but coexist in defective spliceosomes.

One important issue from my original review, however, is not in my view completely resolved. I had questioned whether the DHX35-associated DIS sample necessarily represents a single canonical post-splicing state, or whether it could contain a mixture of late disassembly and rejected spliceosomal complexes. The revised sequencing actually makes it important in my view to explicitly consider this possibility.

The authors report that approximately 30% of reads in the DIS sample are exonic, compared with approximately 7% in ILSAQR, and identify a substantial population of reads extending between intron and downstream exon sequences. They analyse these reads and find no branch-point truncation signature, which they interpret as evidence that they derive from pre-mRNAs that have not undergone branching, thereby arguing against a significant contribution from lariat-intermediate-containing complexes.

I am not convinced that this conclusion follows uniquely from the analysis. A lariat-3′-exon intermediate that had undergone branching and was subsequently debranched by DBR1 would also lack a branch-point signature while retaining continuity between the intron and downstream exon. Thus, absence of a branch-point RT-stop distinguishes branched from unbranched RNA, but as far as I can see does not distinguish an unspliced pre-mRNA from a debranched lariat intermediate. The latter would correspond to a spliceosome that had undergone the first catalytic step of branching in the B*/C complex and was subsequently rejected before exon ligation.

This population is significant: the authors analyse 68,724 intron-exon reads from 23,919 intron-exon pairs in the DHX35 purification. Indeed, the revised manuscript itself now notes that DHX35-associated RNA includes intron-exon junction reads and interprets this as evidence that DHX35 can associate with potentially aberrant substrates as well as successfully excised introns. This seems entirely compatible with my original suggestion that DHX35 may participate in a more general terminal disassembly mechanism acting on both canonical and rejected spliceosomes.

I do not think this undermines the structural characterization of the predominant DIS state, nor the conclusion that the majority of excised DIS-associated introns are debranched. The cryo-EM structure itself lacks 5′-exon density and can reasonably represent a canonical post-splicing DIS. However, I think the biochemical DHX35 purification should be treated more explicitly as potentially heterogeneous, and the sequencing does not exclude the possibility that part of the population consists of debranched, post-branching rejected complexes.

I would therefore ask the authors to address this point explicitly in the text. I do not think additional experimentation is necessary, but they should avoid implying that absence of a branchpoint signature proves that the intron-exon reads derive exclusively from pre-mRNAs that had not undergone the first catalytic step. They should acknowledge the alternative possibility of debranched post-branching intermediates and, correspondingly, that DHX35-containing DIS-like states may be reached from more than one spliceosomal intermediate.

Overall, the revision has substantially strengthened the manuscript and most of my original concerns have been addressed. The remaining issue is primarily one of

interpretation and appropriately defining the heterogeneity of the DHX35-associated population. Thus, subject to clarification of this point, I support publication in Nature.

We thank the reviewer for their support and for raising this point. As the reviewer notes, the absence of a stop at a branch point during reverse transcription discriminates branched from unbranched RNAs, but not unspliced pre-mRNAs from lariat intermediates that underwent branching and were subsequently debranched. It thus remains possible that aberrant spliceosomes arrested after branching, and whose introns were then debranched, are captured in the native DHX35 purification. We have now revised the text to state this limitation explicitly:

Page 16, Lines 347-351: "The native DHX35 purification could thus be heterogeneous. While the regular DIS represents the predominant state, DIS-like states could have arisen from defective spliceosomes stalled either before, as in the B^{*D}, or after branching. The roles of these SQC factors, and the full scope of states they target, await further investigation."

Page 81, Lines 1841-1843: "We cannot exclude that a fraction of particles in our DIS cryo-EM reconstruction originates from SQC complexes that assembled prior to pre-mRNA branching or after branching and debranching."

Referee #2 (Remarks to the Author):

The authors have adequately addressed all of my concerns in the revised manuscript.

We thank the reviewer for this positive assessment.

Referee #3 (Remarks to the Author):

I co-reviewed this manuscript with one of the reviewers who provided the listed reports.

We thank the reviewer for co-reviewing the manuscript.

Referee #4 (Remarks to the Author):

The authors made changes that improved the manuscript. I still have some concern about overinterpreting the data. For example, I feel the passage "sequential passage of introns through ILS^AQR and DIS states is a universal feature of splicing" could be softened to read something like " …. is a predominant pathway for spliceosomal disassembly" . Describing the transition as a universal feature of splicing may cause confusion because of the name DIS (Debranched Intron Spliceosome) implies that all introns are debranched while they are still in the spliceosome. Figure 3g suggests ~ 20% introns in DIS are still branched. It probably makes sense to avoid such definitive language. The cis-effect of AQR I mentioned in my original review suggests the functional requirement for AQR in facilitating debranching is not uniform across introns. The possibility that there could be other ways to expose the branchpoint to DBR1 should be discussed as a potential caveat.

We thank the reviewer for these comments and agree that our original phrasing was overstated. As written, it implied that the generality of the ILS-to-DIS transition extended to debranching, and we have therefore edited the text to the following:

Page 12, Lines 266-268: "the vast majority of ILS^{AQR}-associated introns were detected in the DIS sample (Fig. 3i), indicating that sequential passage through ILS^{AQR} and DIS is the predominant spliceosome termination pathway."

As the reviewer notes, a subset of DIS-associated introns remain branched (Fig. 3g, Extended Data Fig. 7e). We therefore concluded that debranching largely occurs on the spliceosome, but that it is not a prerequisite for the ILS^{AQR}-to-DIS transition (as noted on page 12, Lines 258-261). The name DIS accordingly reflects the predominant state of the complex. We also agree with the reviewer that the requirement for AQR in debranching is unlikely to be uniform across all introns and that AQR-independent routes for exposing the branch point to DBR1 may exist. We have therefore added the following:

Page 8, Lines 156-157: "The dependence on AQR likely varies across introns[14], and we do not exclude AQR-independent routes to BP exposure for debranching."

Page 12, Lines 261-262: "These branched introns may represent complexes in which debranching is delayed or AQR independent.
