# Supplementary material for: Mechanism of spliceosome termination
Source: Nature. 2026 Oct 7;659(8139):248–55. doi: 10.1038/s41586-026-11101-6 (PMC13736545; doi:10.1038/s41586-026-11101-6)
Supplement: Supplementary file 4 [file 41586_2026_11101_MOESM3_ESM.zip › 2025-08-22717B-s3/boreikaite-SupplementaryDataTable3.pdf]

	Hs **ILS^{AQR}**	*Hs* **DIS**	*Sp* **B^{*D}**
**Data collection**			
Magnification	130,000	130,000	
Voltage, kV	300	300	
Total dose, $e^-/Å^2$	40	50	
Pixel size, Å	0.951	0.951	
Defocus range, μm	-1.00 to -2.50	-1.25 to -2.25	
**Coordinate model statistics**			
**Model composition**			
Protein residues	12,306	10,826	11,679
Nucleotide residues	291	137	248
Ligands	2	2	2
**Refinement (PHENIX)**			
Map CC (aroundatoms)	0.6226	0.6559	0.5511
RMS deviations			
Bond lengths, Å	0.012	0.0125	0.012
Bond angles	1.172	1.230	1.262
Validation			
MolProbity score	1.19	1.07	1.46
All-atoms clash score	1.13	0.82	3.16
Rotamer outliers, %	1.60	1.41	0.16
C-beta deviations, %	0.00	0.02	0.02
**Ramachandran plot**			
Outliers, %	0.25	0.19	0.22
Allowed, %	3.36	3.06	4.85
Favoured, %	96.40	96.76	94.94
**Data deposition**			
PDB ID	**9S2E**	**9S2F**	**9S2G**
EMDB (composite map)	**EMD-54493**	**EMD-54494**	ref. (*23*)
