# Supplementary material for: Mechanism of spliceosome termination
Source: Nature. 2026 Oct 7;659(8139):248–55. doi: 10.1038/s41586-026-11101-6 (PMC13736545; doi:10.1038/s41586-026-11101-6)
Supplement: Supplementary file 4 [file 41586_2026_11101_MOESM3_ESM.zip › 2025-08-22717B-s3/Supplementary_table_legends.pdf]

SUPPLEMENTARY DATA TABLES

Supplementary Data Table 1 | Mass spectrometry data of the ILS disassembly assay in total nematode extract.

Abundances of proteins that remain associated with the bead-bound nematode *C. elegans* ILS in the presence or absence of ATP treatment are shown.

Supplementary Data Table 2 | Mass spectrometry data of the IPs from human cells.

Protein abundances in anti-GFP IPs using as baits wild-type DHX35, YJU2B, DBR1 or mutant DHX35 K83A are shown. Wild-type extract as a negative control is shown for each experiment. Protein abundances in wild-type DHX35 and DHX35 K83A IPs were compared in the same experiment. The DBR1-GFP pulldown was performed in triplicates, allowing to derive p-values of protein enrichment. Data from a single experiment is shown for the rest of the bait proteins, but the results of these IPs were reproduced at least twice independently for each bait.

Supplementary Data Table 3 | Cryo-EM data collection and model refinement statistics.

Supplementary Data Table 4 | Mass spectrometry data of the proximity labeling experiments in human cells.

Biotin proximity labelling experiments of TurboID-tagged DBR1, C19L1, YJU2B, and LENG1 were performed in technical triplicates in parallel with a TurboID-tagged nuclear GFP control.

Supplementary Data Table 5 | Mass spectrometry data of the human DIS release assay.

Abundances of proteins that were released upon incubation with either ATP or ADP from the bead-immobilized human DIS containing either wild-type or catalytic mutant DHX35 were analyzed in technical triplicates. Some proteins were absent from at least one sample, most often from the ADP-treated samples. In these cases, to calculate a fold change of ATP over ADP samples, the abundance of a missing protein was imputed from the lower tail of the intensity distribution in that particular sample. If the protein was absent from all samples, for example, DHX35 in the wild-type DHX35 DIS samples, no fold change was calculated, resulting in blank cells in Extended Data Table 5.

Supplementary Data Table 6 | Mass spectrometry data of the LENG1-GFP IP.

Mass spectrometry data of the LENG1-GFP pulldown from human cells. The experiment was performed in quadruplicates, and the fold-change values compare the LENG1-GFP pulldown sample with the nuclear GFP control sample.
